# Hypertensive Disorders of Pregnancy and DNA Methylation in Newborns: Findings From the Pregnancy and Childhood Epigenetics Consortium

**DOI:** 10.1161/HYPERTENSIONAHA.119.12634

**Published:** 2019-06-24

**Authors:** Nabila Kazmi, Gemma C. Sharp, Sarah E. Reese, Florianne O. Vehmeijer, Jari Lahti, Christian M. Page, Weiming Zhang, Sheryl L. Rifas-Shiman, Faisal I. Rezwan, Andrew J. Simpkin, Kimberley Burrows, Tom G. Richardson, Diana L. Santos Ferreira, Abigail Fraser, Quaker E. Harmon, Shanshan Zhao, Vincent W.V. Jaddoe, Darina Czamara, Elisabeth B. Binder, Maria C. Magnus, Siri E. Håberg, Wenche Nystad, Ellen A. Nohr, Anne P. Starling, Katerina J. Kechris, Ivana V. Yang, Dawn L. DeMeo, Augusto A. Litonjua, Andrea Baccarelli, Emily Oken, John W. Holloway, Wilfried Karmaus, Syed H. Arshad, Dana Dabelea, Thorkild I.A. Sørensen, Hannele Laivuori, Katri Raikkonen, Janine F. Felix, Stephanie J. London, Marie-France Hivert, Tom R. Gaunt, Debbie A. Lawlor, Caroline L. Relton

**Affiliations:** 1 From the MRC Integrative Epidemiology Unit (N.K., G.C.S., A.J.S., K.B., T.G.R., D.L.S.F., A.F., M.C.M., T.I.A.S., T.R.G., D.A.L., C.L.R.), University of Bristol, United Kingdom; 2 Population Health Sciences, Bristol Medical School (N.K., G.C.S., K.B., T.G.R., D.L.S.F., A.F., M.C.M., T.R.G., D.A.L., C.L.R.), University of Bristol, United Kingdom; 3 School of Oral and Dental Sciences (G.C.S.), University of Bristol, United Kingdom; 4 Division of Intramural Research, Department of Health and Human Services, National Institute of Environmental Health Sciences, National Institutes of Health, Durham, NC (S.E.R., Q.E.H., S.Z., S.J.L.); 5 The Generation R Study Group (F.O.V., V.W.V.J., J.F.F.), Erasmus MC, University Medical Center Rotterdam, the Netherlands; 6 Department of Epidemiology (F.O.V., V.W.V.J., J.F.F.), Erasmus MC, University Medical Center Rotterdam, the Netherlands; 7 Department of Pediatrics (F.O.V., V.W.V.J., J.F.F.), Erasmus MC, University Medical Center Rotterdam, the Netherlands; 8 Department of Psychology and Logopedics, Faculty of Medicine (J.L., K.R.), University of Helsinki, Finland; 9 Helsinki Collegium of Advanced Studies (J.L.), University of Helsinki, Finland; 10 Institute for Molecular Medicine Finland, Helsinki Institute of Life Science (H.L.), University of Helsinki, Finland; 11 Division of Mental and Physical Health (C.M.P., W.N.), Norwegian Institute of Public Health, Oslo; 12 Centre for Fertility and Health (M.C.M., S.E.H.), Norwegian Institute of Public Health, Oslo; 13 Oslo Centre for Biostatistics and Epidemiology, Oslo University Hospital, Norway (C.M.P.); 14 Department of Biostatistics and Informatics (W.Z., K.J.K.), University of Colorado Anschutz Medical Campus, Aurora; 15 Department of Epidemiology (A.P.S., I.V.Y., D.D.), University of Colorado Anschutz Medical Campus, Aurora; 16 Department of Medicine (I.V.Y.), University of Colorado Anschutz Medical Campus, Aurora; 17 Department of Pediatrics (D.D.), University of Colorado Anschutz Medical Campus, Aurora; 18 Department of Population Medicine, Harvard Medical School, Harvard Pilgrim Health Care Institute, Boston, MA (S.L.R.-S., E.O., M.-F.H.); 19 Human Development and Health (F.I.R., J.W.H.), Faculty of Medicine University of Southampton, United Kingdom; 20 Clinical and Experimental Sciences (J.W.H., S.H.A.), Faculty of Medicine University of Southampton, United Kingdom; 21 Insight Centre for Data Analytics, National University of Ireland, Galway (A.J.S.); 22 Department of Translational Research in Psychiatry, Max-Planck Institute of Psychiatry, Munich, Germany (D.C., E.B.B.); 23 Department of Psychiatry and Behavioral Sciences, Emory University School of Medicine, Atlanta, GA (E.B.B.); 24 Research Unit of Gynaecology and Obstetrics, Department of Clinical Research, University of Southern Denmark, Odense (E.A.N.); 25 Center for Genes, Environment and Health, National Jewish Health, Denver, CO (I.V.Y.); 26 Channing Division of Network Medicine, Brigham and Women’s Hospital, Harvard Medical School, Boston, MA (D.L.D.); 27 Division of Pediatric Pulmonary Medicine, University of Rochester Medical Center, NY (A.A.L.); 28 Laboratory of Precision Environmental Biosciences, Columbia University Mailman School of Public Health, New York, NY (A.B.); 29 Division of Epidemiology, Biostatistics, and Environmental Health, School of Public Health, University of Memphis, TN (W.K.); 30 Novo Nordisk Foundation Center for Basic Metabolic Research, Section on Metabolic Genetics (T.I.A.S.), Faculty of Health and Medical Sciences, University of Copenhagen, Denmark; 31 Department of Public Health, Section on Epidemiology (T.I.A.S.), Faculty of Health and Medical Sciences, University of Copenhagen, Denmark; 32 Medical and Clinical Genetics, University of Helsinki and Helsinki University Hospital, Finland (H.L.); 33 Faculty of Medicine and Life Sciences, University of Tampere, Finland (H.L.); 34 Department of Obstetrics and Gynecology, Tampere University Hospital, Tampere, Finland (H.L.); 35 Institute for Molecular Medicine Finland, Helsinki Institute of Life Science, University of Helsinki, Helsinki. Finland (H.L.); 36 Diabetes Unit, Massachusetts General Hospital, Boston, MA (M.-F.H.); 37 NIHR Bristol Biomedical Research Centre, Bristol, United Kingdom (T.R.G., D.A.L., C.L.R.).

**Keywords:** cardiovascular diseases, DNA methylation, gestational age, hypertension, preeclampsia

## Abstract

Hypertensive disorders of pregnancy (HDP) are associated with low birth weight, shorter gestational age, and increased risk of maternal and offspring cardiovascular diseases later in life. The mechanisms involved are poorly understood, but epigenetic regulation of gene expression may play a part. We performed meta-analyses in the Pregnancy and Childhood Epigenetics Consortium to test the association between either maternal HDP (10 cohorts; n=5242 [cases=476]) or preeclampsia (3 cohorts; n=2219 [cases=135]) and epigenome-wide DNA methylation in cord blood using the Illumina HumanMethylation450 BeadChip. In models adjusted for confounders, and with Bonferroni correction, HDP and preeclampsia were associated with DNA methylation at 43 and 26 CpG sites, respectively. HDP was associated with higher methylation at 27 (63%) of the 43 sites, and across all 43 sites, the mean absolute difference in methylation was between 0.6% and 2.6%. Epigenome-wide associations of HDP with offspring DNA methylation were modestly consistent with the equivalent epigenome-wide associations of preeclampsia with offspring DNA methylation (R^2^=0.26). In longitudinal analyses conducted in 1 study (n=108 HDP cases; 550 controls), there were similar changes in DNA methylation in offspring of those with and without HDP up to adolescence. Pathway analysis suggested that genes located at/near HDP-associated sites may be involved in developmental, embryogenesis, or neurological pathways. HDP is associated with offspring DNA methylation with potential relevance to development.

## Abstract 2

Supplemental Digital Content is available in the text.

Hypertensive disorders of pregnancy (HDP), including gestational hypertension (GH) and preeclampsia, are among the most common complications of pregnancy.^1^ Previous studies have found associations between maternal HDP and the hematological profile of newborns,^2^ preterm birth,^3^ low birth weight,^4^ and slower growth patterns in early infancy.^5^ Associations of HDP with subsequent higher blood pressure (BP) and cardiovascular disease risk in offspring have also been reported.^6–8^ The mechanisms underlying these associations are not fully understood. It has been suggested that environmentally responsive, mitotically stable epigenetic phenomena, such as DNA methylation, may mediate some of the impact of intrauterine exposures, such as to maternal HDP, on subsequent health outcomes in offspring.^9^

Several small studies have found preeclampsia-associated differences in DNA methylation, other epigenetic markers, and gene expression in placental tissue,^10–14^ maternal gestational blood cells, and maternal omental fat biopsies collected during gestation.^15,16^ Two previous studies have explored the association of HDP with DNA methylation in offspring cord blood.^17,18^ Previous studies included small number of preeclampsia cases, reported few findings that reached genome-wide level of confidence, and findings were not replicated. Larger studies would enable replication of these findings to be tested and would likely identify novel loci.

We hypothesized that HDP may influence DNA methylation patterns in cord blood in genes that are important for fetal development. To address this, we examined the association of HDP (preeclampsia and GH jointly), with epigenome-wide DNA methylation in cord blood by meta-analyzing results from 10 birth cohorts from the pregnancy and childhood epigenetics consortium. Our main analyses focused on HDP, and in additional analyses, we also explored epigenome-wide differential methylation between cases of preeclampsia and controls (without any form of HDP). We performed pathway analyses to explore the underlying biology of differentially DNA methylated sites for HDP and preeclampsia.^19^ We also investigated whether the differentially methylated CpG sites (CpGs) identified in relation to HDP in cord blood change over time in serial samples taken from the same individuals in childhood and adolescence (as well as cord blood) using data from 1 cohort.

## Methods

### Data Availability

Data supporting the results reported in this article can be found in the online-only Data Supplement. We are unable to make individual-level data available because of concerns about compromising individual privacy; however, data used in these analyses are available from the individual cohorts on request. Full meta-analysis results datasets generated in this study are available from the corresponding author (nabila.kazmi@bristol.ac.uk) on request.

### Cohort Participants

Our a priori primary analysis was of the association of HDP with epigenome-wide DNA methylation in cord blood. This provided maximal statistical power by enabling us to include all studies, including those that did not have sufficient numbers of preeclampsia for meaningful analyses or had not collected data that could differentiate GH from preeclampsia. In secondary analyses, we examined associations of preeclampsia only with DNA methylation in cord blood.

All pregnancy and childhood epigenetics consortium studies were invited to participate, women had to have experienced a singleton pregnancy, and only those studies with >10 cases of HDP were included in the main analyses. Similarly, for the secondary analyses of preeclampsia, only studies with >10 cases of preeclampsia occurring in singleton pregnancies were included.

Ten cohorts were included in the meta-analysis of HDP and cord blood methylation. These cohorts listed in alphabetic order were the following: the ALSPAC (Avon Longitudinal Study of Parents and Children),^20–22^ the GenR (Generation R) study,^23,24^ the GOYA (Genetics of Overweight Young Adults) study (a genome-wide population-based association study nested in the Danish National Birth Cohort),^25,26^ healthy start (Hispanic and non-Hispanic),^27,28^ the Isle of Wight cohort,^29^ 2 independent datasets from the MoBa (Norwegian Mother and Child Cohort Study; MoBa1 and MoBa2),^30,31^ The PREDO (Prediction and Prevention of Preeclampsia and Intrauterine Growth Restriction),^32^ and Project Viva.^33^

MoBa (MoBa1 and MoBa2) and PREDO also had sufficient number of cases (n>10) to contribute to the meta-analysis of the specific association of preeclampsia with cord blood DNA methylation.

### Meta-Analyses

We used inverse variance-weighted fixed effects meta-analysis performed in METAL^34^ to pool results across studies. This method summarizes effect sizes from several independent studies by calculating the weighted mean of the effect sizes using the inverse variance of the individual studies as weights.^35^ A fixed-effect meta-analysis assumes that all studies are from the same underlying population, and the pooled results reflect the association in that population. We a priori assumed that the studies contributing to this collaboration were from the same underlying population. However, we explored heterogeneity between studies for adjusted results that reached our prespecified Bonferroni level of statistical significance using Cochran Q statistic and by undertaking leave-one-out analyses; the latter using metafor in the R statistical package.^36^ These analyses test our assumption that results are from the same underlying population and act as a test of replication. If results are consistent across studies (ie, the *Q* test supports the null hypothesis of no heterogeneity, and results of meta-analyses that remove one of the studies at a time are consistent with each other), this shows replication across the different studies included in our meta-analysis. We considered the fully adjusted models (adjusted for confounders, cell proportions, and technical covariates [batch effects]^37^; each cohort adjusted for technical covariates using methods suitable for that cohort [online-only Data Supplement]) as our primary analyses but also reported the results of an unadjusted model. The sample size was the same for adjusted and unadjusted models: HDP (n=5242, cases=476) and preeclampsia (n=2219, cases=105). We highlighted results from the meta-analysis that reached statistical significance based on a false discovery rate of 5% and also with a more stringent Bonferroni-corrected *P* threshold (*P*<1.05×10^−7^; correcting for 475 089 tests for HDP analysis and 473 862 tests for preeclampsia analysis). The CpGs passing the more stringent Bonferroni-corrected *P* were considered our primary findings.

### Differentially Methylated Region Analysis

In addition to EWAS (epigenome-wide association study) analyses, differentially methylated region (DMR) analyses in relation to HDP and preeclampsia were conducted separately using the R package DMRcate.^38^ In the DMR analysis, the results of fully adjusted meta-analyzed models were used. DMRcate groups associated probes into separate DMRs if the gap between nucleotides is ≥1000 base pairs and the Bonferroni-corrected *P* of associations <0.05.

### Longitudinal Analysis of HDP and Offspring Methylation

Longitudinal analyses were performed to examine whether the associations of HDP on offspring differential DNA methylation at CpGs at birth were observed at the same CpGs in blood cells at age ≈7 years (mean age, 7.5 years; SD, 0.1) and at age ≈17 years (mean age, 17.1 years; SD, 1.0). These analyses were conducted in 1 cohort only (ALSPAC) and were restricted to the Bonferroni significant CpGs from the main HDP EWAS. Multilevel models were used, level 1 being the repeat assessments (at birth, 7 years, or 17 years) and within participants (level 2).^39,40^ The CpGs were first linked to the gene symbols using an Illumina mapping file and if unsuccessful were annotated to the nearest gene within 10 Mb of each CpG. A multilevel model including a random intercept (to allow for between individual variability in methylation) and a linear spline term (to allow for nonlinear change in methylation from birth to 17 years) was fitted to each of these CpGs. This model is described in more detail in the online-only Data Supplement. These longitudinal analyses included 658 participants in whom 108 had been exposed to HDP.

### Additional Information

Additional information (definition of HDP, covariates, methylation measurements and quality control, estimation of cell-type proportions, CpG annotation, statistical analysis methods, and some results) is provided in online-only Data Supplement.

## Results

### Study Characteristics

A total of 10 independent cohorts participated in our meta-analysis of HDP and DNA methylation of cord blood. The total number of newborns was 5242, including 476 whose mothers met our criteria for HDP (cases of GH+preeclampsia). Of those 10 cohorts, 3 contributed to the preeclampsia analyses (n=135 cases; Table 1). Each cohort is described in detail in Table S1.

**Table 1.**
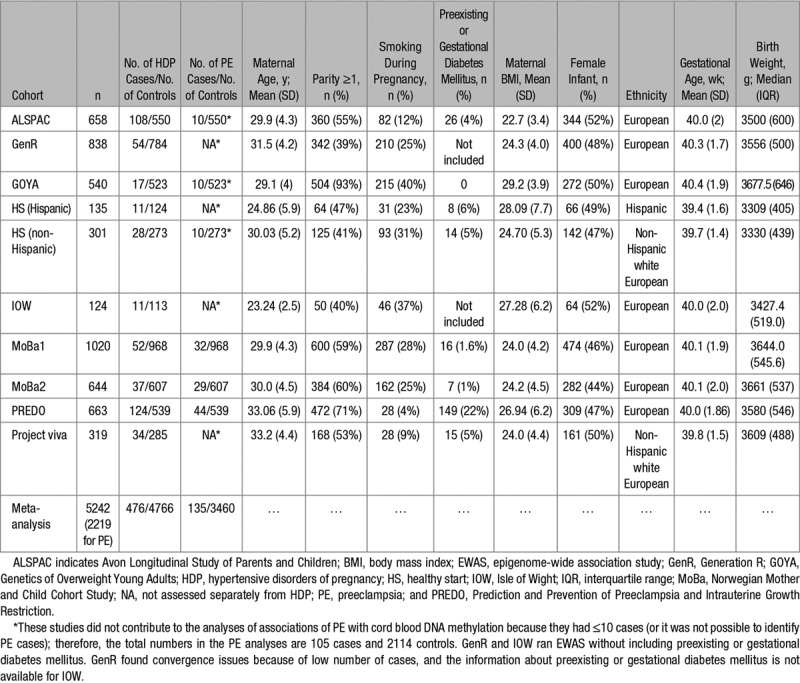
Summary of Cohorts Participating in the Meta-Analysis of HDP and DNA Methylation at Birth

### Main Meta-Analyses Results

In the EWAS meta-analysis evaluating the association between HDP and differential DNA methylation in cord blood, after adjustment for confounders, estimated cell counts, and technical covariates, we found 43 differentially methylated sites that passed the Bonferroni-corrected *P* (*P*<1.05×10^–7^) threshold (Figure; Table 2; Table S2). HDPs were associated with higher methylation levels at 27 (63%) of the CpGs, and across all CpGs, associations were relatively weak with the range of mean difference in methylation being 0.6% to 2.6%. In unadjusted meta-analysis of the association of HDP with cord blood DNA methylation, 70 CpGs reached the Bonferroni *P* (Table S3) including 12 of the 43 CpGs identified in adjusted analyses. When we directly compared the direction and size of association in the 2 EWAS of HDP (unadjusted EWAS versus fully adjusted EWAS), we found moderate consistency between the two (R^2^=0.48, slope=0.854±0.001; Figure S1). When we used a 5% false discovery rate method to account for multiple testing (rather than Bonferroni correction), a much larger number of CpGs (n=1075) were shown to be differentially methylated in relation to HDP in the fully adjusted model (Figure; Table S2).

**Table 2.**
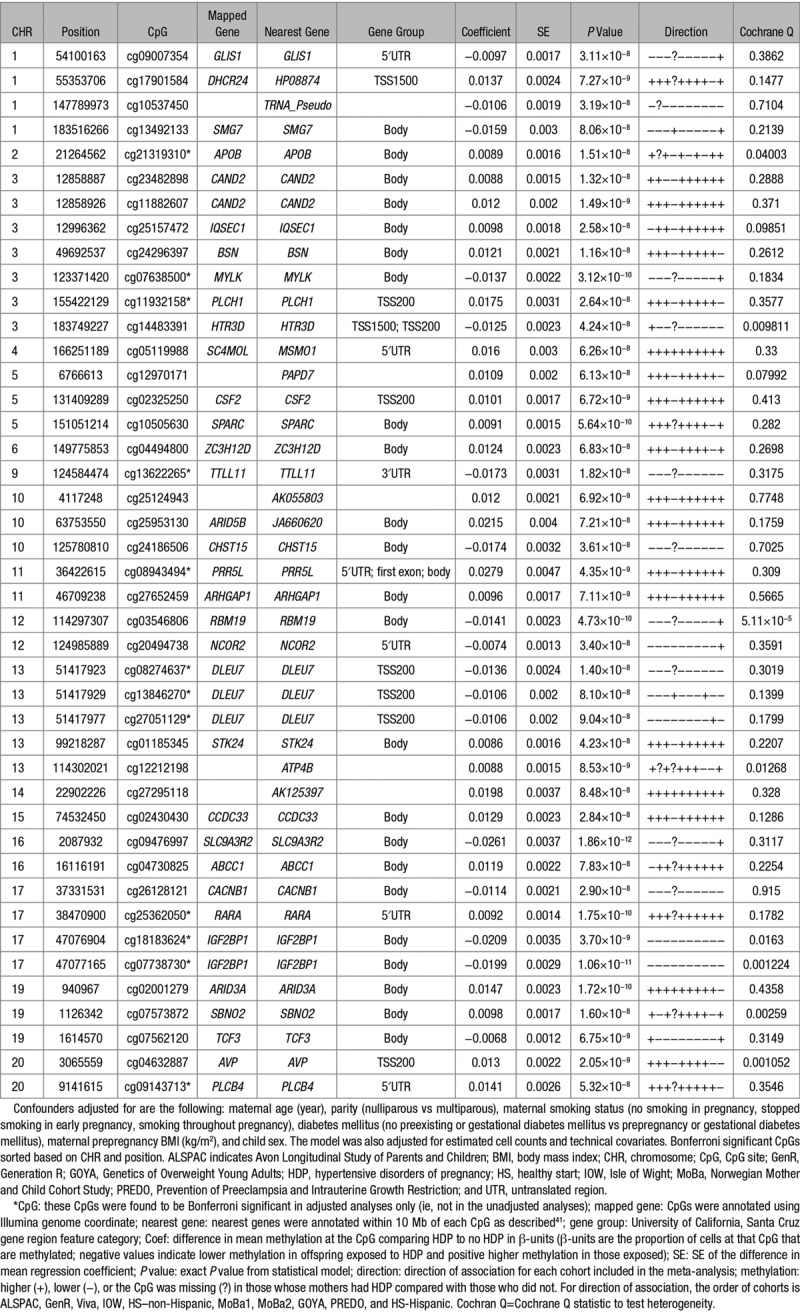
Associations Between HDP and Cord Blood DNA Methylation Levels at CpGs That Surpassed the Bonferroni Significance Threshold (Adjusted for Confounders, Cell-Type Proportions, and Technical Covariates)

**Figure.**
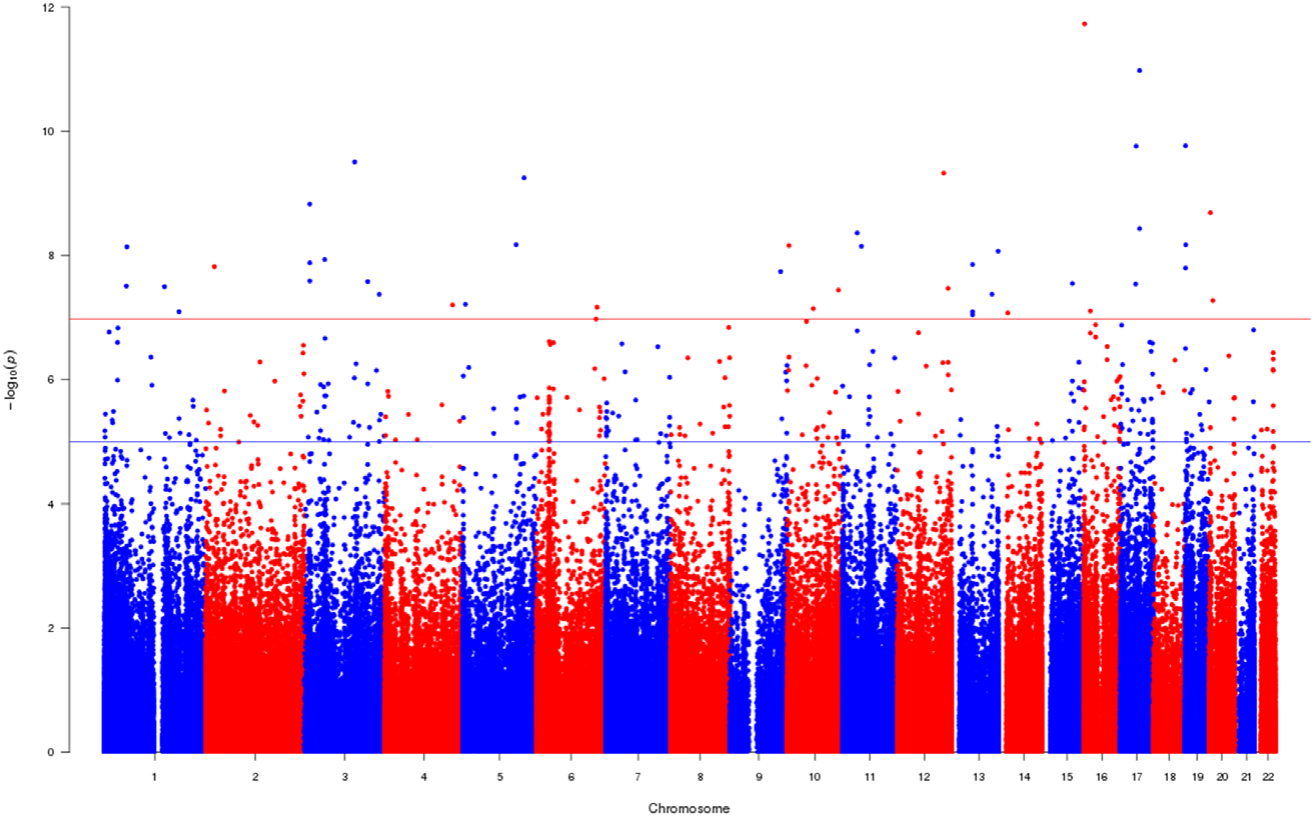
A Manhattan plot indicating the association between hypertensive disorders of pregnancy and cord blood DNA methylation in a meta-analysis. The model was adjusted for confounders; maternal age (years), parity (nulliparous vs multiparous), maternal smoking status (no smoking in pregnancy, stopped smoking in early pregnancy, smoking throughout pregnancy), diabetes mellitus (no preexisting or gestational diabetes mellitus vs prepregnancy or gestational diabetes mellitus), maternal prepregnancy body mass index (kg/m^2^), and child sex. The model was also adjusted for estimated cell counts and technical covariates. The uncorrected −log_10_(*P*) are plotted. A total of 1075 CpG sites (CpGs) reached the false discovery rate threshold (blue line) and 43 CpGs surpassed the Bonferroni threshold (red line).

In the EWAS meta-analysis evaluating the association between preeclampsia and differential DNA methylation in cord blood after adjustment for confounders, estimated cell counts, and technical covariates, we found 26 differentially methylated sites passed the Bonferroni-corrected *P* (*P*<1.05×10^–7^) threshold (Figure S2; Table S4). Preeclampsia was associated with higher methylation levels at 23 (88%) of the CpGs, and across all CpGs, associations were somewhat stronger than HDP associations, with the range of mean difference in methylation being 1.1% to 4.4%. In unadjusted meta-analysis of the association of preeclampsia with cord blood DNA methylation, 117 CpGs reached the Bonferroni *P* (Table S5), including 12 of the 24 identified in the adjusted analyses. When we directly compared the direction and size of association in the 2 EWAS of preeclampsia (unadjusted EWAS versus fully adjusted EWAS), we found moderately strong consistency (R^2^=0.41, slope=0.918±0.002; Figure S3). When we used a 5% false discovery rate method to account for multiple testing (rather than Bonferroni correction), a much larger number of CpGs (n=542) were differentially methylated in relation to preeclampsia in the fully adjusted model (Figure S2; Table S4).

The genomic inflation factor, λ, for all HDP and preeclampsia models is provided in (Tables S6 and S7, respectively). λ-Values for preeclampsia were higher than the meta-analysis results for HDP, likely because of smaller number of preeclampsia cases. Five of the differentially methylated sites for preeclampsia were also identified in the Bonferroni-corrected analyses for HDP. When we directly compared the association direction and sizes of the 2 EWAS (EWAS of HDP and EWAS of preeclampsia), we found modest consistency between the two (R^2^=0.26, slope=1.17±0.003; Figure S4).

There was some statistical evidence of between study heterogeneity for one of the 43 HDP-related CpGs at a stringent Bonferroni-corrected Cochrane Q *P* (*P*<0.001) threshold (Table S2). For the preeclampsia EWAS, there was no evidence of interstudy heterogeneity for all 26 CpGs at Bonferroni-corrected Cochrane Q *P* threshold of 0.002 (Table S4). The results of leave-one-out analysis illustrated that no single cohort had a disproportionally large influence on the meta-analysis results, and most cohorts agreed on the direction of effect at the CpGs that surpassed the Bonferroni significance (Figures S5 and S6). We compared our EWAS results of HDP and preeclampsia to previously published EWAS of early onset of preeclampsia (EOPE) in cord blood of offspring.^17^ Ten of the 43 and 7 of the 26 CpGs that were found in the meta-analysis results of HDP and preeclampsia, respectively, were previously reported in relation to EOPE (Tables S2 and S4). All these CpGs showed the same direction of association in both studies.

Epidemiological studies have suggested that the offspring of mothers with HDP/preeclampsia have a higher risk of long-term hypertension.^6,42^ An EWAS of systolic BP and diastolic BP was conducted among individuals of European, black, and Hispanic/Latino ancestry (n=17 101).^43^ We compared our EWAS results of HDP and preeclampsia to this previously reported EWAS but did not find any overlap. We also compared our EWAS results to those previously reported in cord blood EWAS of birth weight^44^ and gestational age.^45^ Three CpGs related to HDP in our analysis have been previously shown to be differentially methylated in cord blood in relation to birth weight,^44^ with opposite directions of association compared with the direction we found for HDP (ie, where HDP associates with lower methylation levels and higher birth weight associates with higher methylation levels; Table S8; Figure S7A). These findings suggest that the known association of HDP with lower birth weight may be mediated through DNA methylation at these CpGs. Additionally, 19 of the 43 CpGs we identified in our HDP EWAS have been previously shown to be differentially methylated in cord blood in association with gestational age^45^ (Table S9; Figure S7B). Again, the direction of association estimated for HDP was opposite to that estimated for gestational age, suggesting that the association between HDP and lower gestational age might be mediated through DNA methylation.

Pathway analyses were performed using top CpGs identified in the meta-analyses results for enrichment of certain gene ontology terms or biologic pathways. The HDP pathway analysis identified several categories of biologic processes including organ and system development, regulation of cell communication, and cell differentiation. The details are provided in online-only Data Supplement.

### DMR Analysis

DMR analyses were performed to identify associations of HDP and preeclampsia with regions of DNA methylation in cord blood. DMR analysis identified HDP associating with 248 regions (annotated to 203 genes) and preeclampsia associating with 185 regions (annotated to 164 genes) with adjustment for confounders and white cell counts at Bonferroni-corrected *P* (Tables S10 and S11).

In a comparison to EWAS analyses, 30 of the 39 unique genes annotated to HDP-associated CpGs, overlapped with genes identified in DMR analysis of HDP (Table S12). Similarly, 24 genes of the 25 unique genes annotated to preeclampsia-associated CpGs overlapped with the results of DMR analysis of preeclampsia (Table S13).

### Longitudinal Analysis of HDP and Offspring Methylation

Longitudinal mixed-model analyses were undertaken in a subset of the ALSPAC cohort (n=108 HDP cases and 550 controls). As an illustration of the general pattern of observed results, Figure S8 shows longitudinal changes in methylation for the top 4 CpGs reached Bonferroni-corrected *P* threshold in the main adjusted HDP cord blood EWAS meta-analysis (all results are provided in Table S14). There were similar increases in methylation levels between birth and adolescence for most of the 43 CpGs in offspring of mothers who experienced HDP and those who did not. For a small number of CpGs, this age-related change was weaker and less consistent between 7 and 17 than between birth and 7 years. For all but 1 of the 43 CpGs, there was no strong statistical evidence that age-related change differed between offspring of cases and controls, suggesting that differences persisted but that this was because of general age-related change rather than any further long-term effect of exposure to HDP in utero. For the CpG cg08274637 (near *DLEU7* gene), there was evidence that offspring of HDP mothers (compared with those whose mothers who did not experience HDP) had a slightly faster increase in methylation between birth and age 7 (0.27% increased methylation change per year; 95% CI, 0.13%–0.41% methylation change per year; *P*=0.0002).

## Discussion

In this study, we found that HDP (including GH and preeclampsia) and preeclampsia alone are associated with multiple epigenetic methylation marks in cord blood. In our meta-analyses, HDP and preeclampsia were associated with both increased and reduced methylation with generally small mean differences. There were only modest levels of consistency in the magnitude and direction of associations between the preeclampsia and HDP EWAS results, suggesting that overall DNA methylation patterns in offspring cord blood may differ between preeclampsia and GH. However, we acknowledge that for preeclampsia analyses, only 3 independent studies contributed and the overall sample size was small. A previous study examined the association of EOPE with DNA methylation in offspring cord blood using 12 cases of EOPE compared with 8 controls.^17^ There was a small overlap between the results of EWAS of our study and previously reported EWAS of EOPE.^17^ As EOPE is a different outcome to our outcomes and this previous study is smaller than ours, we might not anticipate substantial overlap. A previous study reported the association of systolic BP and diastolic BP with DNA methylation in blood. There was no overlap seen between the results of cord blood EWAS of HDP/preeclampsia exposure from our study and the previous adult blood EWAS and cross-sectional association with BP. Hypertension is a multifactorial disease involving multiple genetical and environmental factors and is mediated by alterations in multiple biologic pathways. The lack of overlap between studies may be related to that prior findings of methylation levels in adult blood may be consequences of elevated BP or lifestyle leading to both change in methylation and high BP, while our findings may reflect developmental impact of prenatal exposure to maternal hypertensive conditions.

The DMR analyses of HDP and preeclampsia identified a region that was annotated to *AVP* gene. Arginine vasopressin is essential for both osmotic and cardiovascular homeostasis and exerts important physiological regulation through 3 receptors, V1a, V1b, and V2.^46^ Results of animal study showed that the V1a receptor has an important role in normal resting arterial BP regulation mainly by regulating circulating blood volume and through baroreflex sensitivity.^46^ Arginine vasopressin has also been previously associated with congestive heart failure.^47^

HDP and a higher maternal BP across pregnancy are associated with small for gestational age, low birth weight, and a shorter gestation.^48–50^ We did not adjust our main analyses for gestational age or birth weight as these could not plausibly influence HDP (and so could not confound the association) but may be on a causal path between HDP and its treatment (including early delivery) and cord blood DNA methylation. In support of this, Mendelian randomization analyses suggest a causal inverse effect of maternal BP on birth weight.^51^ In this study, we found evidence of overlap between results for differential DNA methylation in cord blood related to HDP and CpGs that have been shown to be related to birth weight^44^ or gestational age^45^ that directionally would support potential epigenetic mediation of any effect of HDP on birth weight and gestational age.

Although we postulate that DNA methylation lies on a causal pathway between HDP and birth weight, we cannot discount the possibility that HDP is exerting effects on DNA methylation and birth weight/gestational age or that the associations we have observed are explained by residual confounding. Additional analyses, such as Mendelian randomization, could help to elucidate causal effects,^52^ but we were unable to use Mendelian randomization in this study because of the lack of available maternal genetic instruments specific for HDP. Longitudinal analyses suggested that DNA methylation levels increased with age across most of the HDP-related CpGs but that this was similar in offspring of mothers with and without HDP. These findings suggest that the association of HDP with differential methylation in cord blood remains, but there is no further effect (beyond what is seen at birth) of HDP on differential DNA methylation at these CpGs. These longitudinal analyses were conducted in just 1 study, with no possibility to explore replication, and had limited power. We cannot exclude the possibility that we had larger numbers of studies and participants and more repeat measures of epigenome-wide data, we might have identified an effect of HDP on age-related DNA methylation change. Difference in DNA methylation observed at birth may still influence health at older ages through a transient influence on gene expression.

To assess the underlying biology involved in the associated genomic regions, we performed pathway analyses. These results implicated developmental, embryogenesis, and neurological pathways. These results could provide insight into the pathogenesis of offspring health outcomes related to maternal HDP. The exploration of biologic pathways of the observed differential methylation must also consider the effect sizes detected in this study. Although statistically robust, the changes observed were in the range of 0.6% to 2.6%. The impact of this modest magnitude of differential DNA methylation at a tissue or organ level and hence their influence on health outcomes is currently unknown. Further research on the functional relevance of methylation changes at specific sites is required to further elucidate this.

There are several strengths to our study, including a large sample size, control for variation of DNA methylation levels between blood cells, and the ability to explore change in any cord blood differentially methylated sites as offspring age in 1 study. We were able to control for several potential confounders but as in any epidemiologic study, we cannot be certain that residual confounding does not influence our results and this might be one reason for inflation in our EWAS results. The association between HDP and differential DNA methylation in cord blood may be because of any treatments used for HDP (rather than HDP per se). With moderate or severe hypertension, treatment may include β-blockers, and women with HDP may have their infant delivered early. We did not have information on medications to explore their potential role. The time between birth and processing of cord blood may impact DNA methylation, and it is possible that this may differ between those with and without HDP (eg, cesarean section may be more common in women with HDP and may influence the time from delivery to sample processing); we do not have information on this time interval to adjust for it.

In conclusion, we identified associations between both maternal HDP and preeclampsia and DNA methylation at several loci across the genome in cord blood. Pathway analysis suggested that several of these CpGs are involved in developmental embryogenesis or neurological pathways. We also found overlap between HDP-related differentially methylated CpGs from our study and those found in EWAS of birth weight and gestational age, in directions that are consistent with associations of HDP with these outcomes (eg, higher methylation at CpGs related to HDP being related to lower birth weight and shorter gestational age). However, the biologic and health relevance of the relatively small mean differences in DNA methylation found in our EWAS is unclear, and further research, including the use of methods to distinguish causation from association, is needed to fully understand our findings.

## Perspectives

HDP including GH and preeclampsia is associated with adverse offspring outcomes. Epidemiologic studies have found the associations between HDP and offspring health outcomes including a higher risk of cardiovascular disease. The mechanisms underlying these associations are partly unclear but environmentally responsive; DNA methylation—a type of epigenetic modification—may mediate the relationship between the genetic sequence, environmental factors, and offspring health outcomes. This study identified differences in cord blood methylation pattern with HDP. These findings begin to help us to understand the biologic processes that HDP may influence and possibly mechanisms by which HDP may effect offspring health outcomes.

## Acknowledgments

For all studies, acknowledgments information can be found in the online-only Data Supplement.

## Sources of Funding

For all studies, funding information can be found in the online-only Data Supplement.

## Disclosures

D.A. Lawlor has received support from Roche Diagnostics and Medtronic, as well as from government and charitable funding bodies to support research unrelated to that presented here. T.R. Gaunt receives funding from GlaxoSmithKline and Biogen for unrelated research projects. The other authors report no conflicts.

## Supplementary Material

## Novelty and Significance

### What Is New?

- Epigenome-wide association studies were conducted to identify associations of hypertensive disorders of pregnancy (HDP; including gestational hypertension and preeclampsia) and preeclampsia with DNA methylation at CpG sites and regions in cord blood.
- The study meta-analyzed participant samples (n=5242; 476 with HDP) from 10 independent birth cohorts from the pregnancy and childhood epigenetics consortium. To the best of our knowledge, this is the largest epigenome-wide association study of HDP and preeclampsia.
- We performed novel longitudinal analysis to investigate the change in differentially methylated CpG sites identified in relation to HDP in cord blood from birth to 17 years of age using data from one of the cohorts (n=658; 108 with HDP).

### What Is Relevant?

- This study could identify novel epigenetic mechanisms that mediate effects of HDP on birth weight, gestational length, and future offspring cardiovascular outcomes.
- HDP-associated DNA methylation patterns may act as biomarkers of disease or be modifiable targets for disease treatment in the future.

### Summary

HDP including gestational hypertension and preeclampsia has been associated with shorter gestational age, lower birth weight, and subsequent cardiovascular diseases in women and their offspring. DNA methylation could be a potential mediator of these associations. This study found associations of HDP with differentially methylated CpG sites and regions across the genome in cord blood. It also found some evidence that these CpG sites are related to development, embryogenesis, and neurological outcomes. These findings may be relevant to associations of HDP adverse perinatal and later outcomes.

## References

[1] Gifford RW, August PA, Cunningham G, Green LA, Lindheimer MD, McNellis D, Roberts JM, Sibai BM, Taler SJ (2000). Report of the National High Blood Pressure Education Program Working Group on high blood pressure in pregnancy.. Am J Obstet Gynecol.

[2] Sivakumar S, Bhat BV, Badhe BA (2007). Effect of pregnancy induced hypertension on mothers and their babies.. Indian J Pediatr.

[3] Goldenberg RL, Culhane JF, Iams JD, Romero R (2008). Epidemiology and causes of preterm birth.. Lancet.

[4] Chaim SRP, de Oliveria SMJV, Kimura AF (2008). Pregnancy-induced hypertension and the neonatal outcome.. Acta Paulista de Enfermagem.

[5] Baulon E, Fraser WD, Piedboeuf B, Buekens P, Xiong X (2005). Pregnancy-induced hypertension and infant growth at 28 and 42 days postpartum.. BMC Pregnancy Childbirth.

[6] Fraser A, Nelson SM, Macdonald-Wallis C, Sattar N, Lawlor DA (2013). Hypertensive disorders of pregnancy and cardiometabolic health in adolescent offspring.. Hypertension.

[7] Timpka S, Macdonald-Wallis C, Hughes AD (2016). Hypertensive Disorders of Pregnancy and Offspring Cardiac Structure and Function in Adolescence.. J Am Heart Assoc.

[8] Staley JR, Bradley J, Silverwood RJ (2015). Associations of blood pressure in pregnancy with offspring blood pressure trajectories during childhood and adolescence: findings from a prospective study.. J Am Heart Assoc.

[9] Gluckman PD, Hanson MA, Buklijas T (2010). A conceptual framework for the developmental origins of health and disease.. J Dev Orig Health Dis.

[10] Blair JD, Yuen RK, Lim BK, McFadden DE, von Dadelszen P, Robinson WP (2013). Widespread DNA hypomethylation at gene enhancer regions in placentas associated with early-onset pre-eclampsia.. Mol Hum Reprod.

[11] Anton L, Brown AG, Bartolomei MS, Elovitz MA (2014). Differential methylation of genes associated with cell adhesion in preeclamptic placentas.. PLoS One.

[12] Chelbi ST, Mondon F, Jammes H (2007). Expressional and epigenetic alterations of placental serine protease inhibitors: SERPINA3 is a potential marker of preeclampsia.. Hypertension.

[13] Kulkarni A, Chavan-Gautam P, Mehendale S, Yadav H, Joshi S (2011). Global DNA methylation patterns in placenta and its association with maternal hypertension in pre-eclampsia.. DNA Cell Biol.

[14] Yuen RK, Peñaherrera MS, von Dadelszen P, McFadden DE, Robinson WP (2010). DNA methylation profiling of human placentas reveals promoter hypomethylation of multiple genes in early-onset preeclampsia.. Eur J Hum Genet.

[15] Mousa AA, Archer KJ, Cappello R, Estrada-Gutierrez G, Isaacs CR, Strauss JF, Walsh SW (2012). DNA methylation is altered in maternal blood vessels of women with preeclampsia.. Reprod Sci.

[16] Anderson CM, Ralph JL, Wright ML, Linggi B, Ohm JE (2014). DNA methylation as a biomarker for preeclampsia.. Biol Res Nurs.

[17] Ching T, Ha J, Song MA, Tiirikainen M, Molnar J, Berry MJ, Towner D, Garmire LX (2015). Genome-scale hypomethylation in the cord blood DNAs associated with early onset preeclampsia.. Clin Epigenetics.

[18] He J, Zhang A, Fang M, Fang R, Ge J, Jiang Y, Zhang H, Han C, Ye X, Yu D, Huang H, Liu Y, Dong M (2013). Methylation levels at IGF2 and GNAS DMRs in infants born to preeclamptic pregnancies.. BMC Genomics.

[19] Khatri P, Sirota M, Butte AJ (2012). Ten years of pathway analysis: current approaches and outstanding challenges.. PLoS Comput Biol.

[20] Relton CL, Gaunt T, McArdle W (2015). Data resource profile: Accessible Resource for Integrated Epigenomic Studies (ARIES).. Int J Epidemiol.

[21] Fraser A, Macdonald-Wallis C, Tilling K, Boyd A, Golding J, Davey Smith G, Henderson J, Macleod J, Molloy L, Ness A, Ring S, Nelson SM, Lawlor DA (2013). Cohort profile: the Avon Longitudinal Study of Parents and Children: ALSPAC mothers cohort.. Int J Epidemiol.

[22] Boyd A, Golding J, Macleod J, Lawlor DA, Fraser A, Henderson J, Molloy L, Ness A, Ring S, Davey Smith G (2013). Cohort Profile: the ‘children of the 90s’–the index offspring of the Avon Longitudinal Study of Parents and Children.. Int J Epidemiol.

[23] Kruithof CJ, Kooijman MN, van Duijn CM (2014). The Generation R Study: Biobank update 2015.. Eur J Epidemiol.

[24] Kooijman MN, Kruithof CJ, van Duijn CM (2016). The Generation R Study: design and cohort update 2017.. Eur J Epidemiol.

[25] Nohr EA, Frydenberg M, Henriksen TB, Olsen J (2006). Does low participation in cohort studies induce bias?. Epidemiology.

[26] Paternoster L, Evans DM, Nohr EA (2011). Genome-wide population-based association study of extremely overweight young adults–the GOYA study.. PLoS One.

[27] Starling AP, Brinton JT, Glueck DH, Shapiro AL, Harrod CS, Lynch AM, Siega-Riz AM, Dabelea D (2015). Associations of maternal BMI and gestational weight gain with neonatal adiposity in the Healthy Start study.. Am J Clin Nutr.

[28] Sauder KA, Kaar JL, Starling AP, Ringham BM, Glueck DH, Dabelea D (2017). Predictors of infant body composition at 5 months of age: the healthy start study.. J Pediatr.

[29] Arshad SH, Karmaus W, Zhang H, Holloway JW (2017). Multigenerational cohorts in patients with asthma and allergy.. J Allergy Clin Immunol.

[30] Magnus P, Irgens LM, Haug K, Nystad W, Skjaerven R, Stoltenberg C, MoBa Study Group (2006). Cohort profile: the Norwegian Mother and Child Cohort Study (MoBa).. Int J Epidemiol.

[31] Rønningen KS, Paltiel L, Meltzer HM, Nordhagen R, Lie KK, Hovengen R, Haugen M, Nystad W, Magnus P, Hoppin JA (2006). The biobank of the Norwegian Mother and Child Cohort Study: a resource for the next 100 years.. Eur J Epidemiol.

[32] Girchenko P, Hamalainen E, Kajantie E (2017). Cohort Profile: Prediction and prevention of preeclampsia and intrauterine growth restriction (PREDO) study.. Int J Epidemiol.

[33] Oken E, Kleinman KP, Olsen SF, Rich-Edwards JW, Gillman MW (2004). Associations of seafood and elongated n-3 fatty acid intake with fetal growth and length of gestation: results from a US pregnancy cohort.. Am J Epidemiol.

[34] Willer CJ, Li Y, Abecasis GR (2010). METAL: fast and efficient meta-analysis of genomewide association scans.. Bioinformatics.

[35] Lee CH, Cook S, Lee JS, Han B (2016). Comparison of two meta-analysis methods: inverse-variance-weighted average and weighted sum of Z-scores.. Genomics Inform.

[36] Viechtbauer W (2010). conducting meta-analyses in R with the metafor Package.. J Stat Softw.

[37] Perrier F, Novoloaca A, Ambatipudi S (2018). Identifying and correcting epigenetics measurements for systematic sources of variation.. Clin Epigenetics.

[38] Peters TJ, Buckley MJ, Statham AL, Pidsley R, Samaras K, V Lord R, Clark SJ, Molloy PL (2015). De novo identification of differentially methylated regions in the human genome.. Epigenetics Chromatin.

[39] Laird NM, Ware JH (1982). Random-effects models for longitudinal data.. Biometrics.

[40] Goldstein H (1986). Multilevel mixed linear model analysis using iterative generalized least squares.. Biometrika.

[41] Joubert BR, Felix JF, Yousefi P (2016). DNA methylation in newborns and maternal smoking in pregnancy: genome-wide consortium meta-analysis.. Am J Hum Genet.

[42] Geelhoed JJ, Fraser A, Tilling K, Benfield L, Davey Smith G, Sattar N, Nelson SM, Lawlor DA (2010). Preeclampsia and gestational hypertension are associated with childhood blood pressure independently of family adiposity measures: the Avon Longitudinal Study of Parents and Children.. Circulation.

[43] Richard MA, Huan T, Ligthart S, BIOS Consortium (2017). DNA methylation analysis identifies loci for blood pressure regulation.. Am J Hum Genet.

[44] Agha G, Hajj H, Rifas-Shiman SL, Just AC, Hivert MF, Burris HH, Lin X, Litonjua AA, Oken E, DeMeo DL, Gillman MW, Baccarelli AA (2016). Birth weight-for-gestational age is associated with DNA methylation at birth and in childhood.. Clin Epigenetics.

[45] Bohlin J, Håberg SE, Magnus P, Reese SE, Gjessing HK, Magnus MC, Parr CL, Page CM, London SJ, Nystad W (2016). Prediction of gestational age based on genome-wide differentially methylated regions.. Genome Biol.

[46] Koshimizu TA, Nasa Y, Tanoue A (2006). V1a vasopressin receptors maintain normal blood pressure by regulating circulating blood volume and baroreflex sensitivity.. Proc Natl Acad Sci USA.

[47] Funayama H, Nakamura T, Saito T, Yoshimura A, Saito M, Kawakami M, Ishikawa SE (2004). Urinary excretion of aquaporin-2 water channel exaggerated dependent upon vasopressin in congestive heart failure.. Kidney Int.

[48] Bakker R, Steegers EA, Hofman A, Jaddoe VW (2011). Blood pressure in different gestational trimesters, fetal growth, and the risk of adverse birth outcomes: the generation R study.. Am J Epidemiol.

[49] Steer PJ, Little MP, Kold-Jensen T, Chapple J, Elliott P (2004). Maternal blood pressure in pregnancy, birth weight, and perinatal mortality in first births: prospective study.. BMJ.

[50] Macdonald-Wallis C, Tilling K, Fraser A, Nelson SM, Lawlor DA (2014). Associations of blood pressure change in pregnancy with fetal growth and gestational age at delivery: findings from a prospective cohort.. Hypertension.

[51] Tyrrell J, Richmond RC, Palmer TM, Early Growth Genetics (EGG) Consortium (2016). Genetic evidence for causal relationships between maternal obesity-related traits and birth weight.. JAMA.

[52] Richmond RC, Hemani G, Tilling K, Davey Smith G, Relton CL (2016). Challenges and novel approaches for investigating molecular mediation.. Hum Mol Genet.

