# Supplementary material for: Hypertensive Disorders of Pregnancy and DNA Methylation in Newborns: Findings From the Pregnancy and Childhood Epigenetics Consortium
Source: Hypertension. 2019 Jun 24;74(2):375–83. doi: 10.1161/HYPERTENSIONAHA.119.12634 (PMC6635125; doi:10.1161/HYPERTENSIONAHA.119.12634)
Supplement: Supplementary file 1 [file hyp-74-375-s001.docx]

**Hypertensive disorders of pregnancy and DNA methylation in newborns: Findings from the PACE Consortium**

Supplemental Material

Nabila Kazmi^1,2^*, Gemma C Sharp^1,2,3^, Sarah E Reese^4^ , Florianne O Vehmeijer^5,6,7^, Jari Lahti^8,9^, Christian M Page^10,11^, Weiming Zhang^12^, Sheryl L Rifas-Shiman^13^, Faisal I Rezwan^14^, Andrew J Simpkin^1,15^, Kimberley Burrows^1,2^, Tom G Richardson^1,2^, Diana L Santos Ferreira^1,2^, Abigail Fraser^1,2^, Quaker E Harmon^4^, Shanshan Zhao^4^, Vincent WV Jaddoe^5,6,7^, Darina Czamara^16^, Elisabeth B Binder^16,17^, Maria C Magnus^1,2,18^, Siri E Håberg^18,19^, Wenche Nystad^10^, Ellen A Nohr^20^, Anne P Starling^21^, Katerina J Kechris^12^, Ivana V Yang^21,22,23^, Dawn L DeMeo^24^, Augusto A Litonjua^25^, Andrea Baccarelli^26^, Emily Oken^13^, John W Holloway^14,27^, Wilfried Karmaus^28^, Syed H Arshad^27^, Dana Dabelea^21,29^, Thorkild IA Sørensen^1,30,31^, Hannele Laivuori^32,33,34,35^, Katri Raikkonen^8^, Janine F Felix^5,6,7^, Stephanie J London^4^, Marie-France Hivert^13,36†^, Tom R Gaunt^1,2,37†^, Debbie A Lawlor^1,2,37†^, Caroline L Relton^1,2,37†^

1 MRC Integrative Epidemiology Unit, University of Bristol, Bristol, UK

2 Population Health Sciences, Bristol Medical School, University of Bristol, Bristol, UK

3 School of Oral and Dental Sciences, University of Bristol, Bristol, UK

4 Division of Intramural Research, National Institute of Environmental Health Sciences, National Institutes of Health, Department of Health and Human Services, Research Triangle Park, NC, USA

5 The Generation R Study Group, Erasmus MC, University Medical Center Rotterdam, Rotterdam, the Netherlands

6 Department of Epidemiology, Erasmus MC, University Medical Center Rotterdam, Rotterdam, the Netherlands

7 Department of Pediatrics, Erasmus MC, University Medical Center Rotterdam, Rotterdam, the Netherlands

8 Department of Psychology and Logopedics, Faculty of Medicine, University of Helsinki, Helsinki, Finland

9 Helsinki Collegium of Advanced Studies University of Helsinki, Helsinki, Finland

10 Division of Mental and Physical Health, Norwegian Institute of Public Health, Oslo, Norway

11 Oslo Centre for Biostatistics and Epidemiology, Oslo University Hospital, Oslo, Norway

12 Department of Biostatistics and Informatics, University of Colorado Anschutz Medical Campus, Aurora, CO, USA

13 Department of Population Medicine, Harvard Medical School, Harvard Pilgrim Health Care Institute, Boston, MA, USA

14 Human Development & Health, Faculty of Medicine, University of Southampton, Southampton SO16 6YD, UK

15 Insight Centre for Data Analytics, National University of Ireland, Galway, Ireland

16 Department of Translational Research in Psychiatry, Max-Planck Institute of Psychiatry, Munich, Germany

17 Department of Psychiatry and Behavioral Sciences, Emory University School of Medicine, Atlanta, GA, USA

18 Centre for Fertility and Health, Norwegian Institute of Public Health, Oslo, Norway

19 Centre for Fertility and Health, Norwegian Institute of Public Health, Oslo, Norway

20 Research Unit for Gynaecology and Obstetrics, Department of Clinical Research, University of Southern Denmark, Odense, DK

21 Department of Epidemiology, University of Colorado Anschutz Medical Campus, Aurora, CO, USA

22 Department of Medicine, University of Colorado Anschutz Medical Campus, Aurora, CO, USA

23 Center for Genes, Environment and Health, National Jewish Health, Denver CO

24 Channing Division of Network Medicine, Brigham and Women's Hospital, Harvard Medical School, Boston, MA, USA

25 Division of Pediatric Pulmonary Medicine, University of Rochester Medical Center, Rochester, NY, USA

26 Laboratory of Precision Environmental Biosciences, Columbia University Mailman School of Public Health, New York, NY, USA

27 Clinical & Experimental Sciences, Facultys of Medicine, University of Southampton, Southampton SO16 6YD, UK

28 Division of Epidemiology, Biostatistics, and Environmental Health, School of Public Health, University of Memphis, Memphis, TN 38152, USA

29 Department of Pediatrics, University of Colorado Anschutz Medical Campus, Aurora, CO, USA

30 Novo Nordisk Foundation Center for Basic Metabolic Research, Section on Metabolic Genetics, Faculty of Health and Medical Sciences, University of Copenhagen, Copenhagen, Denmark

31 Department of Public Health, Section on Epidemiology, Faculty of Health and Medical Sciences, University of Copenhagen, Copenhagen, Denmark

32 Medical and Clinical Genetics, University of Helsinki and Helsinki University Hospital, Helsinki, Finland

33 Institute for Molecular Medicine Finland, Helsinki Institute of Life Science, University of Helsinki, Helsinki. Finland

34 Faculty of Medicine and Life Sciences, University of Tampere, Tampere, Finland
35 Department of Obstetrics and Gynecology, Tampere University Hospital, Tampere, Finland

36 Diabetes Unit, Massachusetts General Hospital, Boston, MA, USA

37 NIHR Bristol Biomedical Research Centre, Bristol, UK

† These authors are joint senior authors and contributed equally to this work

* Corresponding author:

Dr Nabila Kazmi

Telephone number: +44 (0) 117 3310106

Email: [nabila.kazmi@bristol.ac.uk](mailto:nabila.kazmi@bristol.ac.uk)

## Supplemental Material and Methods

Definition of HDP

We investigated two hypertensive traits: 1) HDP (women with either GH or PE) and 2) PE alone. Where possible, studies used the International Society for the Study of Hypertension in Pregnancy (ISSHP) criteria for defining HDP^1^. According to these criteria, GH is defined as systolic blood pressure (BP)>139 mm Hg and/or diastolic BP>89 mm Hg on at least two separate occasions, between 20 weeks of gestation and delivery, in women who had not been diagnosed with hypertension before pregnancy and PE is defined as GH, with the addition that there is evidence of proteinuria co-occurring with the high blood pressure (proteinuria is defined as ≥300 mg in a 24hour sample or dip-stick test on a random sample episodes of ≥30 mg/dl, equivalent to ≥1+ on dip stick). Where studies were not able to apply these criteria with certainty, HDP and PE were defined on the basis of a clinical diagnosis appearing in their maternal medical records or the women reporting that they had been diagnosed with HDP (More detail of how HDP/PE was defined for each study is provided in the sections about individual cohorts). Controls (in both analyses of HDP and PE) were women who did not have any evidence of HDP. Women diagnosed with hypertension before pregnancy were excluded from all analyses.

Covariates

We adjusted for the following characteristics which we *a priori* selected on the basis that it was plausible they would influence HDP and cord-blood DNA methylation (and as such fit the criteria of a potential confounder^2^): cell-type, maternal age (years), parity (categorized into nulliparous versus multiparous), maternal smoking status (the preferred categorization was into three groups: no smoking in pregnancy, stopped smoking in early pregnancy, smoking throughout pregnancy, but a binary categorization of any versus no smoking was also used in cohorts unable to adjust for the three level category), diabetes (no pre-existing or gestational diabetes versus pre-pregnancy or gestational diabetes), maternal pre-pregnancy BMI (kg/m^2^). Child sex (male/female) was also adjusted for in all models. Whilst this could not plausibly be influence by HDP adjusting for sex differences in DNA methylation could improve the statistical efficiency of our analyses. Each cohort also adjusted for technical covariates using methods suitable for each respective cohort (details for each cohort is written in following sections).

All cohorts ran ethnicities separately to reduce heterogeneity. We did not adjust for genetic principal components (PCs) because not all cohorts had genome-wide genotype data and cohorts with genotype data had it only for a subset of subjects with methylation data. One of the previous PACE papers perform a sensitivity analysis in ALSPAC and GenR adjusting for ancestry PCs that did not reduce the inflation (lambda)^3^.

The birthweight and gestational age and the differences in these two traits by HDP (GH + PE) and PE alone for all cohorts is provided in (Supplemental Table S15 and Table S16). The results showed small differences between controls and cases of HDP and PE, as expected based on consequences of these conditions on fetal growth and treatment (induction of delivery).

Methylation measurements and quality control

DNA methylation assays were performed in cohort-specific laboratories. DNA was extracted from cord-blood and underwent bisulfite conversion (details on methods used by each cohort are given in following sections). Samples were processed using Illumina Infinium® HumanMethylation450 (HM450) BeadChip assays. Sample pre-processing was followed by cohort-specific quality control and normalisation of methylation data. Each cohort excluded samples and probes that failed their own quality control procedures, as detailed in the following sections. For the meta-analysis, we excluded control probes (n=65) and probes mapping to the sex chromosomes (n=11,648).

After running meta-analysis, we additionally excluded non-CpG probes, polymorphic probes (defined as SNP-overlapping probes, probes with a SNP at the target CpG sites (CpGs), or probes with a SNP at the base next to the target CpG) with minor-allele frequency (MAF) ≥ 5%; based on UCSC common SNPs track for dbSNP build 137 from our FDR significant probes. We further excluded the remaining probes that are considered cross-hybridizing^4^. We applied this stringent CpG filtering because polymorphic and cross-hybridizing probes can interfere with accurate detection of methylation levels.

Estimation of cell-type proportions

All cohorts estimated cell-type proportions from Bakulski cord-blood reference^5^ using the Houseman algorithm^6^. This method estimates the relative proportions of seven white blood cell subtypes: B cells, CD4 T cells, CD8 T cells, granulocytes, monocytes, natural killer (NK) cells and nucleated red blood cells. The Bakulski cord-blood reference has been validated and showed high correlations between the estimated and measured composition for nRBCs (r=0.92, R^2^=0.85), lymphocytes (r=0.77, R^2^=0.58), and granulocytes (r=0.72, R^2^=0.52), and a moderate correlation for monocytes (r=0.51, R^2^=0.25) as well as relatively low root mean square errors from the residuals ranging from 1.4 to 5.4%^7^. The differences in predicted seven cell-types between HDP/PE controls and cases for all participating cohorts are provided in (Supplemental Table S17 and Table S18).

CpG annotation

The gene name for each CpG was annotated using the Illumina Infinium® HM450 BeadChip annotation file^8^. We also annotated all CpGs for nearest genes within 10Mb of each CpG, as previously described^9^. We include this expanded gene annotation in our tables.

Cohort-specific statistical analyses

Each cohort conducted independent EWAS using a common, pre-specified analysis plan. Each cohort ran robust linear regression models (RLM) in R^10^ to evaluate the association between HDP or PE and cord-blood DNA methylation. RLM was chosen because of its robustness to potential heteroskedasticity and/or influential outliers in the methylation data. We have found in the PACE consortium that extreme outliers, likely caused by rare SNPs or technical errors can drive results considerably. These seem to happen at different probes in different cohorts, and it is not always the same sample that is the outlier. We have explored various different methods to remove outliers but found the Tukey method^11^ was the best over multiple cohorts. This method does not depend on the distributional assumptions of the data and is increasingly used as a solution to this problem^12-14^. To remove extreme outliers, we trimmed the methylation set using: (25th percentile -3*IQR) and (75th percentile+3*IQR), where IQR = interquartile range. This means that for each probe, values outside this range was set to NA in the methylation set. A sample R code for this procedure is now provided as an example. The number of samples remained after trimming for each probe for all cohorts is provided in the (Supplemental Tables S19-28). Each cohort ran EWAS models with HDP (or PE) as the exposure and normalised untransformed methylation beta values as the outcome, with adjustment for confounders and estimated cell counts.

Cohorts used different approaches to normalize the data, but previous work from this consortium suggest that different normalizing approaches might reduce statistical efficiency but are unlikely to importantly bias results^9^.

Example R code to remove outliers:

rowIQR <- rowIQRs(meth, na.rm = T)

row2575 <- rowQuantiles(meth, probs = c(0.25, 0.75), na.rm = T)

maskL <- meth < row2575[,1] - 3 * rowIQR

maskU <- meth > row2575[,2] + 3 * rowIQR

initial_NAs<-rowSums(is.na(meth))

meth[maskL] <- NA

removed_lower <- rowSums(is.na(meth))-initial_NAs

meth[maskU] <- NA

removed_upper <- rowSums(is.na(meth))-removed_lower-initial_NAs

N_for_probe<-rowSums(!is.na(meth))

Pathway analyses

Pathway analyses were carried out using the goseq R package^15^ to test top CpGs for enrichment of certain gene ontology terms or biological pathways and evaluate the potential functionality of genes mapping to differentially methylated CpGs.

The CpGs were linked to genes using the 450k BeadChip annotation file. The CpGs associated with HDP and PE based on FDR q-value<0.05 were flagged differentially expressed features. Duplicate gene entries and CpGs lacking an annotated gene symbol were filtered out. A total of 18,614 unique gene identifiers were used the analysis for both HDP and PE.

The HDP pathway analysis included FDR significant CpGs and identified 178 GO categories among 19,788 possible categories using goseq^15^. The identified GO categories were enriched with FDR q-value<0.05 (Supplemental Table S29). Although the analysis did not find any GO category using only Bonferroni significant sites. The analysis identified several categories of biological processes including organ and system development, regulation of cell communication, cell differentiation, digestive tract, digestive system and muscle tissue development and regulation of cellular process. The PE pathway analysis did not identify any GO categories that were enriched with FDR q-value<0.05. The top 15 enriched categories identified based on uncorrected P-values are provided in (Supplemental Table S30). These GO categories include striated muscle cell differentiation, muscle structure development, positive regulation of biological process, TRAIL production and regulation of TRAIL production.

Longitudinal model in ALSPAC

Longitudinal methylation data were extracted for each of the Bonferroni significant CpGs. A multilevel model including a random intercept (to allow for between-subject variability in methylation) and a linear regression spline term (to allow for linear change between two adjacent measures (e.g. birth and age 7) but differences in the magnitude of this over time between age periods (e.g. linear change between birth and age 7 could differ to that between age 7 and 17) was fitted to each of these CpGs separately. For example, for CpGs found on comparing HDP and normotensive mothers:

$${meth}_{ij}= \beta_{0}+ \mu_{0i}+ \beta_{1}{HDP}_{i}+ \beta_{2}{age}_{ij}+ {\beta_{3}\left( {age}_{ij}-7 \right)}_{+}+ \beta_{4}{HDP}_{i}{age}_{ij}+ {\beta_{5}{HDP}_{i}\left( {age}_{ij}-7 \right)}_{+}+confounders+ \varepsilon_{ij}$$

$$\varepsilon_{ij} \sim N(0,\sigma_{\varepsilon}^{2})$$

$$\mu_{0i} \sim N(0, \sigma_{\mu}^{2})$$

where 𝑖 = 1, . . .658 indexes the children in ARIES, 𝑗 = 1,2,3 indexes the measurement occasion and $\left( {age}_{ij}-7 \right)_{+}$ is equal to ${age}_{ij}-7$ when ${age}_{ij}-7$ is greater than 0, or equal to 0 if it is not, i.e. this cannot take a negative value. This term is used to construct a linear spline at age 7. The exact technical meaning of each beta coefficient in the above equation is provided in the box below. Once the model is fitted, we can calculate the change in methylation from 0-7 for children of non-HDP mothers (𝛽_2_), HDP mothers (𝛽_2_ + 𝛽_4_) and the change from 7-17 for children of non-HDP mothers (𝛽_2_ + 𝛽_3_) and HDP mothers (𝛽_2_ + 𝛽_3_ + 𝛽_4_ + 𝛽_5_). To test whether there is a difference in methylation change between 7 and 17, for example, we test whether 𝛽_4_ + 𝛽_5_ is different from zero (the null). This can be done by comparing (𝛽_4_ + 𝛽_5_)/se(𝛽_4_ + 𝛽_5_) to the standard normal distribution, where se is the standard error.

For each CpG, we used the above described multilevel model, adjusting for confounders (maternal age, parity, maternal smoking status, gestational diabetes, maternal pre-pregnancy BMI, child sex and six cell counts (i.e. CD4 T cells, CD8 T cells, NK cells, B cells, monocytes and granulocytes estimated using the Houseman algorithm^6^) at each of birth, age 7 and age 17. In these analyses we adjusted for cell-type using the adult blood reference^16^ and Houseman algorithm. While the adult reference panel may not be optimal for our cord-blood samples, we used it here to keep estimates consistent from birth to age 7 and 17.

Cohorts description

ALSPAC

The Avon Longitudinal Study of Parents and Children (ALSPAC) is a large, prospective cohort study based in the South West of England. 14,541 pregnant women residents in Avon, UK with expected dates of delivery 1st April 1991 to 31st December 1992 were recruited and detailed information has been collected on these women and their offspring at regular intervals^17-19^. The study website contains details of all the data that is available through a fully searchable data dictionary (<http://www.bris.ac.uk/alspac/researchers/data-access/data-dictionary/>).

Written informed consent has been obtained for all ALSPAC participants. Ethical approval for the study was obtained from the ALSPAC Ethics and Law Committee and the Local Research Ethics Committees.

### ALSPAC hypertensive disorders of pregnancy

Data on all repeat measurements of blood pressure and proteinuria (obtained by dip-stick) that were measured across pregnancy were abstracted from the mother’s antenatal medical records by research midwives. A median 14 (IQR: 11 to 16) blood pressure and 12 (IQR: 10 to 14) proteinuria measures, together with the gestational age of measurement and maternal self-report of existing hypertension were used to categories women according to the International Society for the Study of Hypertension in Pregnancy (ISSHP) criteria for defining hypertensive disorders of pregnancy (HDP). Gestational hypertension (GH) was defined as systolic blood pressure>139 mmHg OR diastolic blood pressure>89 mmHG on at least 2 occasions after 20 weeks of gestation in women who had not previously been diagnosed with hypertension. Pre-eclampsia (PE) was defined in the same way as GH but in addition with proteinuria (≥30 mg/dl which is equivalent to ≥1+ on dip stick) occurring at the same time as the episodes of raised blood pressure.

### ALSPAC covariates

Maternal age at delivery was derived from her date of birth and the date of delivery. It was included as a continuous variable. Parity (categorised for this study as nulliparous or multiparous), maternal smoking (categorised for this study as never smoking during pregnancy or any smoking during pregnancy) and history of existing diabetes were assessed during pregnancy via self-completed questionnaires. Gestational diabetes was extracted from antenatal medical records and women were categorised as either no diabetes or either existing or gestational diabetes. Maternal pre-pregnancy weight and height were self-reported in the pregnancy questionnaire, with strong correlation between self-report for weight and weight measured at the first antenatal clinic (Pearson correlation=0.96). BMI was calculated as self-reported weight (kg)/height (m2) and included in the analysis as a continuous variable. Child sex was obtained from midwife or hospital records. Ten surrogate variables for unknown sources of variation in high-throughput experiments were generated and the ones not associated with the exposure at P-value<0.05 (1,3 and 5-9) were included in models to adjust for technical batches^20^.

### ALSPAC methylation measurements

Cord-blood samples were collected according to standard procedures. The DNA methylation wet-lab and pre-processing analyses were performed at the University of Bristol as part of the ARIES project. Following extraction, DNA was bisulphite-converted using the Zymo EZ DNA MethylationTM kit (Zymo, Irvine, CA). Following conversion, genome-wide methylation status of over 485,000 CpGs was measured using the Infinium HM450 BeadChip according to the standard protocol. The arrays were scanned using an Illumina iScan and initial quality review was assessed using GenomeStudio (version 2011.1). Samples from all time points in ARIES were distributed across slides using a semi-random approach (sampling criteria were in place to ensure that all time points were represented on each array) to minimise the possibility of confounding by technical covariates. In addition, during the data generation process, a wide range of batch variables was recorded in a purpose-built laboratory information management system (LIMS). The main batch variable was found to be the bisulphite conversion (BCD) plate number. Samples were converted in batches of 48 samples and each batch identified by a plate number. The LIMS also reported quality control (QC) metrics from the standard control probes on the 450K BeadChip for each sample. Samples failing QC (average probe p value >= 0.01) were repeated and if unsuccessful excluded from further analysis. As an additional QC step genotype probes were compared with SNP-chip data from the same individual to identify and remove any sample mismatches. For individuals with no genome-wide SNP data, samples were flagged if there was a sex-mismatch based on X-chromosome methylation.

Data were pre-processed in R (version 3.0.1) using subset quantile normalization approach described by Touleimat & Tost to reduce the non-biological differences between probes^21^. Probes with a single nucleotide polymorphism and sites on sex chromosomes were excluded to reduce complexity due to sex-specific differences and X-chromosome inactivation by DNA methylation in females. Finally, we also excluded probes showing a detection P-value>0.05 for >5% samples. These exclusions left 471193 probes for the analysis.

Generation R

The Generation R Study is a population-based prospective cohort study from fetal life onwards in Rotterdam, the Netherlands, which has been described in detail elsewhere^22,23^. The study protocol was approved by the Medical Ethics Committee of the Erasmus Medical Center, Rotterdam. Written informed consent was obtained for all participants. All children were born between April 2002 and January 2006 and form a largely prenatally enrolled birth cohort that is currently being followed until young adulthood. A total of 9,778 mothers were included, most during pregnancy (response rate at birth 61%).

### Generation R hypertensive disorders of pregnancy

Information about GH was abstracted from the medical records by a trained medical abstractor and it was derived according to the ISSHP criteria (see details above provided in the ALSPAC cohort description).

### Generation R covariates

Information on maternal age, parity, and smoking was collected using questionnaires during pregnancy. Maternal smoking was defined as never smoking during pregnancy or any smoking during pregnancy. Maternal body mass index was measured in early pregnancy. This measure was strongly correlated with self-reported pre-pregnancy body mass index (Pearson correlation coefficient 0.96). Child sex was obtained from midwife or hospital registries. Maternal gestational diabetes was not included as a covariate in any of the models, due to convergence issues because of the low number of cases. Plate number was used to correct for technical effects. The smallest plate was removed from the analyses.

### Generation R methylation measurements

500 ng DNA per sample extracted (using the salting-out method) from 979 cord-blood samples underwent bisulfite conversion using the EZ-96 DNA Methylation kit (Shallow) (Zymo Research Corporation, Irvine, USA). Samples were plated onto 96-well plates in no specific order. Samples were processed with the Illumina Infinium HumanMethylation450 BeadChip (Illumina Inc., San Diego, USA).

Quality control of analyzed samples was performed using standardized criteria. Samples were excluded in case of low sample call rate (<99%, 6 samples excluded), colour balance >3 (no samples excluded), low staining efficiency (no samples excluded), poor extension efficiency (no samples excluded), poor hybridization performance (no samples excluded), low stripping efficiency after extension (no samples excluded) and poor bisulfite conversion (1 sample removed). In addition, 2 samples were excluded because of a gender mismatch and 1 sample was excluded because of a retracted informed consent, leaving a total of 969 Generation R samples in the analysis.

Probes with a single nucleotide polymorphism in the single base extension site with a frequency of > 1% in the GoNLv4 reference panel^24^ were excluded, as were probes with non-optimal binding (non-mapping or mapping multiple times to either the normal or the bisulphite-converted genome), resulting in the exclusion of 49,564 probes, leaving a total of 436,013 probes in the analysis.

We ran DASES normalization using a pipeline adapted from that developed by Touleimat and Tost^21^. DASES normalization includes background adjustment, between-array normalization applied to type I and type II probes separately, and dye bias correction applied to type I and type II probes separately and is based on the DASEN method described by Pidsley et al, but adds the dye bias correction, which is not included in DASEN^25^.

GOYA

The GOYA study was based on The Danish National Birth Cohort (DNBC). From 1996–2002, 91,387 women with a total of 100,419 pregnancies were recruited to the DNBC in early pregnancy by their GP, and approximately 60% of those invited chose to participate. Detailed descriptions of the study methods and the recruitment were published elsewhere^26,27^. Briefly, the main data collection consisted of two telephone interviews during pregnancy at <16 and <30 weeks of gestation and two postnatal telephone interviews when the child was <6 and 18 months old. Also, the woman provided two blood samples during pregnancy and a blood sample of the child taken from the umbilical cord at birth. When entering the DNBC, all women provided written informed consent that their data and biological material could be used in scientific studies of health in women and children. The main cohort study was approved by all the regional scientific ethics committees in Denmark, by the central scientific ethics committee for whole Denmark and by the Danish Data Protection Board.

### GOYA hypertensive disorders of pregnancy

HDP included both chronic and GH and was identified by ICD-10 codes I10 through I15, and O10, O11, and O13 in The National Patient Register.

### GOYA covariates

Data on covariates were collected via a telephone interview at around 16 weeks gestation. Maternal age was derived from the mother’s report of her own date of birth. Parity was categorized for this study as nulliparous or parous. Maternal smoking in pregnancy was defined never smoking during pregnancy or any smoking during pregnancy. Maternal pre-pregnancy BMI was calculated from self-reported height and weight. Diabetes included both pre-gestational and gestational diabetes and was identified by the ICD-10 codes E10 before or in pregnancy and O24 in pregnancy. Because we suspected some under-reporting of gestational diabetes in the register, we also relied on self-reported information on diabetes from the telephone interviews. This increased the frequency of gestational diabetes from 0.9% to 1.2% in the overall cohort.

### GOYA methylation measurements

Cord-blood was collected according to standard procedures, spun and frozen at -80˚C. DNA methylation analysis and data pre-processing were performed at the University of Bristol. Following extraction, DNA was bisulfite converted using the Zymo EZ DNA MethylationTM kit (Zymo, Irvine, CA). Following conversion, the genome-wide methylation status of over 485,000 CpGs was measured using the Illumina Infinium® HumanMethylation450k BeadChip assay according to the standard protocol. The arrays were scanned using an Illumina iScan and initial quality review was assessed using GenomeStudio (version 2011.1). Further quality checks include CpG detection p-value test which removed the samples whose average detection p-value is > 0.01. We also used several study specific variables and created multidimensional scaling (MDS) plots to identify any potential outliers but all samples were within the thresholds. The level of methylation is expressed as a “Beta” value (β-value), ranging from 0 (no cytosine methylation) to 1 (complete cytosine methylation). Raw beta values were normalised using the Functional Normalisation method of minfi package^28^. We excluded SNPs, probes with a detection p-value > 0.05 in more than 5% samples and features on sex chromosomes. After excluding these features 473389 and 473382 probes were left for analyses. Technical effects were corrected by SVA.

Healthy Start

The Healthy Start study is an ongoing, pre-birth cohort study that recruited pregnant women from outpatient obstetrics clinics at the University of Colorado Hospital in 2009-2014. Eligible women were 16 years or older, having completed 24 or fewer gestational weeks of a singleton pregnancy, with no history of stillbirth or extremely preterm birth, and no chronic diseases including diabetes, cancer, or asthma treated with steroid medications. The study recruited an ethnically diverse population of 1,410 pregnant women (~50% of those eligible). The Colorado Multiple Institutional Review Board approved procedures. Informed consent was obtained from pregnant women prior to participation.

### Healthy Start hypertensive disorders of pregnancy

Information on clinical diagnosis of GH was abstracted from maternal medical records by trained study personnel shortly after delivery. The obstetricians at the University of Colorado hospital system followed the current ACOG guidelines for diagnosis at the time of the patient visit (2009-2014).

### Healthy Start covariates

Maternal age, race/ethnicity, parity, and history of diabetes were obtained via self-report at enrolment. Maternal history of type 1 or type 2 diabetes at enrolment was an exclusion criterion for the Healthy Start study. Women with a prior history of gestational diabetes were not excluded. Maternal pre-pregnancy body mass index was obtained from the medical record or, if unavailable, via self-report at enrolment. Maternal smoking status was assessed via questionnaire and coded as ever or never smoked. Information on child sex was obtained from the medical record shortly after delivery.

### Healthy Start methylation measurements

Umbilical cord-blood was collected at delivery. DNA was extracted from stored buffy coats using the QIAamp kit (Qiagen). DNA purity was checked using 260/280 and 260/230 readings from the Nanodrop spectrophotometer while DNA quantification was performed using the Qubit fluorimeter. Bisulfite conversion, labelling and hybridisation were performed in the University of Colorado Denver Genomics Core. Methylation analysis was conducted using the Illumina Infinium HumanMethylation450 BeadChip. We removed 587 probes with high detection P value (> 0.05). We removed 664 probes with a bead count < 3 in at least 5% of samples. We compared the predicted sex to the reported sex, and samples with inconsistent sex were removed. The preprocessQuantile function in minfi was used for normalization. We used ComBat to adjust for technical covariates ^29^.

Isle of Wight (IOW) 3^rd^ Generation

A whole population birth cohort was established on the Isle of Wight, UK, in 1989 to prospectively study the natural history of allergic diseases from birth onwards. Both the Isle of Wight and the study population are 99% Caucasian. Ethics approvals were obtained from the Isle of Wight Local Research Ethics Committee (now named the National Research Ethics Service, NRES Committee South Central – Southampton B) at recruitment and for the 1, 2, 4, 10 and 18 years follow-up. Of the 1,536 children born between January 1, 1989, and February 28, 1990, written informed consent was obtained from parents to enrol 1,456 newborns. Children were followed up at the ages of 1 (n=1,167), 2 (n=1,174), 4 (n=1,218), 10 (n=1,373), and 18 years (n=1,313). From January 2012 to May 2016, we further recruited 367 1989-1990 cohort participants and 124 newborns of these participants^30^. These 124 mother-child pairs are included in the current study.

### Isle of Wight hypertensive disorders of pregnancy

Information about GH was abstracted from the Hospital Maternity notes and it was derived according to the ISSHP criteria (see details above provided in the ALSPAC cohort description).

### Isle of Wight covariates

Data on covariates were collected via questionnaires collected at recruitment during the 1st trimester of pregnancy, including maternal age, smoking status during pregnancy and parity. Information on maternal age was collected by questionnaire at enrolment and it is included in the analysis as a continuous variable. Maternal smoking during pregnancy was assessed by questionnaires at 20 and 28 weeks of pregnancy, and 3 months after birth. Maternal pre-pregnancy BMI was calculated from maternal height and first trimester weight. Indicators of different batches that DNA methylation data were generated were included as a covariate to adjust for technical covariates. No information on maternal diabetes was available. Sex of the child was obtained from the hospital admin system (patient centres).

### Isle of Wight methylation measurements

Epigenome-wide DNA methylation was measured using DNA extracted from cord-blood, and 1000 ng DNA per sample underwent bisulfite conversion using the EZ-96 DNA Methylation kit (Shallow) (Zymo Research Corporation, Irvine, USA). Samples were plated onto 96-well plates in no specific order. Samples were processed with the Illumina Infinium HumanMethylation450 BeadChip (Illumina Inc., San Diego, USA).

Quality control of analyzed samples was performed using standardized criteria. All samples are over 99.8% probes detected. Samples were deleted if more than 75% CpGs of that sample with detection p value larger than or equal to 10^-5^, CpGs were deleted if 10% of the samples with detection p value larger than or equal to 0.01. CpGs with missing values caused by insufficient copies of a probe binding to the sample DNA were excluded from the study.

Data were pre-processed using IMA^31^ package in R including quantile normalization to reduce inter-array variation and type I and type II probe peak correction^32^. Beta-values were calculated for all CpGs. Additionally, we only took common CpGs between our 6 batches of samples and run ComBat on them to remove technical effect.

MoBa

Participants in the current analysis represent two subsets of mother-offspring pairs from the national Norwegian Mother and Child Cohort Study (MoBa). Each subset is referred as MoBa1 and MoBa2. MoBa1 and MoBa2 study populations were part of a larger study within MoBa that was designed to evaluate the association between maternal plasma folate during pregnancy and childhood asthma status at 3 years of age^33^. The association between maternal smoking during pregnancy and differential DNA methylation in 1,062 MoBa1 newborns has been previously reported^34^. Subsequently DNA methylation was measured in an additional 685 newborns with maternal plasma folate measurements following separate quality control and preprocessing (MoBa2). These analyses include the children who had cord-blood DNA methylation measurements, information on HDP, and covariate data (n=1020 from MoBa1 and n=644 from MoBa2), and each dataset was analyzed independently. The year of birth for participants in these MoBa participants ranged from 2000-2009. Both studies were approved by the Regional Committee for Ethics in Medical Research and the Norwegian Data Inspectorate, and written informed consent was provided by all mothers participating. In addition, MoBa1 and MoBa2 were approved by the Institutional Review Board of the National Institute of Environmental Health Sciences, USA.

### MoBa hypertensive disorders of pregnancy

The diagnosis of GH and PE was extracted from the Medical Birth Registry of Norway (MBRN) notification form^35^.

### MoBa covariates

For both MoBa1 and MoBa2 datasets, information on maternal weight and height (used to calculated BMI), maternal age, child sex, and parity were collected via questionnaires completed by the mother or from birth registry records. Maternal BMI and age were included as continuous variables. Child’s sex was included as a dichotomous variable (1 = male; 2 = female). Parity was categorized into two groups; 0 = nulliparous and 1 = multiparous (at least one previous birth). Maternal smoking during pregnancy (yes vs. no) was assessed by maternal questionnaire and plasma cotinine, a biomarker of tobacco smoking, measured by liquid chromatography - tandem mass spectrometry at approximately 18 weeks gestation. If a mother reported not smoking but had plasma cotinine ≥ 56.8 nmol/L she was categorized as a smoker. If a mother reported that she stopped in pregnancy and had cotinine values < 56.8 nmol/L she was classified as a non-smoker. Maternal diabetes was extracted from MBRN notification form. It was recorded in the form by marking tick boxes for “Pre-existing diabetes type 1”, “Pre-existing diabetes type 2” and “Gestational diabetes”. For this study we categorised women as no diabetes or any of pre-existing type 1, pre-existing type 2 or gestational diabetes.

### MoBa methylation measurements

The protocols for DNA methylation measurements and quality control for the MoBa1 and MoBa2 participants were same. Umbilical cord-blood samples were collected and frozen at birth at -80°C. All biological material was obtained from the Biobank of the MoBa study^36^. Bisulfite conversion was performed using the EZ-96 DNA Methylation kit (Zymo Research Corporation, Irvine, CA) and DNA methylation was measured at 485,577 CpGs in cord-blood using Illumina’s Infinium HumanMethylation450 BeadChip. Raw intensity (.idat) files were handled in R using the minfi package to calculate the methylation level at each CpG as the beta-value (β=intensity of the methylated allele (M)/(intensity of the unmethylated allele (U) + intensity of the methylated allele (M) + 100)) and the data was exported for quality control and processing.

Probe and sample-specific quality control was performed in the MoBa1 and MoBa2 datasets separately. Similar protocols were applied to MoBa1 and Moba2, as follows: Control probes (n=65) and probes on X (n=11,230) and Y (n=416) chromosomes were excluded in both datasets. Remaining CpGs missing >10% of methylation data were also removed (n=20 in MoBa1, none in MoBa2). Samples indicated by Illumina to have failed or have an average detection p value across all probes < 0.05 (n=49 MoBa1, n=35 MoBa2) and samples with gender mismatch (n=13 MoBa1, n=8 MoBa2) were also removed. For MoBa1 and MoBa2, we accounted for the two different probe designs by applying the intra-array normalization strategy Beta Mixture Quantile dilation (BMIQ)^37^. The Empirical Bayes method via ComBat was applied separately in each dataset for technical correction. Methylation features were filtered from (i) cross-reactive probes, (ii) probes mapping to sex chromosomes and (iii) probes overlapping with a known single nucleotide polymorphism (SNP) with an allele frequency of at least 5% in the overall population (all ethnic groups), resulting in the exclusion of 36,231 probes. Data quality was further assessed using box plots for the distribution of methylated and unmethylated signals, and multidimensional scaling plots and unsupervised clustering were used to check for sample outliers. After background correction and color-bias adjustment, type I and type II probe distributions were aligned using the intra-array beta-mixture quantile normalization (BMIQ) from the watermelon package. Beta-values were calculated for all CpGs. Technical effects were corrected using COMBAT.

PREDO

Data were from the Prediction and Prevention of Preeclampsia and Intrauterine Growth Restriction (PREDO) Study, which is a longitudinal multicenter pregnancy cohort study of Finnish women and their singleton children born alive between 2006-2010^38^. We recruited 1079 pregnant women, of whom 969 had one or more and 110 had none of the known risk factors for PE and intrauterine growth restriction. The recruitment took place in arrival order when these women attended the first ultrasound screening at 12+0-13+6 weeks+days of gestation in one of the ten hospital maternity clinics participating in the study. The cohort profile^38^ contains details of the study design and inclusion criteria. The study protocol was approved by the Ethical Committees of the Helsinki and Uusimaa Hospital District and by the participating hospitals. A written informed consent was obtained from all women. The study has been registered as ClinicalTrials.gov identifier ISRCTN14030412.

### PREDO hypertensive disorders of pregnancy

Information about HDP was extracted from the Finnish Medical Birth Register. GH and PE were diagnosed according to the ISSHP criteria (see details above provided in the ALSPAC cohort description). Each individual diagnosis was further verified by a clinical jury based on a review of individual patient case records.

##

### PREDO covariates

Data on all covariates were derived from the Finnish Medical Birth Register. Gestational diabetes diagnosis was further verified by a clinical jury. Covariates included maternal age at delivery (included as a continuous variable), parity (categorised for this study as nulliparous or multiparous), maternal smoking (categorised for this study as never smoked or ever smoked), gestational diabetes (categorised for this study as no diabetes versus pre-pregnancy or gestational diabetes), and maternal pre-pregnancy weight and height verified by a measurement conducted at first antenatal clinical visit taking place on average at 8^th^ gestational week and child’s sex. BMI was calculated as from weight (kg)/height (m2) (included in the analysis as a continuous variable).

### PREDO methylation measurements

Cord-blood samples were ran on Illumina 450K Methylation arrays. To limit technical effects, we randomized all samples over the 96-well plates, based on gender and maternal risk factors for PE. Samples were placed on 96-well plates. Bisulfite conversion was performed using the EZ-96 DNA methylation kit (Zymo research Corporation, Irvine, USA). Then we used the Infinium HumanMethylation450 BeadChip (Illumina Inc., San Diego, USA) to measure the methylation level as a beta value ranging from 0 (no methylation) to 1 (complete methylation). The quality control pipeline was set up using the R-package minfi*.* Three IDs were excluded as they were outliers in the median intensities. Furthermore, twenty IDs showed discordance between phenotypic sex and estimated sex and were excluded. Nine IDs were contaminated with maternal DNA and were also removed.

Methylation beta-values were normalized using the Functional normalistaion method. We excluded any probes on chromosome X or Y, probes containing SNPs and cross-hybridizing probes according to Chen et al.^4^ and Price et al^39^. Furthermore, any CpGs with a detection p-value > 0.01 in at least 25% of the samples were excluded.

After normalization two batches, i.e. slide and well, were significantly associated and were removed iteratively using the ComBat method.

Project Viva

Project Viva is a population-based prospective pre-birth cohort of mothers and their children in Eastern Massachusetts, USA, which has been described in detail elsewhere^40^. The study has been approved by the Institutional Review Board of Harvard Pilgrim Health Care and written consent was obtained from participating women. Women were enrolled from 1999 to 2002 and enrolment included a total of 2,128 live births. Follow up of the children through early adolescence is ongoing.

### Project Viva hypertensive disorders of pregnancy

Information about GH was abstracted from the medical records and it was derived according to the ISSHP criteria (see details above provided in the ALSPAC cohort description).

### Project Viva covariates

Maternal age, parity, pregnancy smoking status and child sex were collected using questionnaires. We calculated maternal pre-pregnancy BMI using self-reported pre-pregnancy weight and height. Maternal age and pre-pregnancy BMI were used as continuous variables.

### Project Viva methylation measurements

DNA samples extracted from cord-blood were platted using a two-stage stratified randomization scheme to ensure balance of cohort characteristics across sample plates and chips. Samples were bisulfite converted using the EZ-96 DNA Methylation kit (Zymo Research Corporation, Irvine, USA) and measured using. the Illumina Infinium HumanMethylation450 BeadChip (Illumina Inc., San Diego, USA) run at Illumina FastTrack Microarray Services following standard manufacturer’s protocols (San Diego, CA). We excluded samples with genotype and/or sex mismatch. Sample pre-processing included the exclusion of allosomal probes, non-CpG probes, and failing probes. A total of 470,411 CpGs remained for analyses.).

We adjusted for dye-bias and performed a background correction using the normal-exponential out-of-band (noob) function in R. We used the BMIQ (beta-mixture quantile) function in the wateRmelon package of R to scale the distribution of the type-2 probes present in the array. Finally, we used ComBat in the processed data to adjust for technical covariates. We included maternal age, pre-pregnancy BMI, and smoking during pregnancy and child sex and parity as covariates in the ComBat adjustment. We limited the analysis to white children.

**Supplemental Acknowledgements**

**ALSPAC:** We are extremely grateful to all the families who took part in this study, the midwives for their help in recruiting them, and the whole ALSPAC team, which includes interviewers, computer and laboratory technicians, clerical workers, research scientists, volunteers, managers, receptionists, and nurses.

**Generation R:** The Generation R Study is conducted by the Erasmus Medical Center in close collaboration with the School of Law and Faculty of Social Sciences of the Erasmus University Rotterdam, the Municipal Health Service Rotterdam area, Rotterdam, the Rotterdam Homecare Foundation, Rotterdam and the Stichting Trombosedienst & Artsenlaboratorium Rijnmond (STAR-MDC), Rotterdam. We gratefully acknowledge the contribution of children and parents, general practitioners, hospitals, midwives and pharmacies in Rotterdam. The study protocol was approved by the Medical Ethical Committee of the Erasmus Medical Centre, Rotterdam. Written informed consent was obtained for all participants. The generation and management of the Illumina 450K methylation array data (EWAS data) for the Generation R Study was executed by the Human Genotyping Facility of the Genetic Laboratory of the Department of Internal Medicine, Erasmus MC, the Netherlands. We thank Mr. Michael Verbiest, Ms. Mila Jhamai, Ms. Sarah Hunter, Mr. Marijn Verkerk and Dr. Lisette Stolk for their help in creating the EWAS database.

**GOYA:** GOYA (Genomics of Obesity in Young Adults) was sampled as a case-cohort study within the Danish National Birth Cohort. The Danish National Birth Cohort was established with a significant grant from the Danish National Research Foundation. Additional support was obtained from the Danish Regional Committees, the Pharmacy Foundation, the Egmont Foundation, the March of Dimes Birth Defects Foundation, the Health Foundation and other minor grants. The DNBC Biobank has been supported by the Novo Nordisk Foundation and the Lundbeck Foundation.

**IOW 3rd generation cohort:** IOW cohort acknowledges the great help from the nurses at the David Hide Asthma and Allergy Research Centre led by Professor Hasan Arshad. We greatly appreciate the participating families in the third-generation study. IOW Researchers are grateful to Stephen Potter for data processing.

**MoBa 1 and 2:** We are grateful to all the participating families in Norway who take part in the ongoing MoBa cohort study. We also acknowledge Dr. Frank Day of Integrative Bioinformatics at the NIEHS, Dr. Jianping Jin of Westat, and Elin Alsaker of the National Institute of Public Health, Bergen, Norway for their expert computational and data management assistance.

**PREDO**: The PREDO study would not have been possible without the dedicated contribution of the PREDO Study group members: P Villa, E Hämäläinen, E Kajantie. We also thank the PREDO cohort mothers, fathers and children for their enthusiastic participation.

**Project Viva:** We are indebted to the Project Viva mothers, children and families for their ongoing participation.

**Funding Support**

**ALSPAC**: The UK Medical Research Council and the Wellcome Trust (Grant ref: 102215/2/13/2) and the University of Bristol provide core support for ALSPAC. The Accessible Resource for Integrated Epigenomics Studies (ARIES) which generated large scale methylation data was funded by the UK Biotechnology and Biological Sciences Research Council (BB/I025751/1 and BB/I025263/1). Additional support for the work presented here comes from the Wellcome Trust (WT088806), the United States National Institute of Diabetes and Digestive and Kidney Diseases (R01DK10324), the European Research Council (DevelopObese; 669545) and European Union’s Horizon 2020 research and innovation programme (733206, LIFECYCLE). NK, GCS, AJS, KB, TGR, DLSF, AF, TRG, DAL and CLR work in a Unit that received funds from the University of Bristol and UK Medical Research Council (MC_UU_00011/4, MC_UU_00011/15, MC_UU_00011/6-7); this Unit also contributed funds to some of the cord-blood DNA methylation assays in ALSPAC. AF, TGR, DAL, and CLR also supported by the UK National Institute of Health Research Biomedical Research Centre at University Hospitals Bristol NHS Foundation Trust and the University of Bristol, and DAL is an NIHR Senior Investigator (NF-SI-0611-10196). The funders had no role in study design, data collection and analysis, decision to publish, or preparation of the manuscript.

**Generation R:** The Generation R Study is made possible by financial support from the Erasmus Medical Center, Rotterdam, the Erasmus University Rotterdam and the Netherlands Organization for Health Research and Development. The EWAS data was funded by a grant to VWJ from the Netherlands Genomics Initiative (NGI)/Netherlands Organisation for Scientific Research (NWO) Netherlands Consortium for Healthy Aging (NCHA; project nr. 050-060-810), by funds from the Genetic Laboratory of the Department of Internal Medicine, Erasmus MC, and by a grant from the National Institute of Child and Human Development (R01HD068437). V.W.J. received a grant from the Netherlands Organization for Health Research and Development (VIDI 016.136.361) and a Consolidator Grant from the European Research Council (ERC-2014-CoG-648916). J.F.F. has received funding from the European Union’s Horizon 2020 research and innovation programme under grant agreement No 633595 (DynaHEALTH). This project received funding from the European Union’s Horizon 2020 research and innovation programme (733206, LIFECYCLE).

**GOYA:** Genotyping for the GOYA Study was funded by the Wellcome Trust (Grant ref: 084762MA). Generation of DNA methylation data was funded by the MRC Integrative Epidemiology Unit, which is supported by the Medical Research Council (MC_UU_12013/1-9) and the University of Bristol.

**Healthy Start:** The Healthy Start study was supported by grants from the National Institute of Diabetes and Digestive and Kidney Diseases (R01DK076648) and the National Institute of Environmental Health Sciences (R01ES022934).

**IOW 3rd generation cohort:** The IOW third generation cohort was funded by NIAID/NIH R01AI091905. This study was supported in part by NIAID/NIH R01AI091905 and R01AI121226.

**MoBa 1 and 2:** This research was supported in part by the Intramural Research Program of the NIH, National Institute of Environmental Health Sciences (Z01-ES-49019) and the Norwegian Research Council/BIOBANK (grant no 221097). The Norwegian Mother and Child Cohort Study is supported by the Norwegian Ministry of Health and the Ministry of Education and Research, NIH/NIEHS (contract no. N01-ES-75558), NIH/NINDS (grant no.1 UO1 NS 047537-01) and the Norwegian Research Council/FUGE (grant no. 151918/S10).

**PREDO**: The PREDO study was supported by the Academy of Finland, EraNet Neuron, EVO (a special state subsidy for health science research), University of Helsinki Research Funds, the Signe and Ane Gyllenberg foundation, the Emil Aaltonen Foundation, the Finnish Medical Foundation, the Jane and Aatos Erkko Foundation, the Novo Nordisk Foundation, the Päivikki and Sakari Sohlberg Foundation, the Sigrid Juselius Foundation, Juho Vainio Foundation, and Hope&Optimism Initiative.

**Project Viva:** This study was supported by the National Institutes of Health (R01 HD034568, R01 NR013945 and R01 HL 111108).

Authors’ contributions

NK, DAL, CR and MFH conceived and designed the study. First authors for each cohort conducted the cohort-specific analyses (NK, SER, FOV, JL, CMP, WZ, SLRS and FIR). NK carried out additional analyses for their cohorts. NK and KB meta-analysed the results. NK wrote the manuscript with initial input on early drafts by DAL, CR and TGR and further input to later drafts from all authors. Correspondence and material requests should be addressed to NK ([nabila.kazmi@bristol.ac.uk](mailto:nabila.kazmi@bristol.ac.uk)).

**References**

1. Tranquilli AL, Dekker G, Magee L, et al. The classification, diagnosis and management of the hypertensive disorders of pregnancy: A revised statement from the ISSHP. *Pregnancy hypertension.* 2014;4(2):97-104.

2. Hernan MA, Hernandez-Diaz S, Werler MM, Mitchell AA. Causal knowledge as a prerequisite for confounding evaluation: an application to birth defects epidemiology. *American journal of epidemiology.* 2002;155(2):176-184.

3. Sharp GC, Salas LA, Monnereau C, et al. Maternal BMI at the start of pregnancy and offspring epigenome-wide DNA methylation: findings from the pregnancy and childhood epigenetics (PACE) consortium. *Human molecular genetics.* 2017;26(20):4067-4085.

4. Chen YA, Lemire M, Choufani S, et al. Discovery of cross-reactive probes and polymorphic CpGs in the Illumina Infinium HumanMethylation450 microarray. *Epigenetics.* 2013;8(2):203-209.

5. Bakulski KM, Feinberg JI, Andrews SV, et al. DNA methylation of cord blood cell types: Applications for mixed cell birth studies. *Epigenetics.* 2016;11(5):354-362.

6. Houseman EA, Accomando WP, Koestler DC, et al. DNA methylation arrays as surrogate measures of cell mixture distribution. *BMC bioinformatics.* 2012;13:86.

7. Cardenas A, Allard C, Doyon M, et al. Validation of a DNA methylation reference panel for the estimation of nucleated cells types in cord blood. *Epigenetics.* 2016;11(11):773-779.

8. Infinium HumanMethylation450K v1.2 Product Files. 2014.

9. Joubert BR, Felix JF, Yousefi P, et al. DNA Methylation in Newborns and Maternal Smoking in Pregnancy: Genome-wide Consortium Meta-analysis. *American journal of human genetics.* 2016;98(4):680-696.

10. Team RC. R: A language and environment for statistical computing (Vienna, Austria: R Foundation for Statistical Computing). 2013.

11. Tukey JW. Exploratory Data Analysis 1977.

12. Sharp GC, Arathimos R, Reese SE, et al. Maternal alcohol consumption and offspring DNA methylation: findings from six general population-based birth cohorts. *Epigenomics.* 2018;10(1):27-42.

13. Sharp GC, Ho K, Davies A, et al. Distinct DNA methylation profiles in subtypes of orofacial cleft. *Clinical epigenetics.* 2017;9:63.

14. Dai L, Mehta A, Mordukhovich I, et al. Differential DNA methylation and PM2.5 species in a 450K epigenome-wide association study. *Epigenetics.* 2017;12(2):139-148.

15. Young MD, Wakefield MJ, Smyth GK, Oshlack A. Gene ontology analysis for RNA-seq: accounting for selection bias. *Genome biology.* 2010;11(2):R14.

16. Reinius LE, Acevedo N, Joerink M, et al. Differential DNA methylation in purified human blood cells: implications for cell lineage and studies on disease susceptibility. *PloS one.* 2012;7(7):e41361.

17. Boyd A, Golding J, Macleod J, et al. Cohort Profile: the 'children of the 90s'--the index offspring of the Avon Longitudinal Study of Parents and Children. *Int J Epidemiol.* 2013;42(1):111-127.

18. Fraser A, Macdonald-Wallis C, Tilling K, et al. Cohort Profile: the Avon Longitudinal Study of Parents and Children: ALSPAC mothers cohort. *International journal of epidemiology.* 2013;42(1):97-110.

19. Relton CL, Gaunt T, McArdle W, et al. Data Resource Profile: Accessible Resource for Integrated Epigenomic Studies (ARIES). *International journal of epidemiology.* 2015;44(4):1181-1190.

20. Leek JT, Ewans WJ, Parker HS, Fertig EJ, Jaffe AE, Storey JD. sva: surrogate variable analysis. *R package version 3140.* 2015.

21. Touleimat N, Tost J. Complete pipeline for Infinium((R)) Human Methylation 450K BeadChip data processing using subset quantile normalization for accurate DNA methylation estimation. *Epigenomics.* 2012;4(3):325-341.

22. Kruithof CJ, Kooijman MN, van Duijn CM, et al. The Generation R Study: Biobank update 2015. *European journal of epidemiology.* 2014;29(12):911-927.

23. Kooijman MN, Kruithof CJ, van Duijn CM, et al. The Generation R Study: design and cohort update 2017. *European journal of epidemiology.* 2016;31(12):1243-1264.

24. Whole-genome sequence variation, population structure and demographic history of the Dutch population. *Nature genetics.* 2014;46(8):818-825.

25. Pidsley R, CC YW, Volta M, Lunnon K, Mill J, Schalkwyk LC. A data-driven approach to preprocessing Illumina 450K methylation array data. *BMC genomics.* 2013;14:293.

26. Nohr EA, Frydenberg M, Henriksen TB, Olsen J. Does low participation in cohort studies induce bias? *Epidemiology (Cambridge, Mass).* 2006;17(4):413-418.

27. Olsen J, Melbye M, Olsen SF, et al. The Danish National Birth Cohort--its background, structure and aim. *Scandinavian journal of public health.* 2001;29(4):300-307.

28. Aryee MJ, Jaffe AE, Corrada-Bravo H, et al. Minfi: a flexible and comprehensive Bioconductor package for the analysis of Infinium DNA methylation microarrays. *Bioinformatics (Oxford, England).* 2014;30(10):1363-1369.

29. Johnson WE, Li C, Rabinovic A. Adjusting batch effects in microarray expression data using empirical Bayes methods. *Biostatistics (Oxford, England).* 2007;8(1):118-127.

30. Arshad SH, Karmaus W, Zhang H, Holloway JW. Multigenerational cohorts in patients with asthma and allergy. *The Journal of allergy and clinical immunology.* 2017;139(2):415-421.

31. Wang D, Yan L, Hu Q, et al. IMA: an R package for high-throughput analysis of Illumina's 450K Infinium methylation data. *Bioinformatics (Oxford, England).* 2012;28(5):729-730.

32. Dedeurwaerder S, Defrance M, Calonne E, Denis H, Sotiriou C, Fuks F. Evaluation of the Infinium Methylation 450K technology. *Epigenomics.* 2011;3(6):771-784.

33. Haberg SE, London SJ, Nafstad P, et al. Maternal folate levels in pregnancy and asthma in children at age 3 years. *The Journal of allergy and clinical immunology.* 2011;127(1):262-264, 264.e261.

34. Joubert BR, Haberg SE, Nilsen RM, et al. 450K epigenome-wide scan identifies differential DNA methylation in newborns related to maternal smoking during pregnancy. *Environmental health perspectives.* 2012;120(10):1425-1431.

35. Klungsoyr K, Harmon QE, Skard LB, et al. Validity of pre-eclampsia registration in the medical birth registry of norway for women participating in the norwegian mother and child cohort study, 1999-2010. *Paediatric and perinatal epidemiology.* 2014;28(5):362-371.

36. Magnus P, Irgens LM, Haug K, Nystad W, Skjaerven R, Stoltenberg C. Cohort profile: the Norwegian Mother and Child Cohort Study (MoBa). *Int J Epidemiol.* 2006;35(5):1146-1150.

37. Teschendorff AE, Marabita F, Lechner M, et al. A beta-mixture quantile normalization method for correcting probe design bias in Illumina Infinium 450 k DNA methylation data. *Bioinformatics (Oxford, England).* 2013;29(2):189-196.

38. Girchenko P, Hamalainen E, Kajantie E, et al. Prediction and Prevention of Preeclampsia and Intrauterine Growth Restriction (PREDO) study. *International journal of epidemiology.* 2016.

39. Price ME, Cotton AM, Lam LL, et al. Additional annotation enhances potential for biologically-relevant analysis of the Illumina Infinium HumanMethylation450 BeadChip array. *Epigenetics Chromatin.* 2013;6(1):4.

40. Oken E, Baccarelli AA, Gold DR, et al. Cohort profile: project viva. *International journal of epidemiology.* 2015;44(1):37-48.

**Figure S1.** Consistency between DNA methylation in fully adjusted model and unadjusted model of HDP.


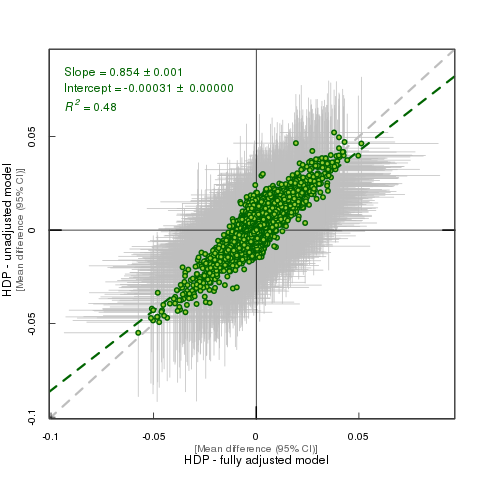


Correlation between DNA methylation association patterns in fully adjusted model and unadjusted model of HDP (green dashed line). DNA methylation associations are shown for fully adjusted and unadjusted models with 473864 common CpGs in offspring white blood cells at birth from meta-analyses of EWAS. Each green dot represents a CpG and the positions of the dots are determined by difference in mean DNA methylation in offspring of mothers with HDP versus normotensive mothers in fully adjusted model (x-axis) and difference in mean DNA methylation in offspring of mothers with HDP versus normotensive mothers in unadjusted model (y-axis). The horizontal grey lines on each dot denote the confidence intervals (CI) for fully adjusted model of HDP and the vertical grey lines indicate the CI for unadjusted model of HDP estimates. A linear fit of the overall correspondence summarises the similarity in magnitude and direction between fully adjusted model of HDP and unadjusted model of HDP associations (green dashed line). A slope of 1 with an intercept of 0 (dashed grey line), with all green dots sitting on that line (R^2^=1), would indicate that fully adjusted model of HDP and unadjusted model of HDP estimates had the same magnitude and direction. R^2^=goodness of linear fit and as such is a measure of the consistency between fully adjusted model of HDP and unadjusted model of HDP associations.

**Figure S2.** A Manhattan plot indicating the association between PE and cord-blood DNA methylation in a meta-analysis.


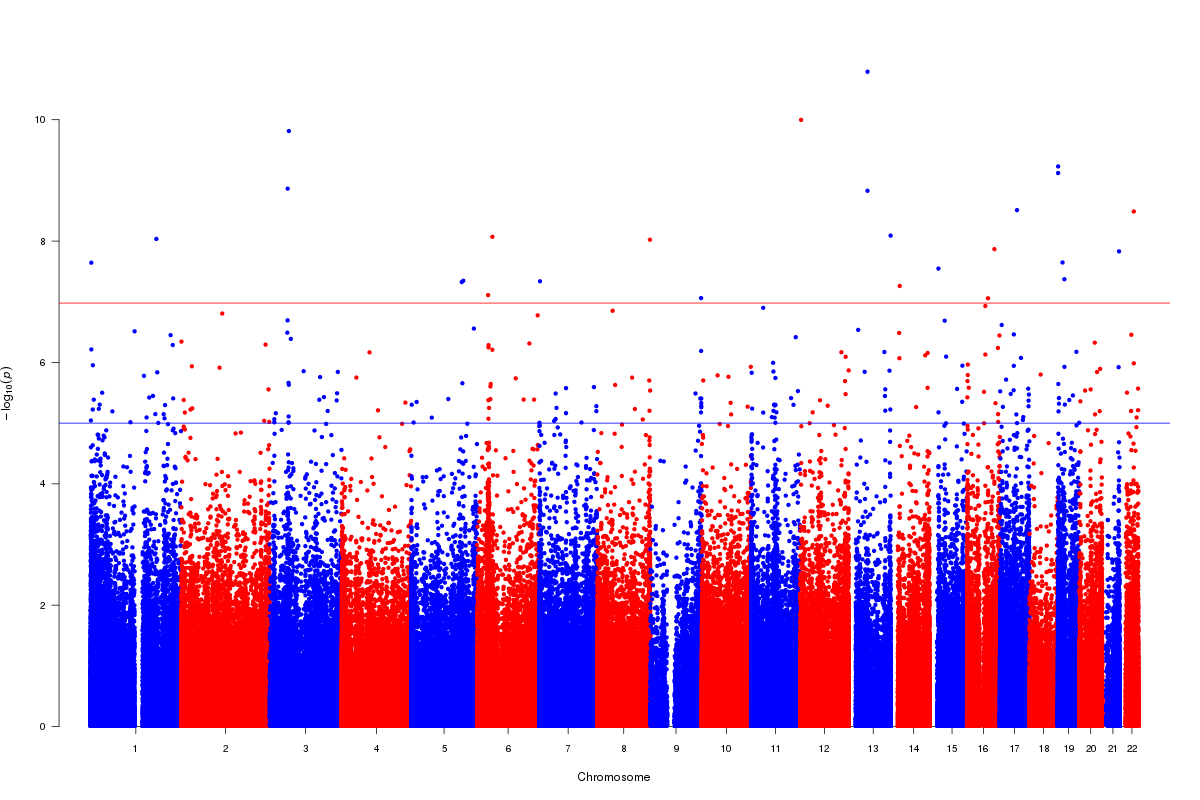


The model was adjusted for confounders; maternal age (years), parity (nulliparous versus multiparous), maternal smoking status (no smoking in pregnancy, stopped smoking in early pregnancy, smoking throughout pregnancy), diabetes (no pre-existing or gestational diabetes versus pre-pregnancy or gestational diabetes), maternal pre-pregnancy BMI (kg/m^2^) and child sex. The model was also adjusted for estimated cell counts and technical covariates. The uncorrected –log_10_(P-values) are plotted. A total of 542 CpGs reached the FDR threshold (blue line) and 26 CpGs surpassed the Bonferroni threshold (red line).

**Figure S3.** Consistency between DNA methylation in fully adjusted model and unadjusted model of PE.


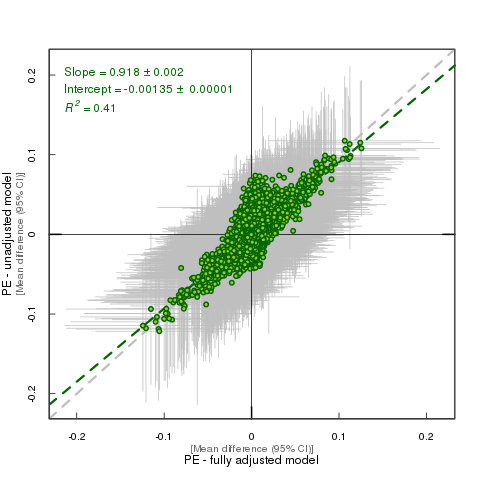


Correlation between DNA methylation association patterns in fully adjusted model and unadjusted model of PE (green dashed line). DNA methylation associations are shown for fully adjusted and unadjusted models with 473864 common CpGs in offspring white blood cells at birth from meta-analyses of EWAS. Each green dot represents a CpG and the positions of the dots are determined by difference in mean DNA methylation in offspring of mothers with PE versus normotensive mothers in fully adjusted model (x-axis) and difference in mean DNA methylation in offspring of mothers with PE versus normotensive mothers in unadjusted model (y-axis). The horizontal grey lines on each dot denote the confidence intervals (CI) for fully adjusted model of PE and the vertical grey lines indicate the CI for unadjusted model of PE estimates. A linear fit of the overall correspondence summarises the similarity in magnitude and direction between fully adjusted model of PE and unadjusted model of PE associations (green dashed line). A slope of 1 with an intercept of 0 (dashed grey line), with all green dots sitting on that line (R^2^=1), would indicate that fully adjusted model of PE and unadjusted model of PE estimates had the same magnitude and direction. R^2^=goodness of linear fit and as such is a measure of the consistency between fully adjusted model of PE and unadjusted model of PE associations.

**Figure S4.** Consistency between DNA methylation in offspring of HDP and PE mothers.


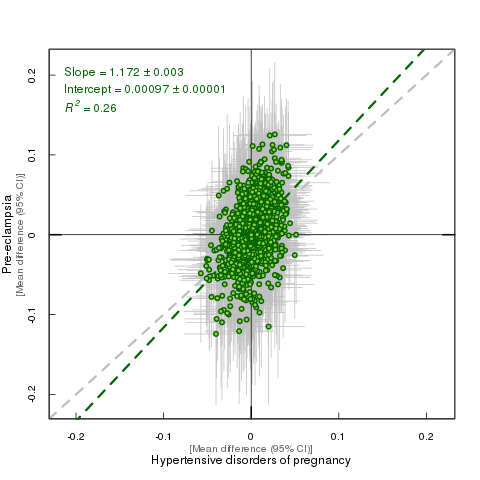


Correlation between DNA methylation association patterns in offspring of HDP and PE mothers (green dashed line). DNA methylation associations are shown for maternal HDP and PE with 473864 common CpGs in offspring white blood cells at birth from meta-analyses of EWAS. Each green dot represents a CpG and the positions of the dots are determined by difference in mean DNA methylation in offspring between mothers with HDP versus normotensive mothers (x-axis) and difference in mean DNA methylation in offspring between mothers with PE versus normotensive mothers (y-axis). The horizontal grey lines on each dot denote the confidence intervals (CI) for HDP and the vertical grey lines indicate the CI for PE estimates. A linear fit of the overall correspondence summarises the similarity in magnitude and direction between HDP and PE associations (green dashed line). A slope of 1 with an intercept of 0 (dashed grey line), with all green dots sitting on that line (R^2^=1), would indicate that HDP and PE estimates had the same magnitude and direction. R^2^=goodness of linear fit and as such is a measure of the consistency between HDP and PE associations.

**Figure S5.** The results of leave-one-out analysis to illustrate that no single cohort had a disproportionally large influence on the meta-analysis results of HDP (using regression coefficients) at the CpGs that surpassed the Bonferroni significance.

**Figure S6.** The results of leave-one-out analysis to illustrate that no single cohort had a disproportionally large influence on the meta-analysis results of PE (using regression coefficients) using regression coefficients at the CpGs that surpassed the Bonferroni significance.

| **Figure S7**. Comparison of our HDP EWAS results to previously reported EWAS of birthweight (A) and EWAS of gestational age (B).  **A)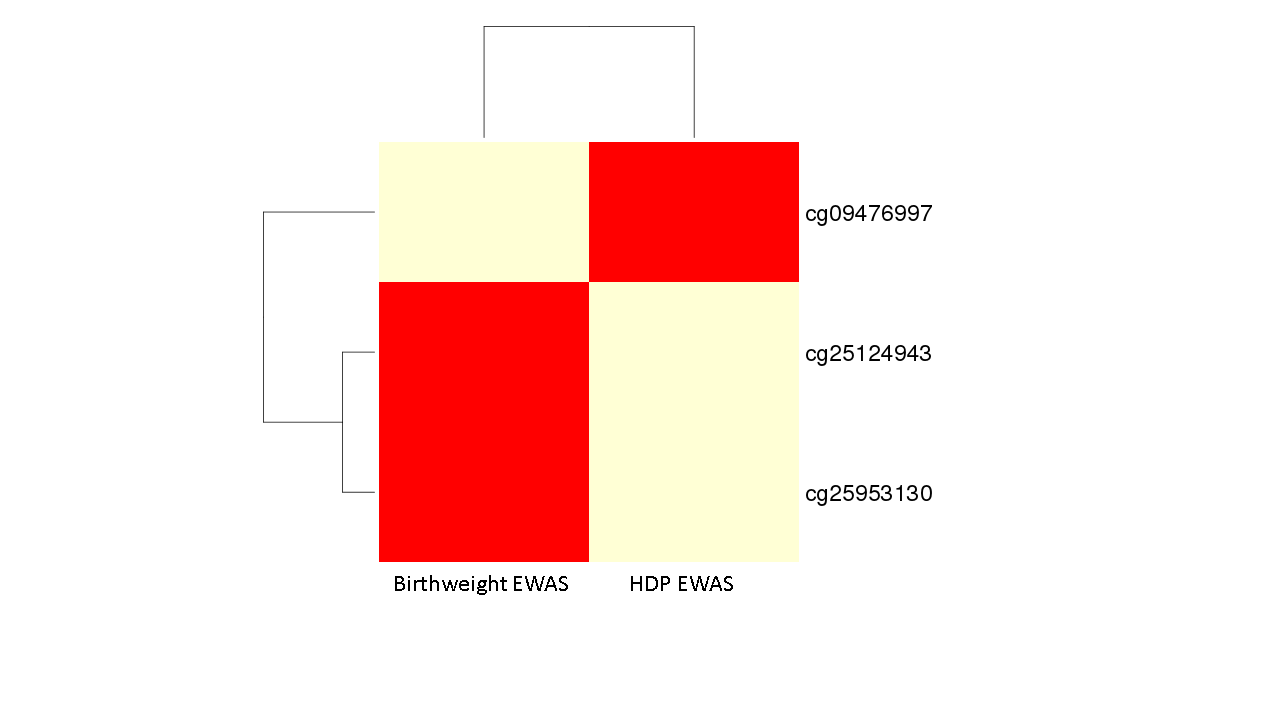** |
| --- |
| **B)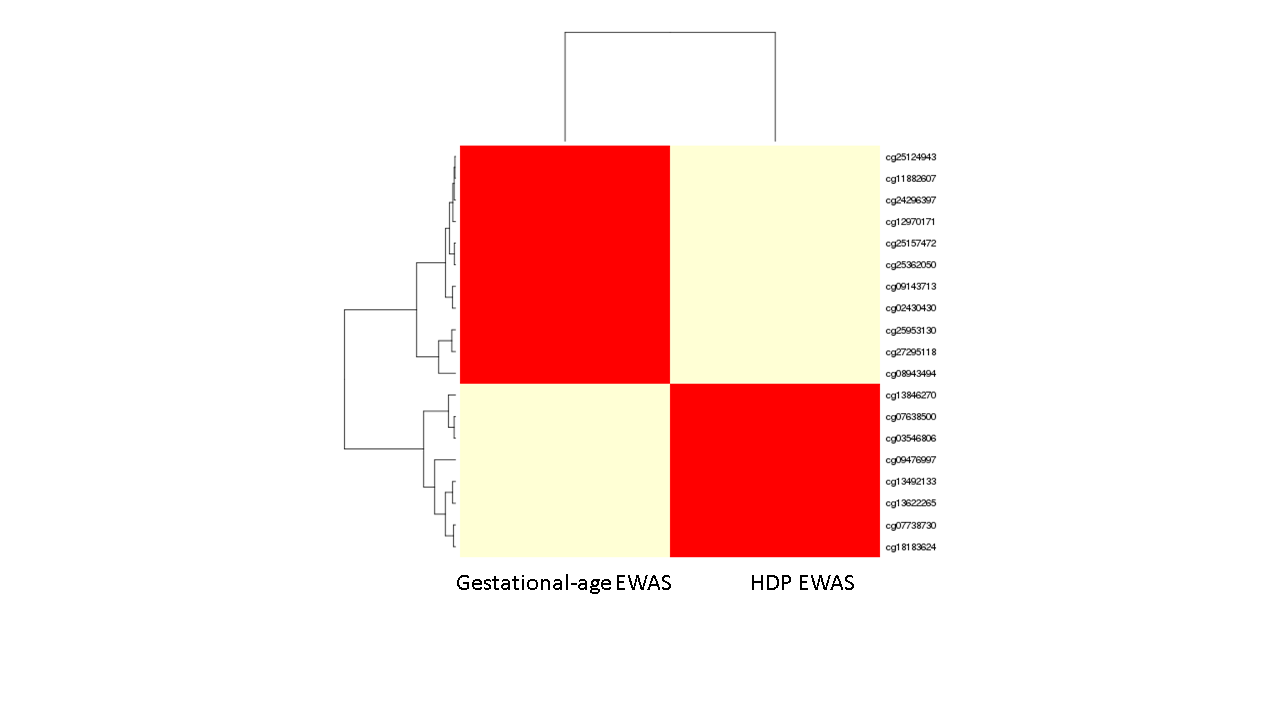** |

**Figure S8**. Methylation over time for offspring of HDP mothers

| 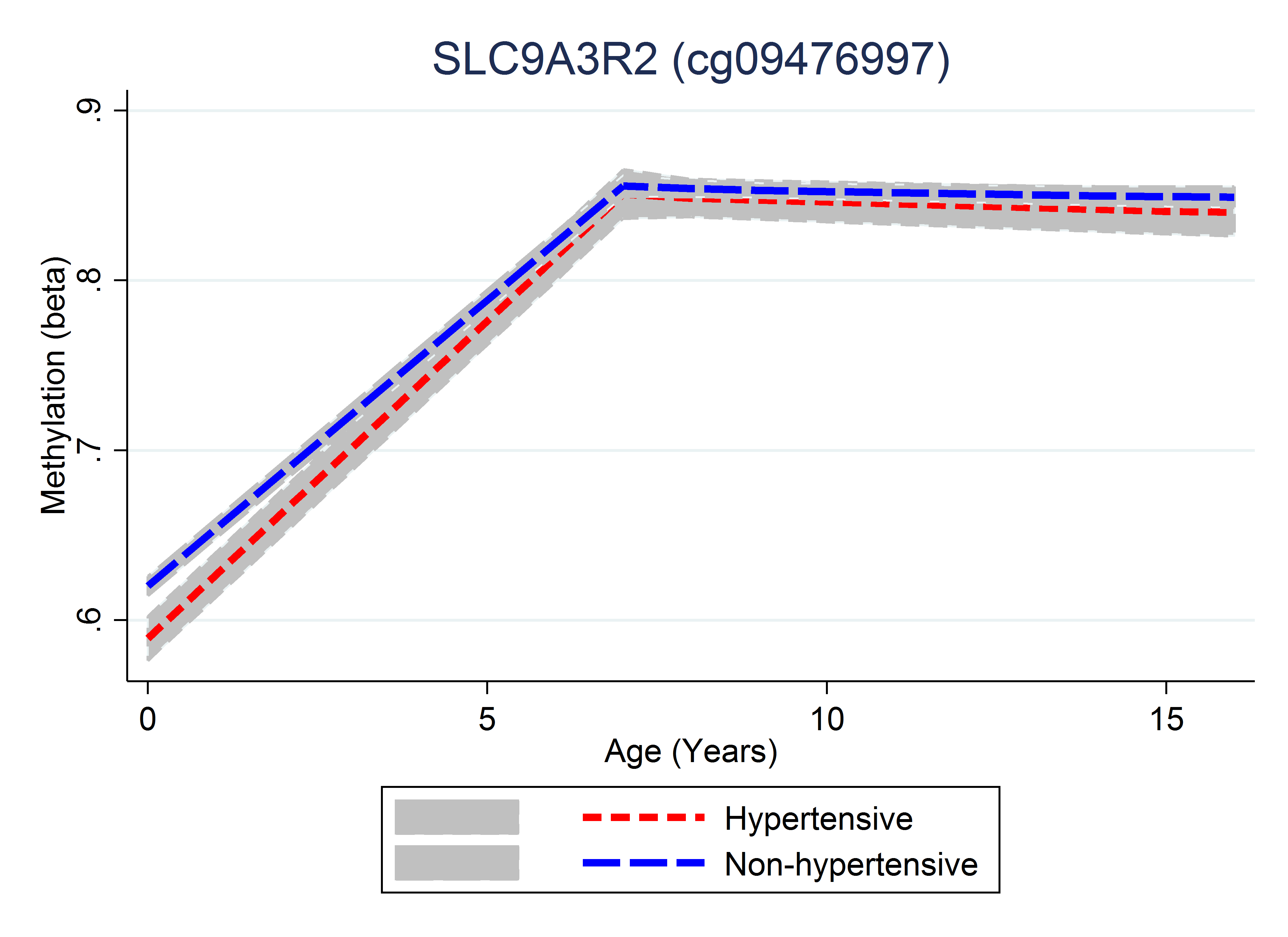 | 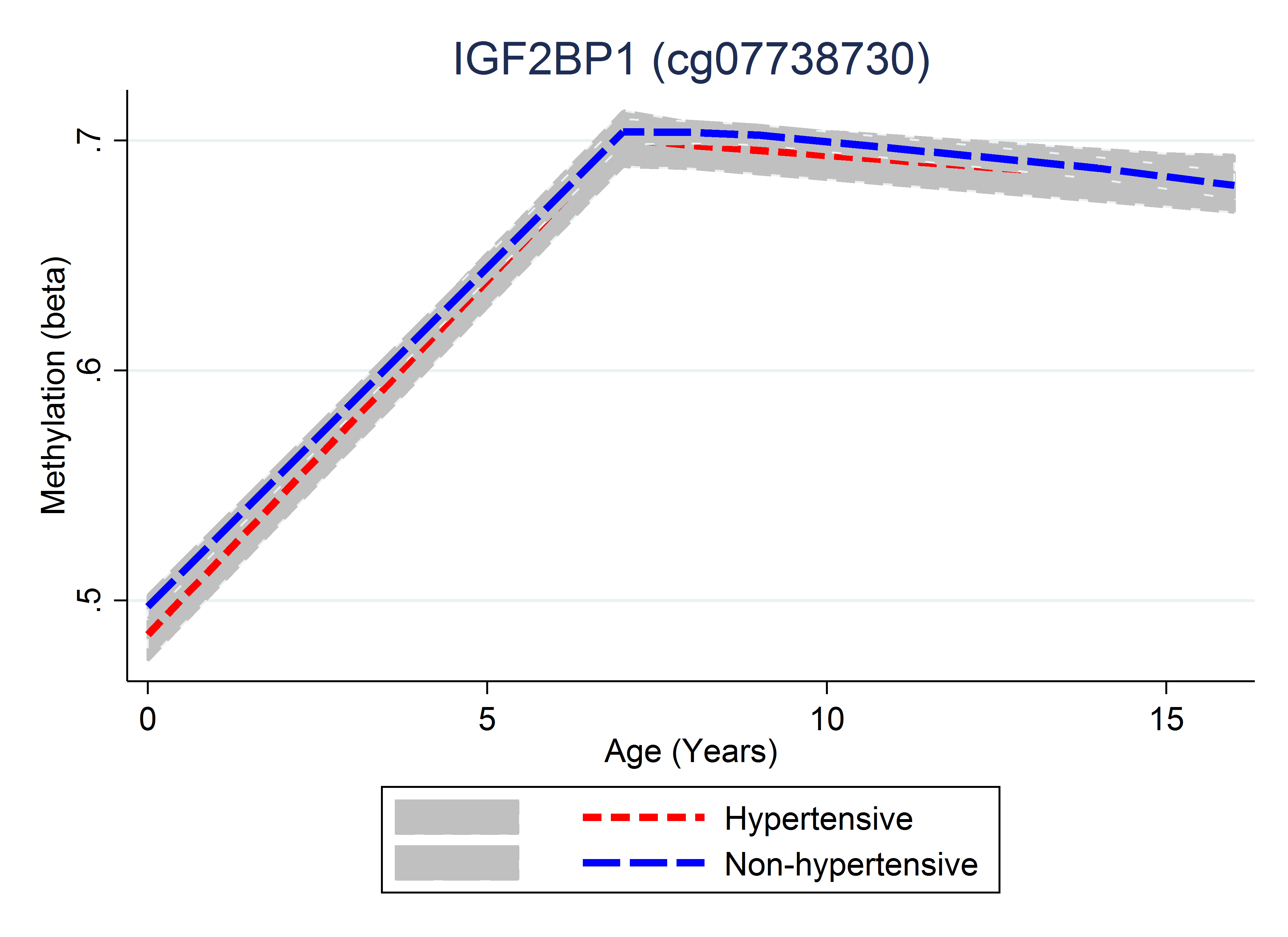 |
| --- | --- |
| 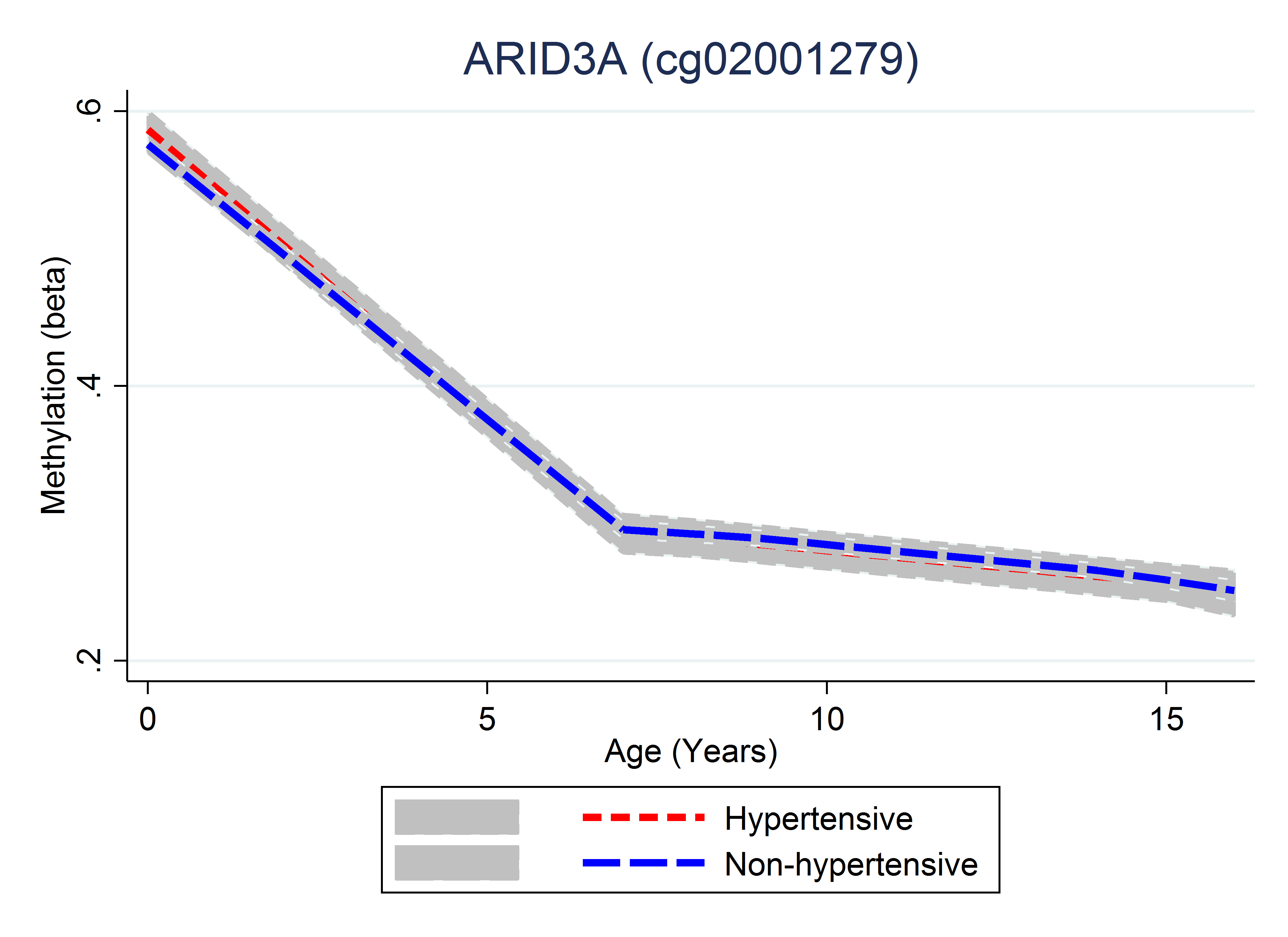 | 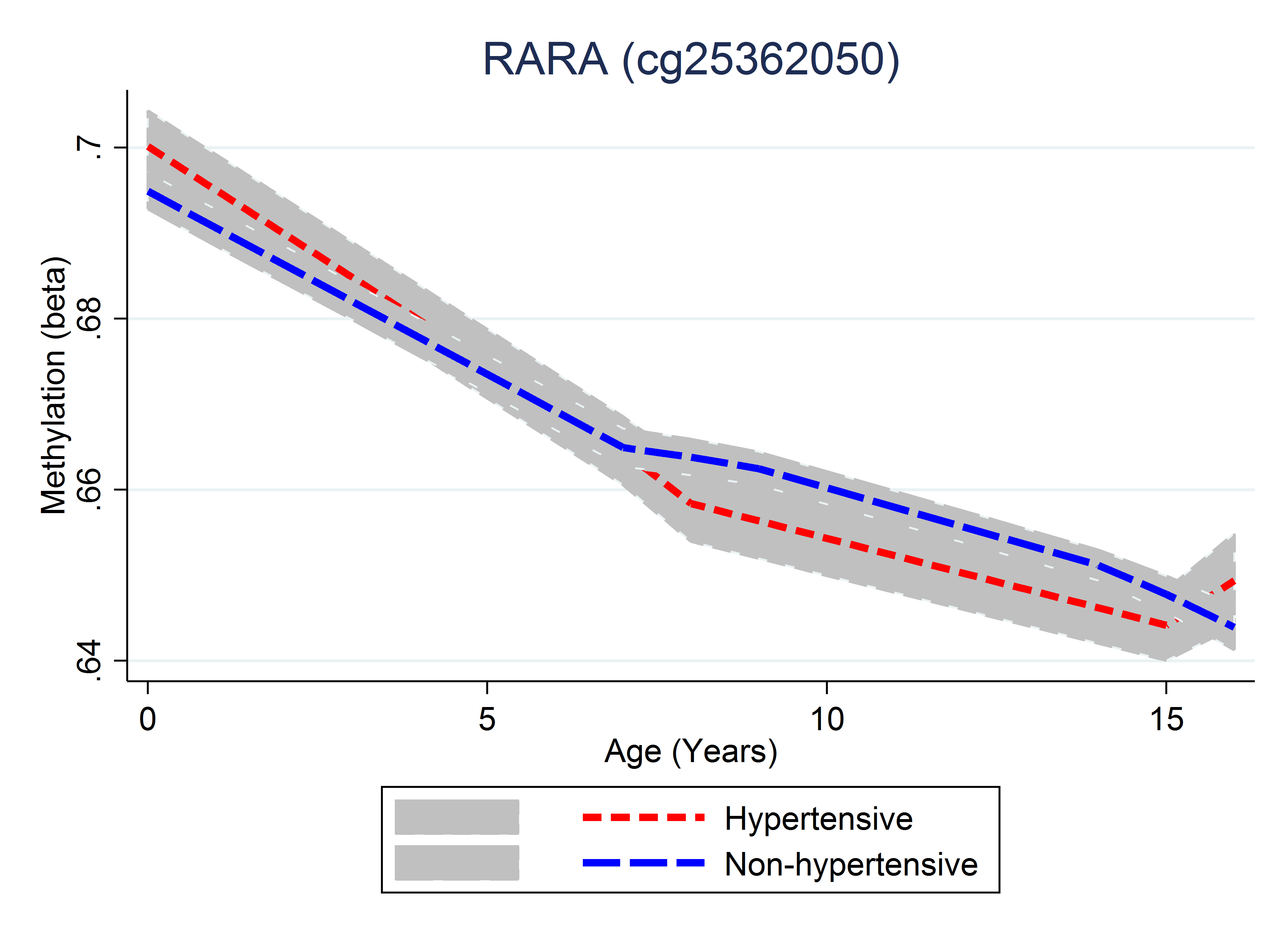 |

Methylation (proportion of methylated cells) over time for offspring of HDP mothers (dashed (red) line) compared with offspring of non-HDP mothers (dashed (blue) line). Ribbons indicate 95% confidence intervals.
