# Supplementary figures and images for: Hypertensive Disorders of Pregnancy and DNA Methylation in Newborns: Findings From the Pregnancy and Childhood Epigenetics Consortium
Source: Hypertension. 2019 Jun 24;74(2):375–83. doi: 10.1161/HYPERTENSIONAHA.119.12634 (PMC6635125; doi:10.1161/HYPERTENSIONAHA.119.12634)

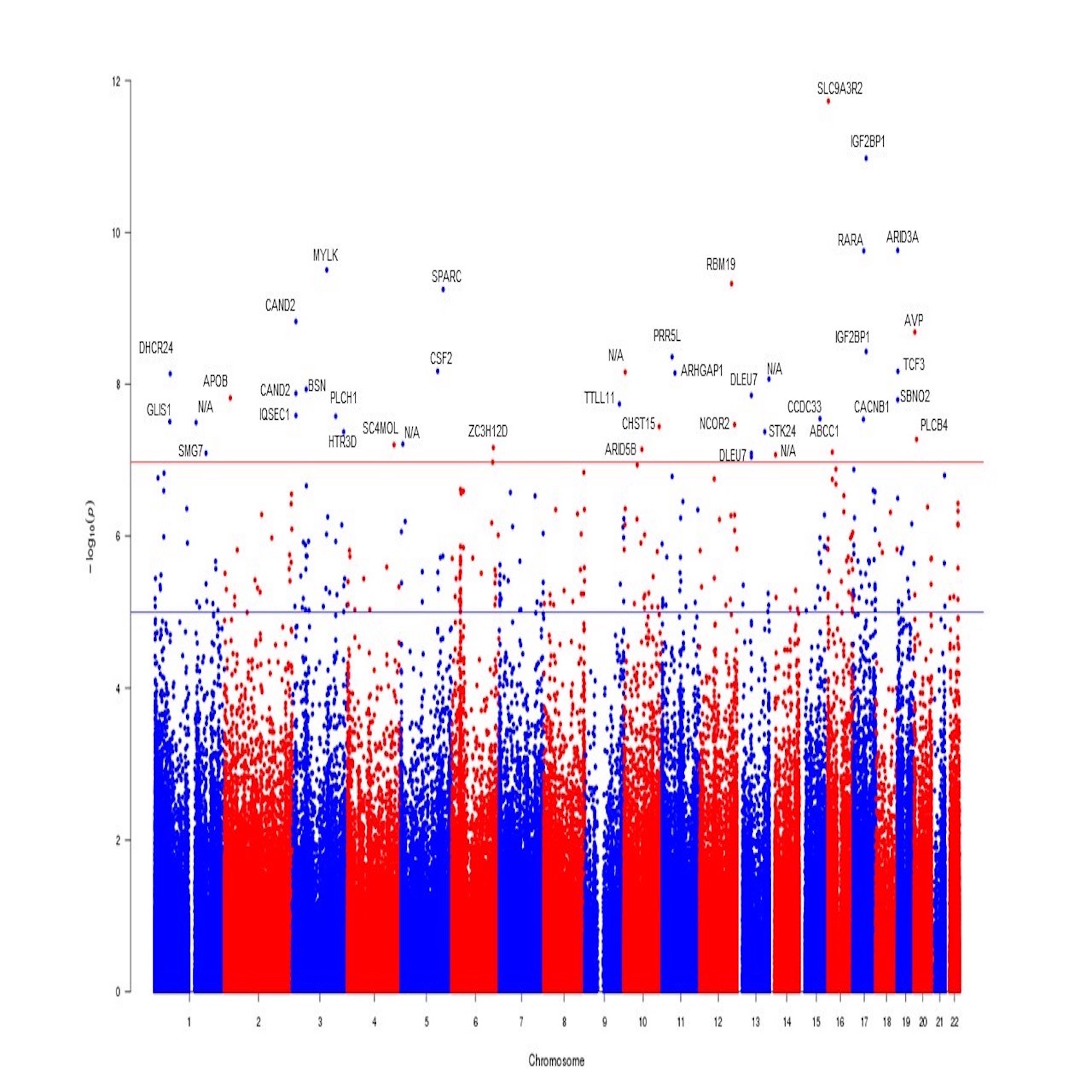

Supplement: Supplementary file 2 [file hyp-74-375-s002.jpg]
