# Supplementary material for: Hypertensive Disorders of Pregnancy and DNA Methylation in Newborns: Findings From the Pregnancy and Childhood Epigenetics Consortium
Source: Hypertension. 2019 Jun 24;74(2):375–83. doi: 10.1161/HYPERTENSIONAHA.119.12634 (PMC6635125; doi:10.1161/HYPERTENSIONAHA.119.12634)
Supplement: Supplementary file 3 [file hyp-74-375-s003.pdf]

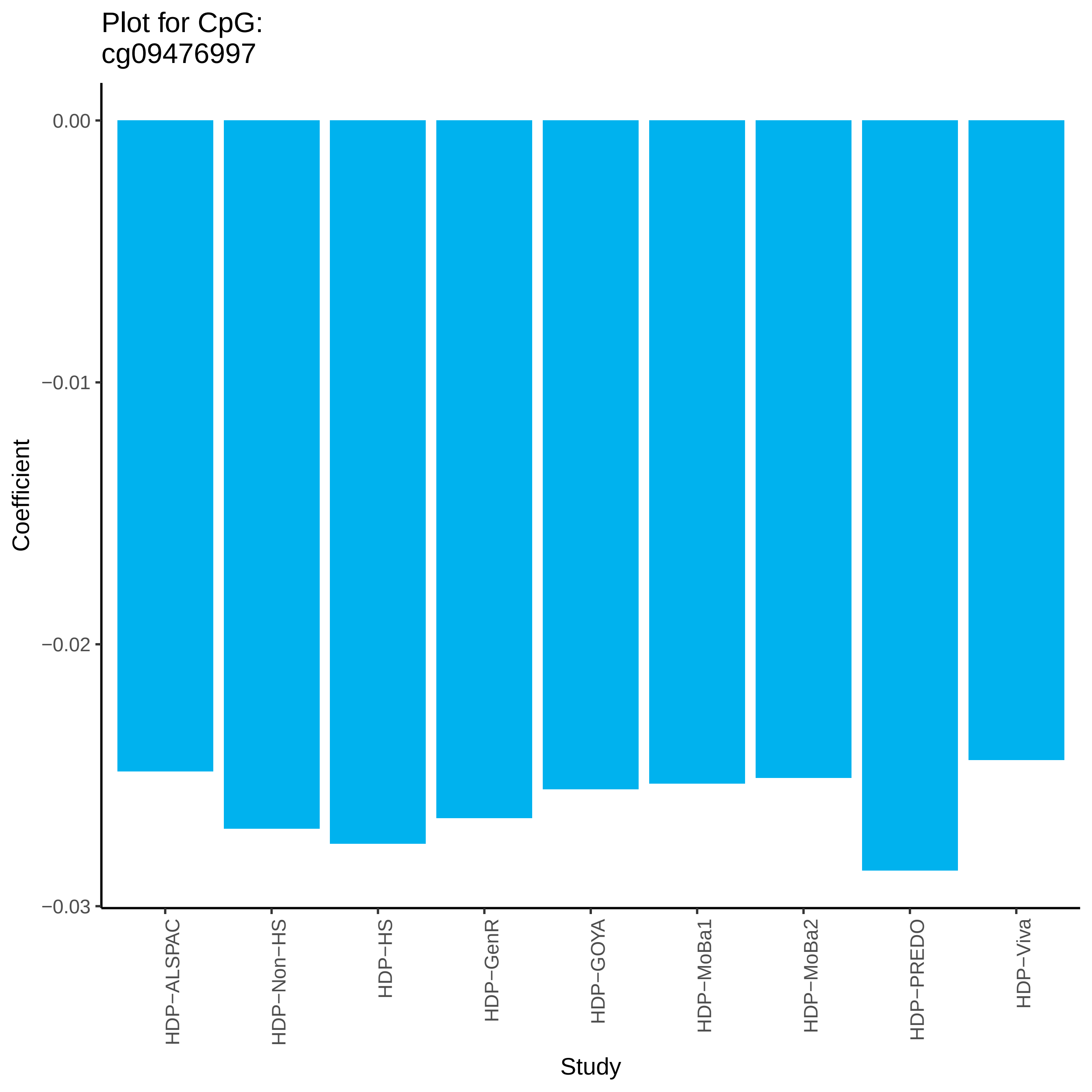

Plot for CpG:
cg09476997
0.00
−0.01
−0.02
−0.03
Coefficient
HDP−ALSPAC
HDP−Non−HS
HDP−HS
HDP−GenR
HDP−GOYA
HDP−MoBa1
HDP−MoBa2
HDP−PREDO
HDP−Viva
Study

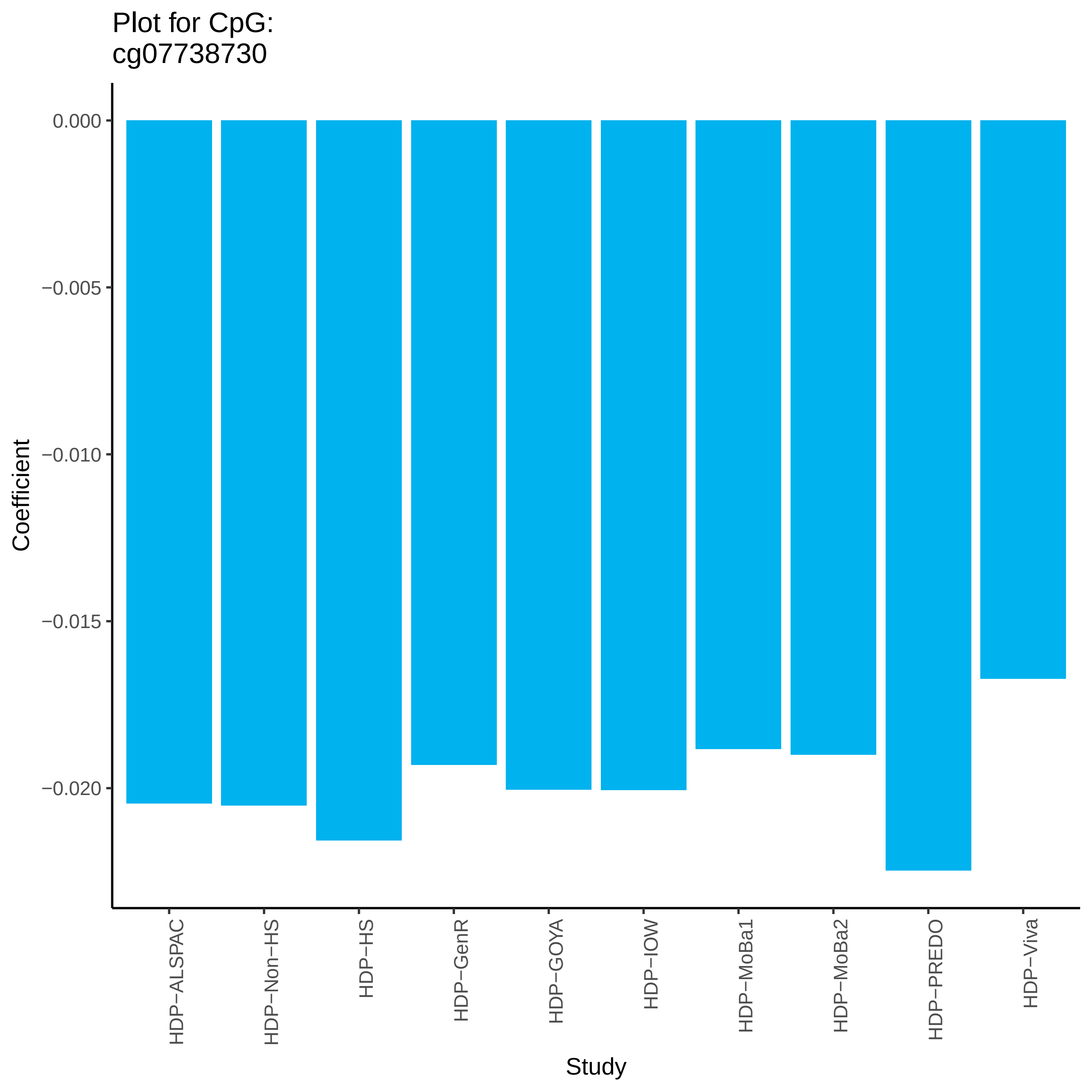
Plot for CpG:
cg07738730
Coefficient
0.000
−0.005
−0.010
−0.015
−0.020
HDP−ALSPAC
HDP−Non−HS
HDP−HS
HDP−GenR
HDP−GOYA
HDP−IOW
HDP−MoBa1
HDP−MoBa2
HDP−PREDO
HDP−Viva
Study

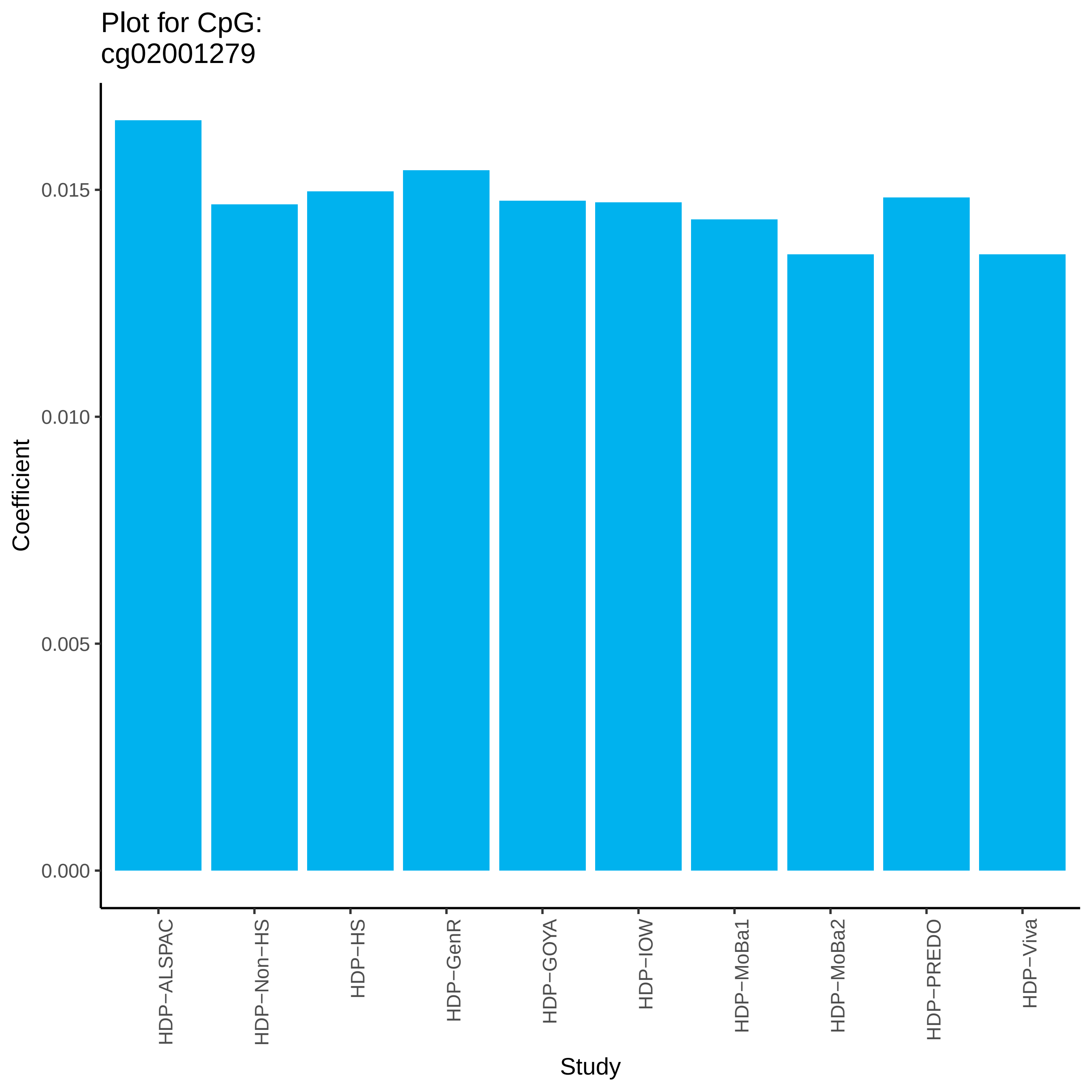

Plot for CpG:
cg02001279
Coefficient
0.000
0.005
0.010
0.015
Study
HDP−ALSPAC
HDP−Non−HS
HDP−HS
HDP−GenR
HDP−GOYA
HDP−IOW
HDP−MoBa1
HDP−MoBa2
HDP−PREDO
HDP−Viva

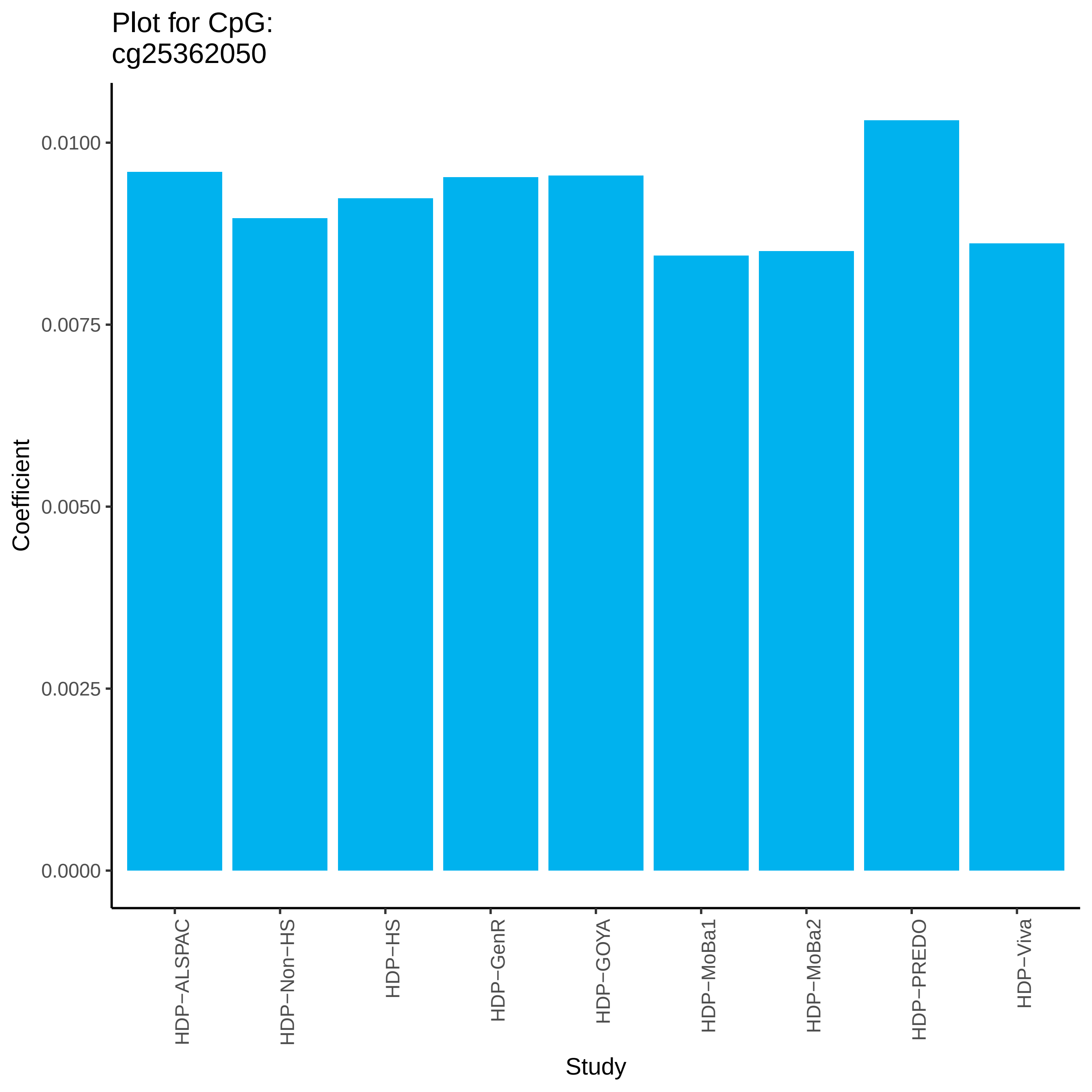
Plot for CpG:
cg25362050
Coefficient
0.0100
0.0075
0.0050
0.0025
0.0000
HDP−ALSPAC
HDP−Non−HS
HDP−HS
HDP−GenR
HDP−GOYA
HDP−MoBa1
HDP−MoBa2
HDP−PREDO
HDP−Viva
Study

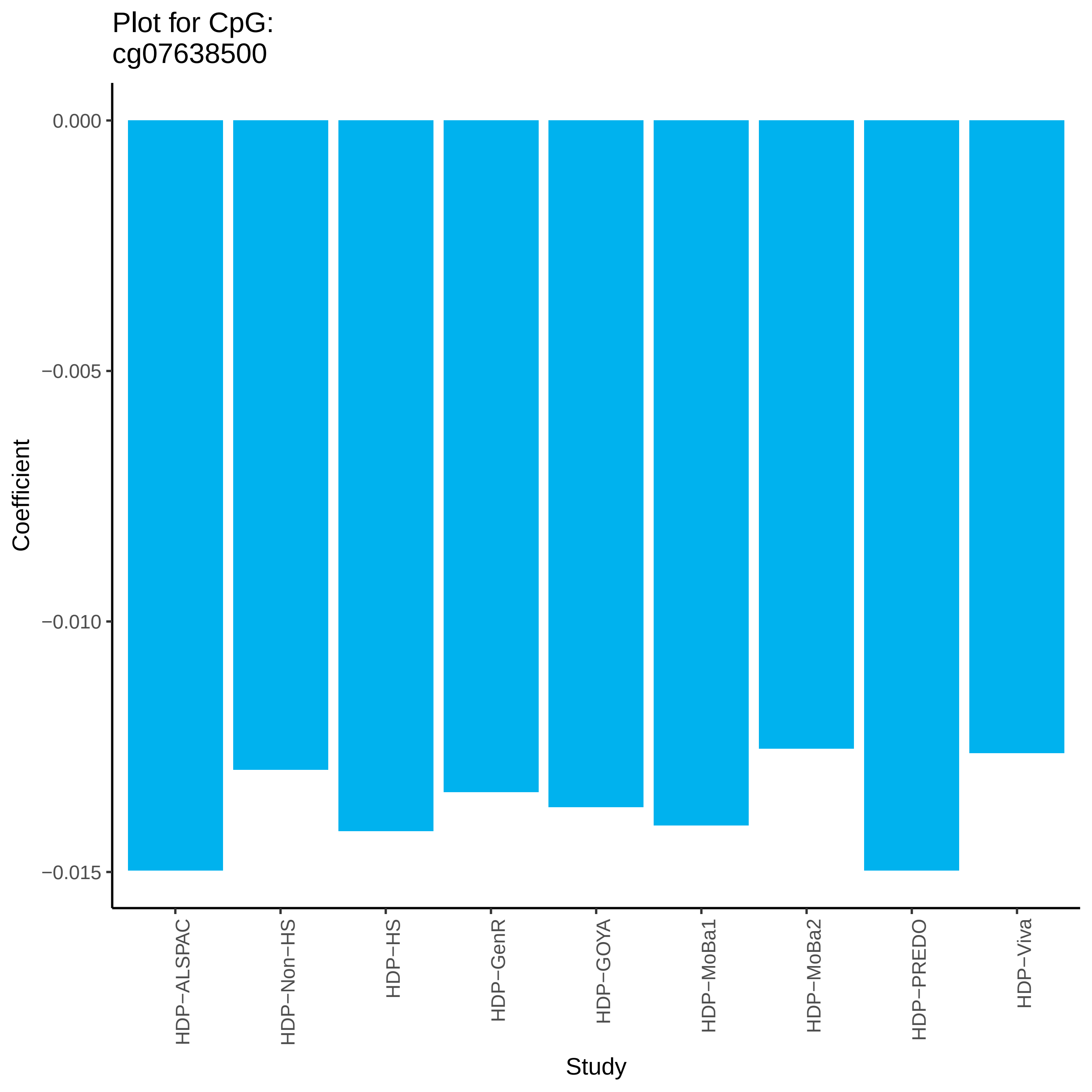
Plot for CpG:
cg07638500
0.000
−0.005
−0.010
−0.015
Coefficient
HDP−ALSPAC
HDP−Non−HS
HDP−HS
HDP−GenR
HDP−GOYA
HDP−MoBa1
HDP−MoBa2
HDP−PREDO
HDP−Viva
Study

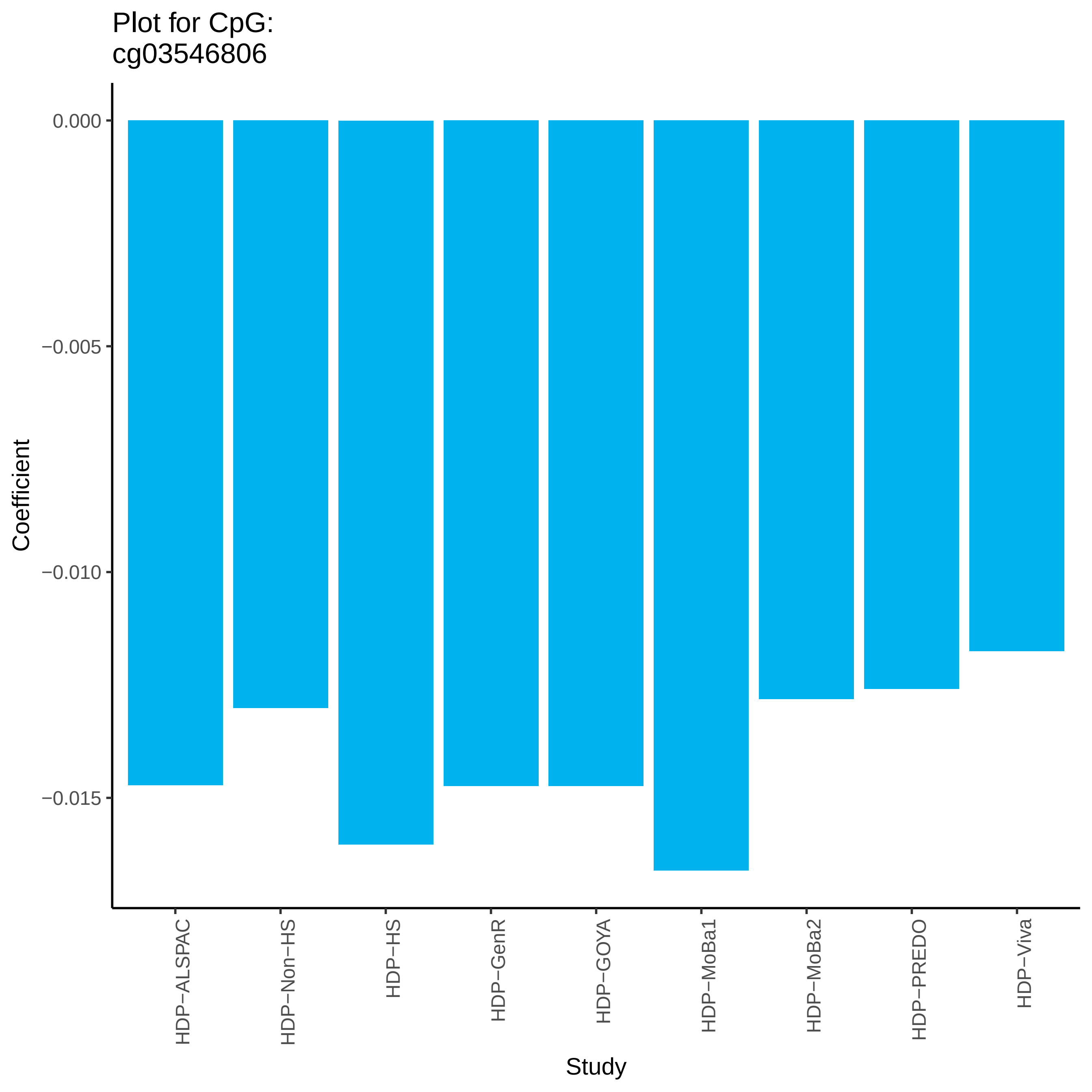
Plot for CpG:
cg03546806
0.000
−0.005
−0.010
−0.015
Coefficient
HDP−ALSPAC
HDP−Non−HS
HDP−HS
HDP−GenR
HDP−GOYA
HDP−MoBa1
HDP−MoBa2
HDP−PREDO
HDP−Viva
Study

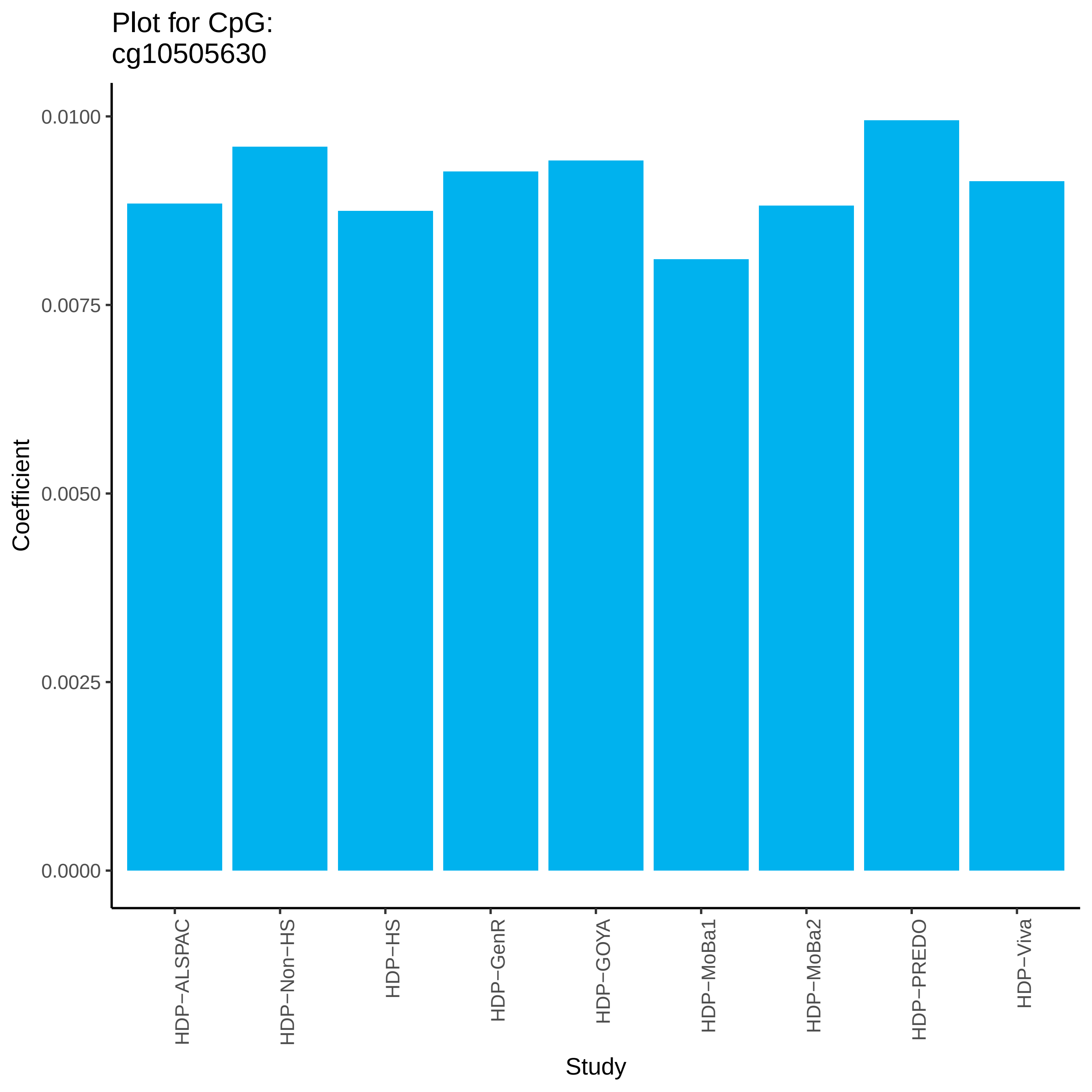

Plot for CpG:
cg10505630
Coefficient
0.0100
0.0075
0.0050
0.0025
0.0000
HDP−ALSPAC
HDP−Non−HS
HDP−HS
HDP−GenR
HDP−GOYA
HDP−MoBa1
HDP−MoBa2
HDP−PREDO
HDP−Viva
Study

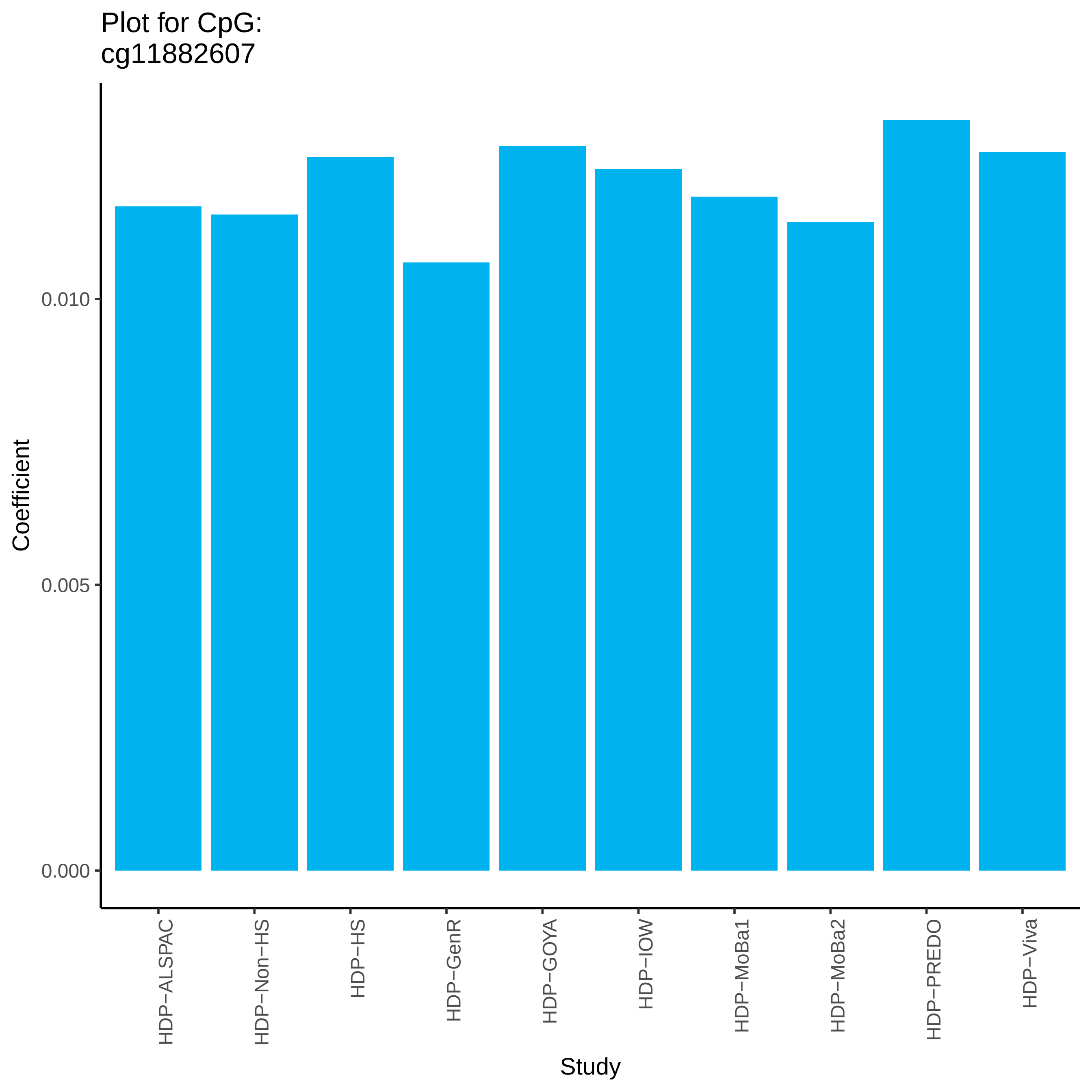

Plot for CpG:
cg11882607
Coefficient
0.010
0.005
0.000
HDP−ALSPAC
HDP−Non−HS
HDP−HS
HDP−GenR
HDP−GOYA
HDP−IOW
HDP−MoBa1
HDP−MoBa2
HDP−PREDO
HDP−Viva
Study

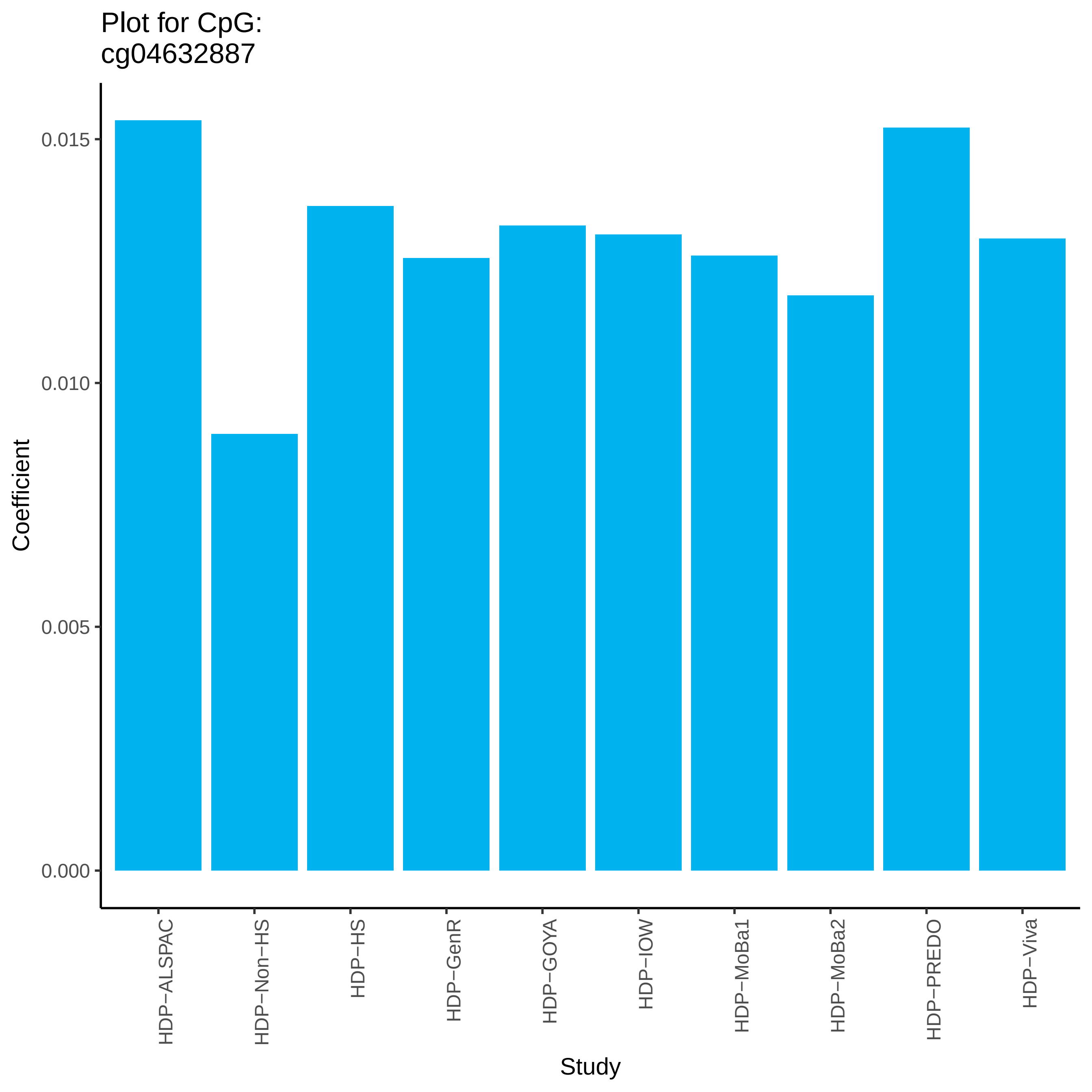
Plot for CpG:
cg04632887
Coefficient
0.015
0.010
0.005
0.000
HDP−ALSPAC
HDP−Non−HS
HDP−HS
HDP−GenR
HDP−GOYA
HDP−IOW
HDP−MoBa1
HDP−MoBa2
HDP−PREDO
HDP−Viva
Study

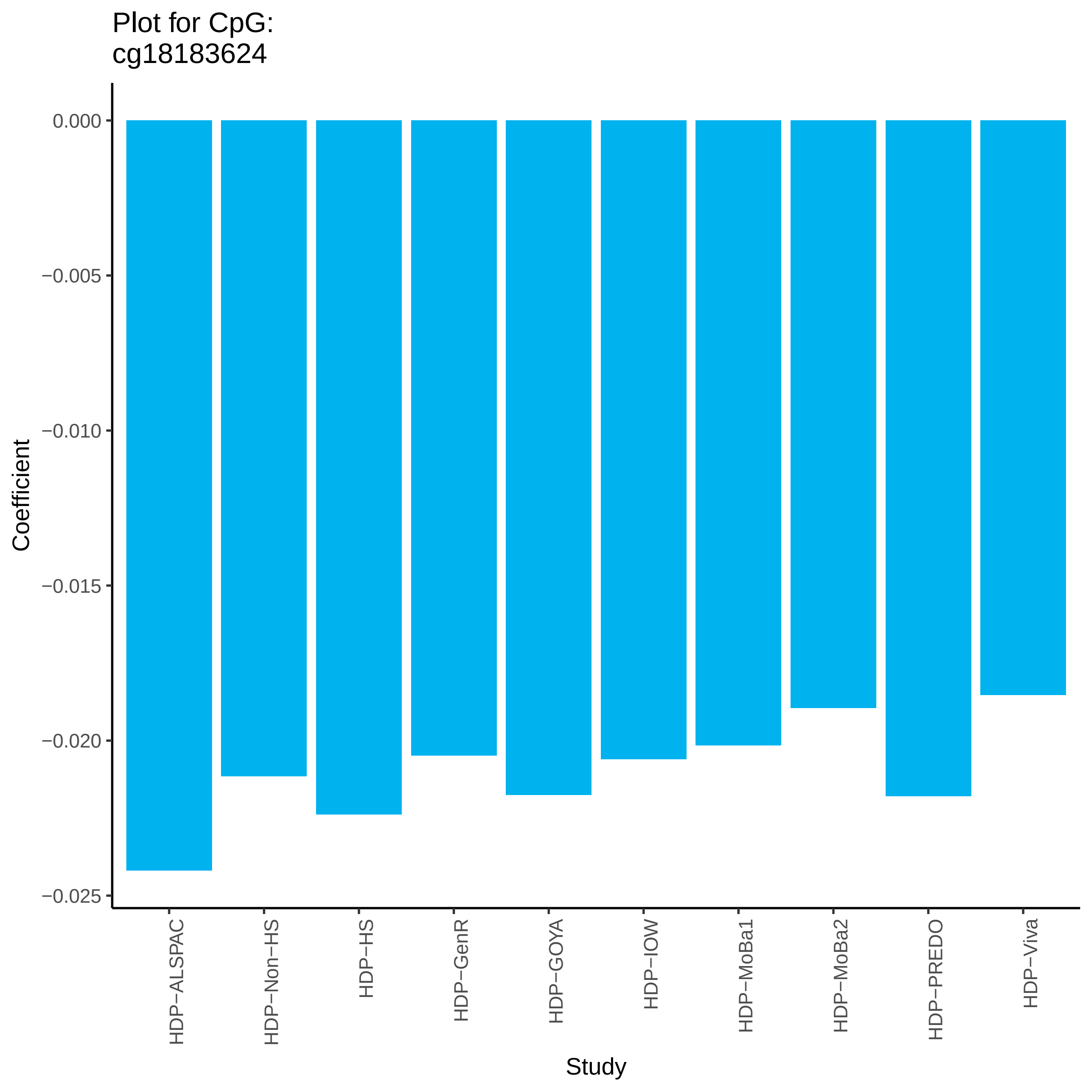

Plot for CpG:
cg18183624
Coefficient
0.000
−0.005
−0.010
−0.015
−0.020
−0.025
HDP−ALSPAC
HDP−Non−HS
HDP−HS
HDP−GenR
HDP−GOYA
HDP−IOW
HDP−MoBa1
HDP−MoBa2
HDP−PREDO
HDP−Viva
Study

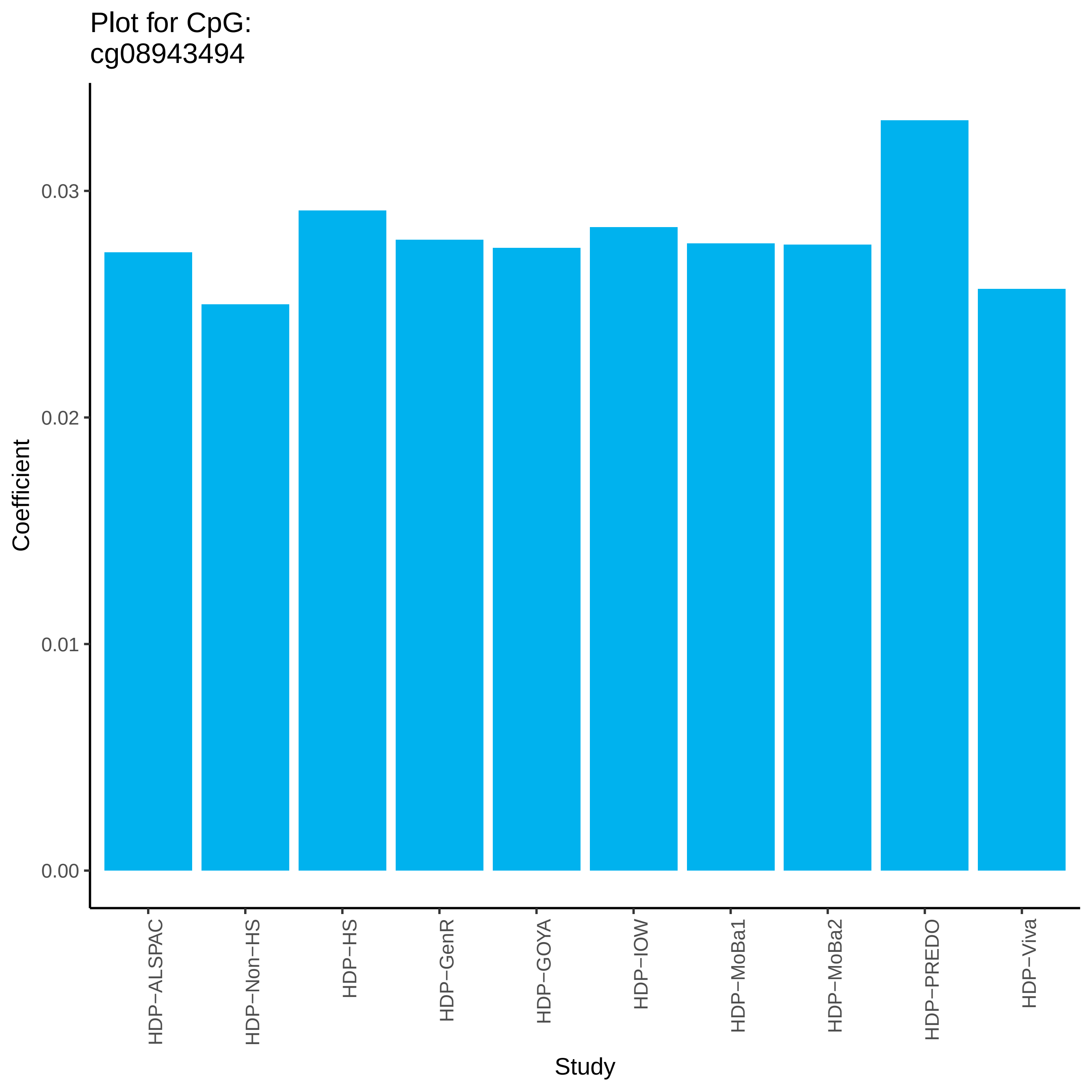
Plot for CpG:
cg08943494
Coefficient
0.03
0.02
0.01
0.00
HDP−ALSPAC
HDP−Non−HS
HDP−HS
HDP−GenR
HDP−GOYA
HDP−IOW
HDP−MoBa1
HDP−MoBa2
HDP−PREDO
HDP−Viva
Study

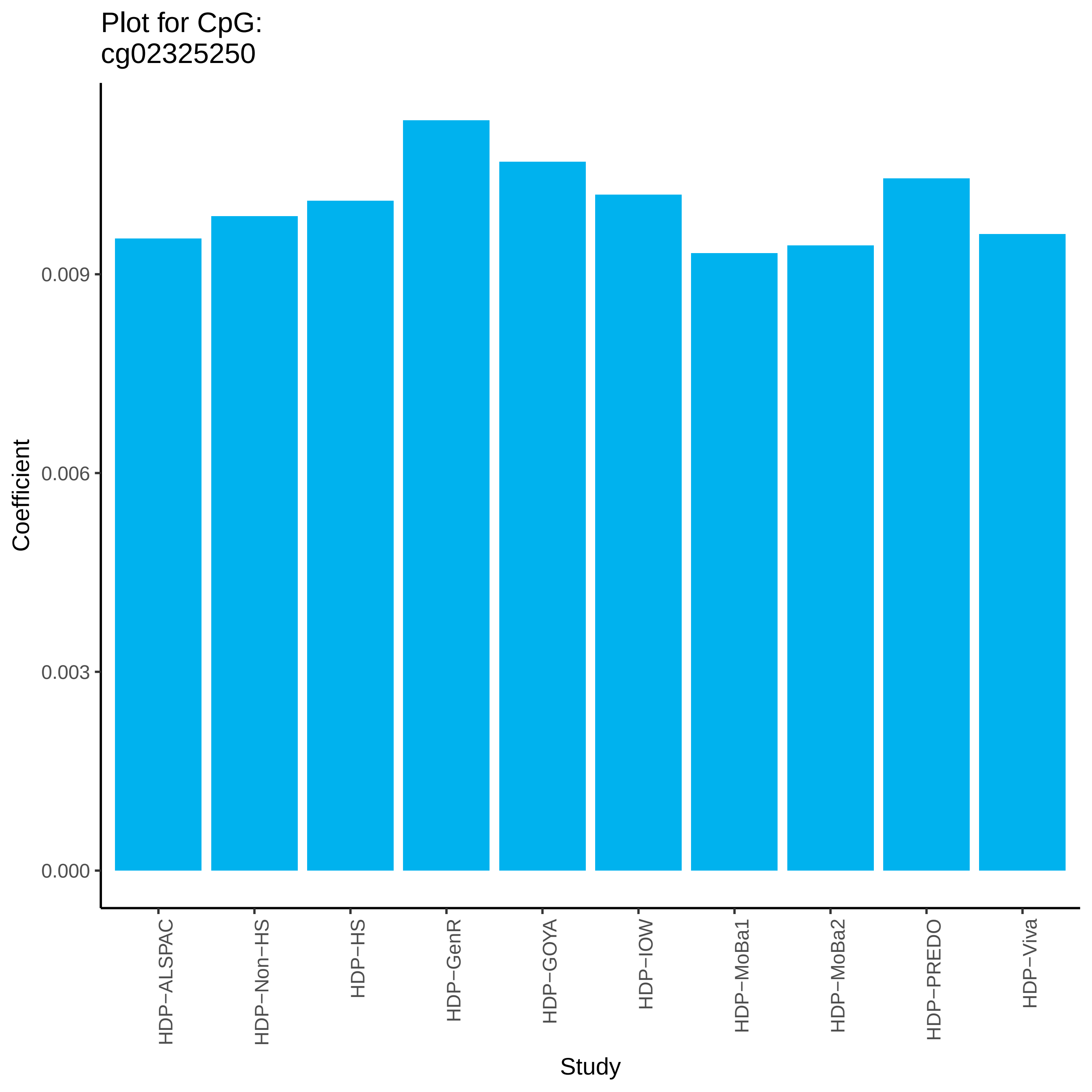

Plot for CpG:
cg02325250
Coefficient
0.009
0.006
0.003
0.000
HDP−ALSPAC
HDP−Non−HS
HDP−HS
HDP−GenR
HDP−GOYA
HDP−IOW
HDP−MoBa1
HDP−MoBa2
HDP−PREDO
HDP−Viva
Study

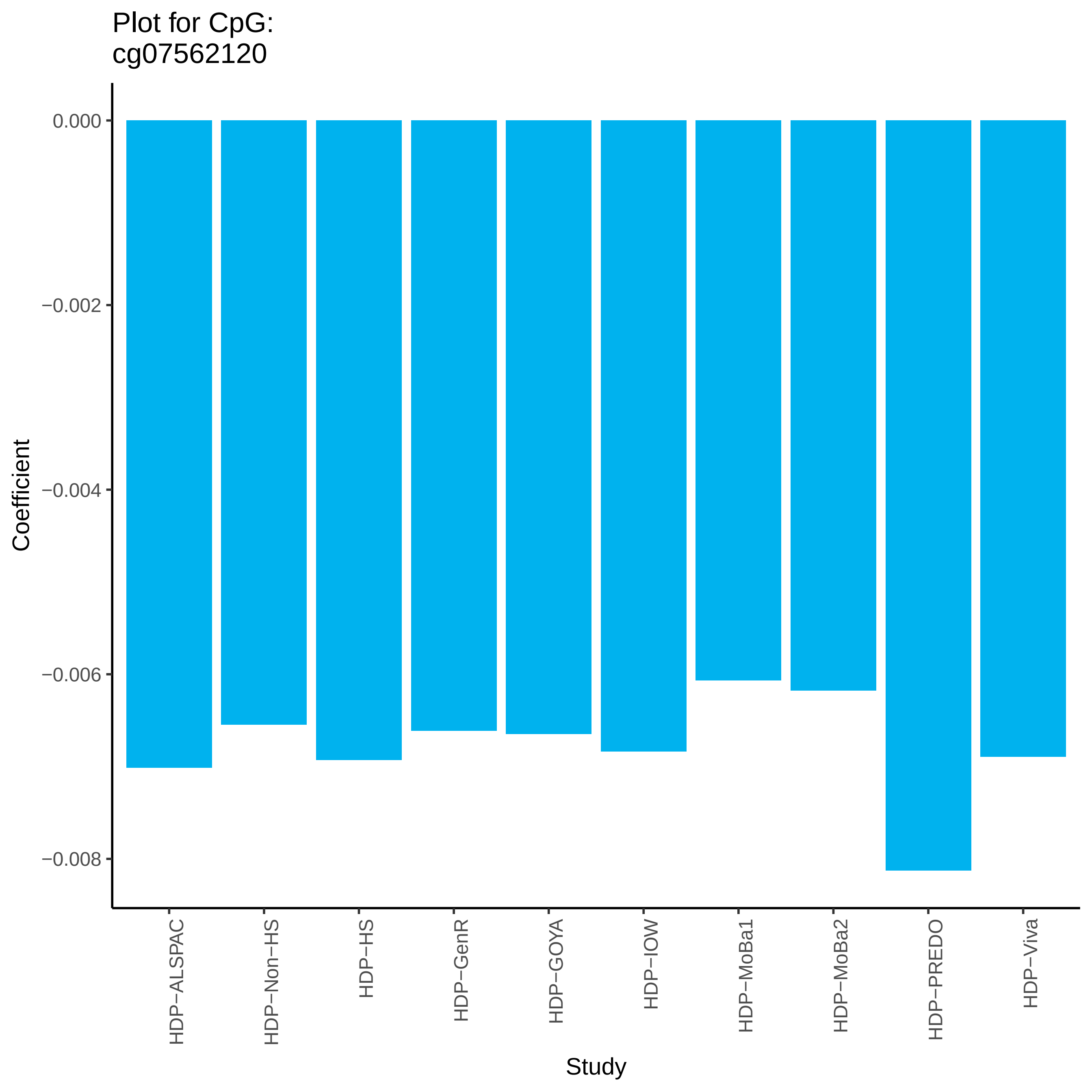

Plot for CpG:
cg07562120
Coefficient
0.000
−0.002
−0.004
−0.006
−0.008
HDP−ALSPAC
HDP−Non−HS
HDP−HS
HDP−GenR
HDP−GOYA
HDP−IOW
HDP−MoBa1
HDP−MoBa2
HDP−PREDO
HDP−Viva
Study

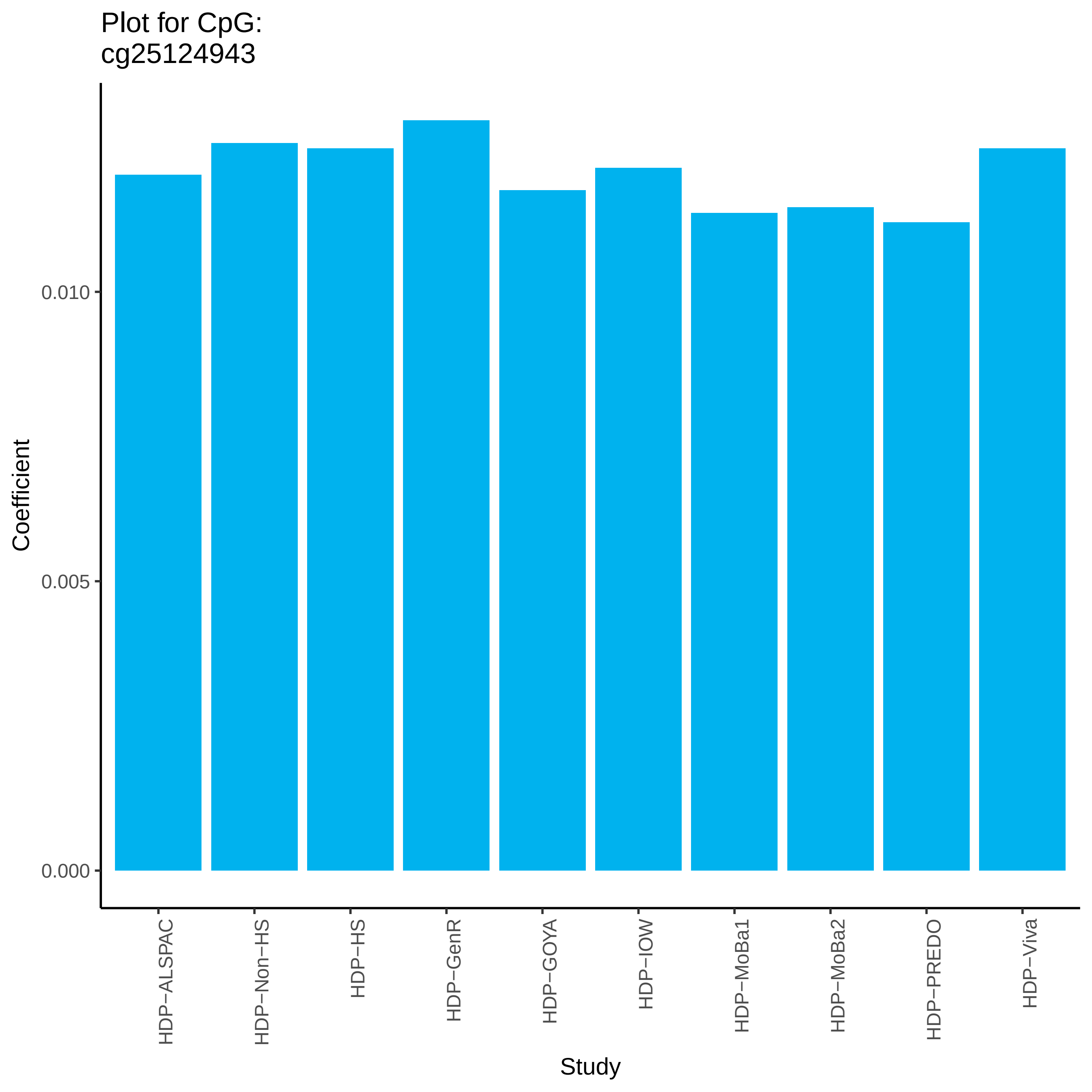

Plot for CpG:
cg25124943
Coefficient
0.010
0.005
0.000
HDP−ALSPAC
HDP−Non−HS
HDP−HS
HDP−GenR
HDP−GOYA
HDP−IOW
HDP−MoBa1
HDP−MoBa2
HDP−PREDO
HDP−Viva
Study

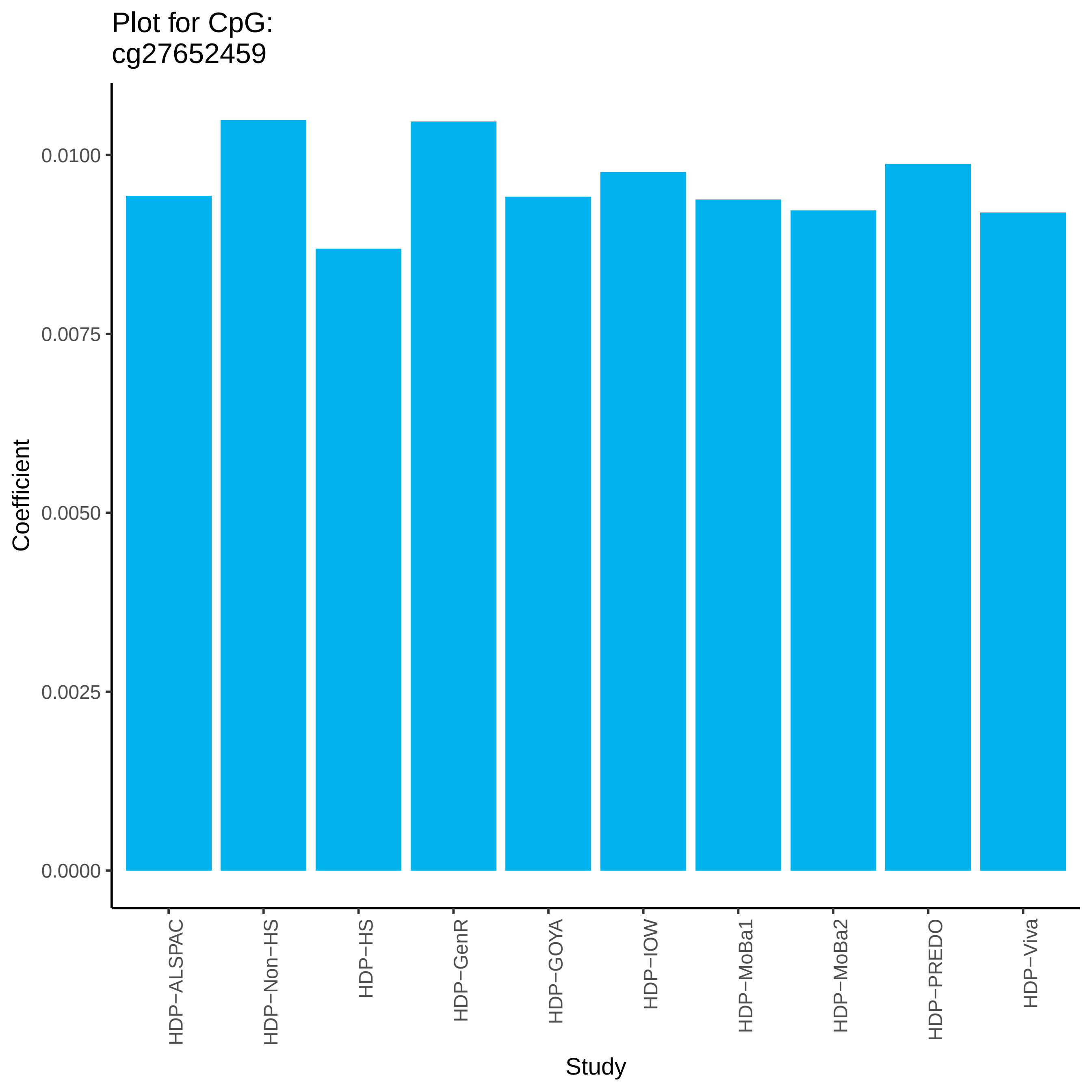

Plot for CpG:
cg27652459
Coefficient
0.0100
0.0075
0.0050
0.0025
0.0000
HDP−ALSPAC
HDP−Non−HS
HDP−HS
HDP−GenR
HDP−GOYA
HDP−IOW
HDP−MoBa1
HDP−MoBa2
HDP−PREDO
HDP−Viva
Study

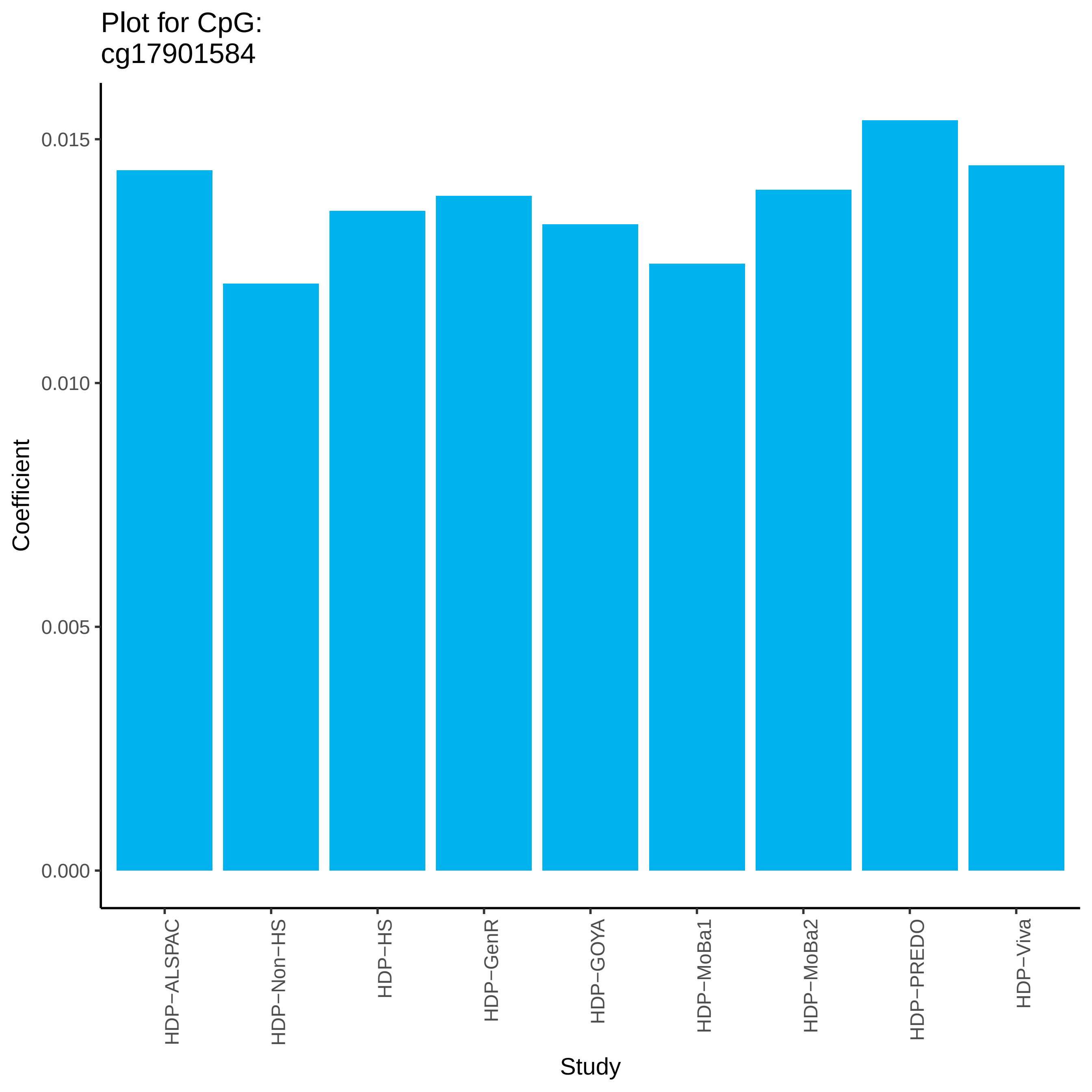

Plot for CpG:
cg17901584
Coefficient
0.015
0.010
0.005
0.000
HDP−ALSPAC
HDP−Non−HS
HDP−HS
HDP−GenR
HDP−GOYA
HDP−MoBa1
HDP−MoBa2
HDP−PREDO
HDP−Viva
Study

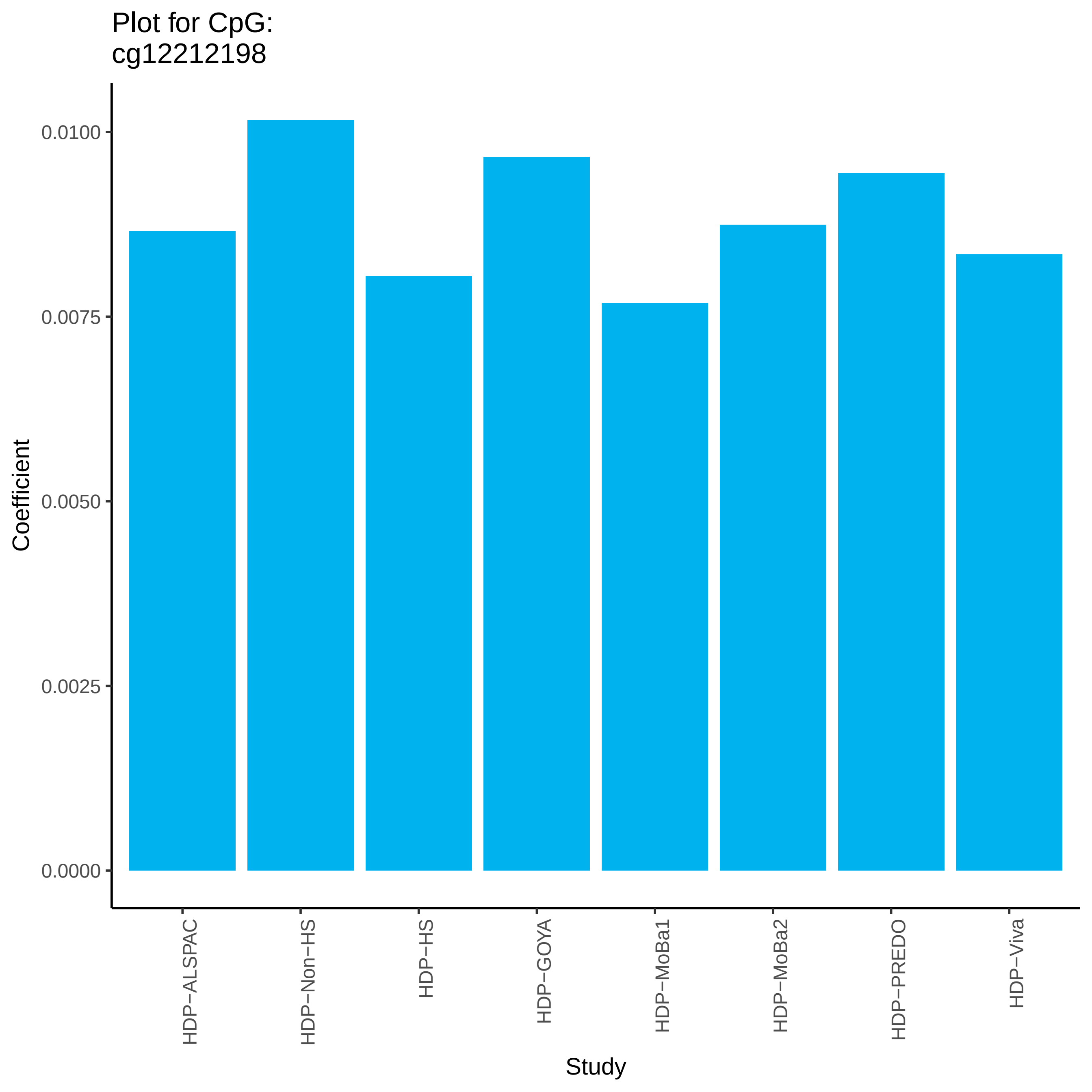

Plot for CpG:
cg12212198
Coefficient
0.0100
0.0075
0.0050
0.0025
0.0000
HDP−ALSPAC
HDP−Non−HS
HDP−HS
HDP−GOYA
HDP−MoBa1
HDP−MoBa2
HDP−PREDO
HDP−Viva
Study

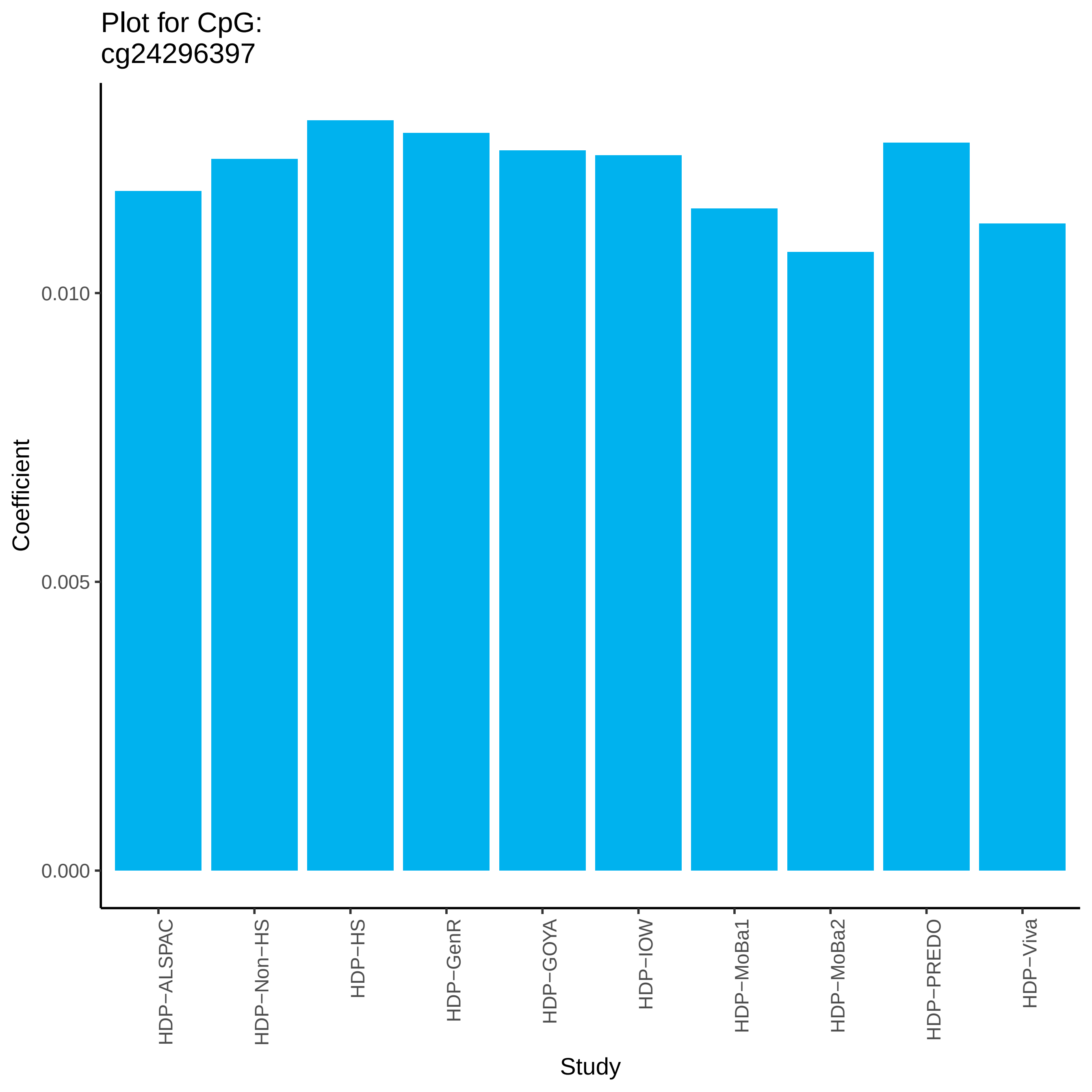

Plot for CpG:
cg24296397
Coefficient
0.010
0.005
0.000
HDP−ALSPAC
HDP−Non−HS
HDP−HS
HDP−GenR
HDP−GOYA
HDP−IOW
HDP−MoBa1
HDP−MoBa2
HDP−PREDO
HDP−Viva
Study

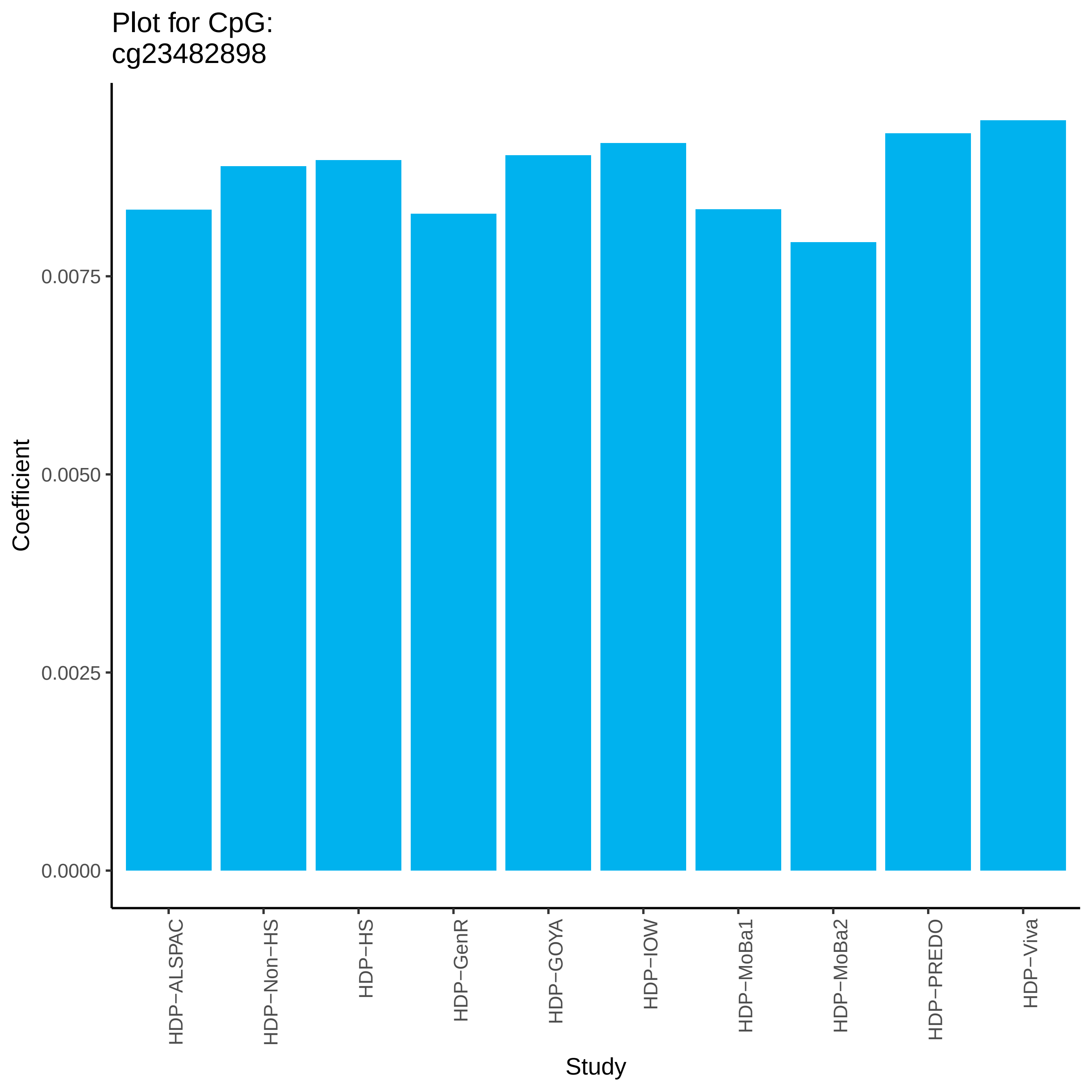

Plot for CpG:
cg23482898
Coefficient
0.0075
0.0050
0.0025
0.0000
HDP−ALSPAC
HDP−Non−HS
HDP−HS
HDP−GenR
HDP−GOYA
HDP−IOW
HDP−MoBa1
HDP−MoBa2
HDP−PREDO
HDP−Viva
Study

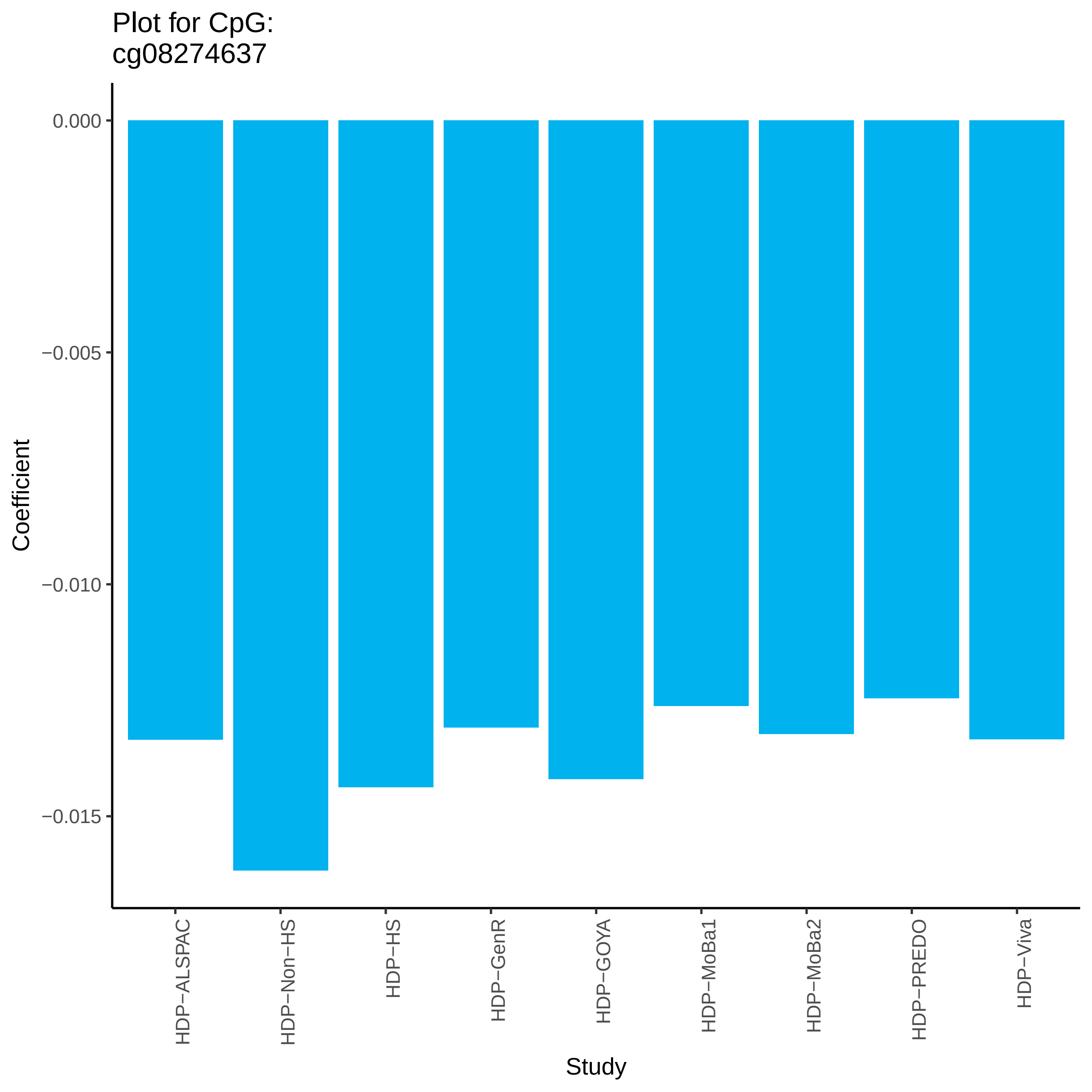

Plot for CpG:
cg08274637
Coefficient
0.000
−0.005
−0.010
−0.015
HDP−ALSPAC
HDP−Non−HS
HDP−HS
HDP−GenR
HDP−GOYA
HDP−MoBa1
HDP−MoBa2
HDP−PREDO
HDP−Viva
Study

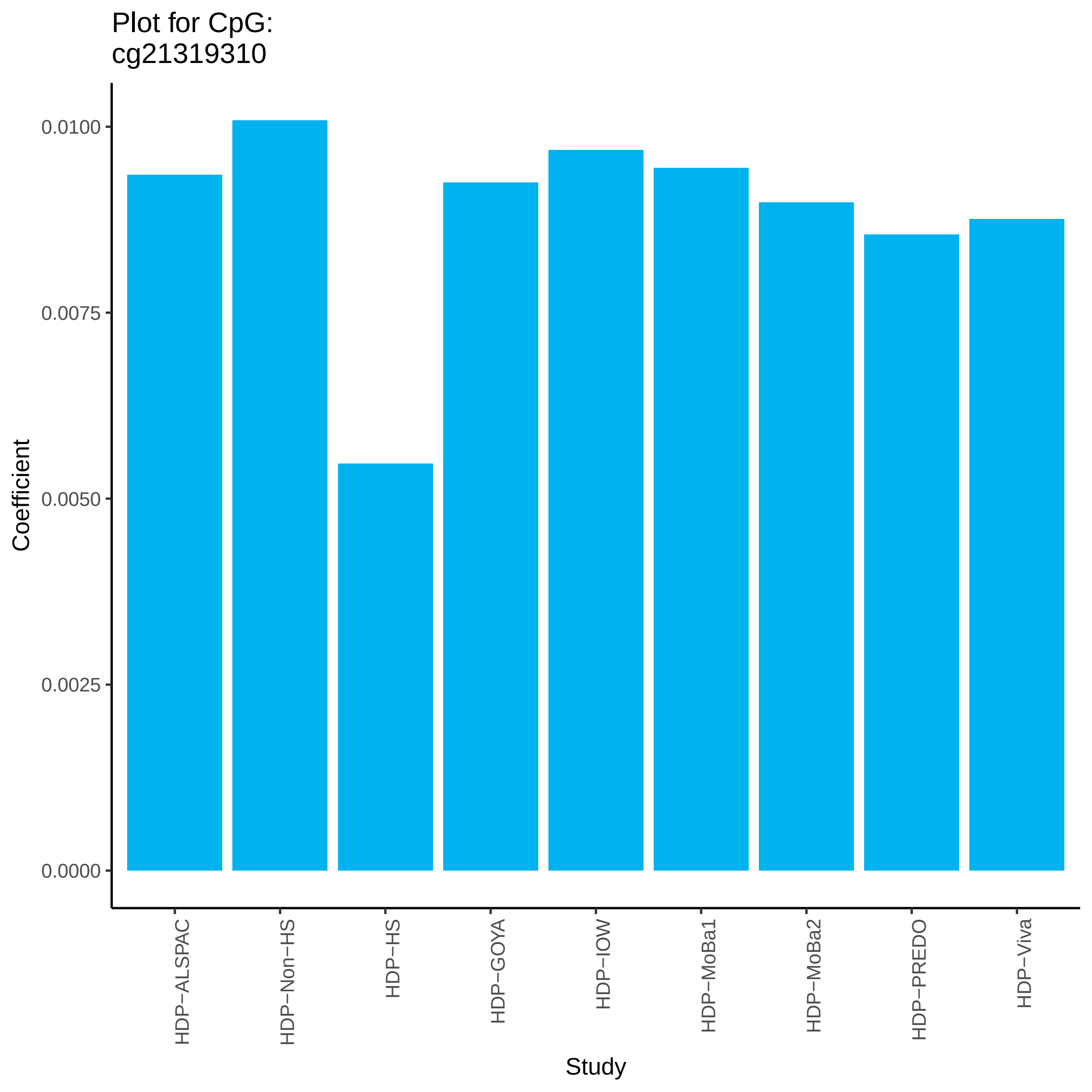

Plot for CpG:
cg21319310
Coefficient
0.0100
0.0075
0.0050
0.0025
0.0000
HDP−ALSPAC
HDP−Non−HS
HDP−HS
HDP−GOYA
HDP−IOW
HDP−MoBa1
HDP−MoBa2
HDP−PREDO
HDP−Viva
Study

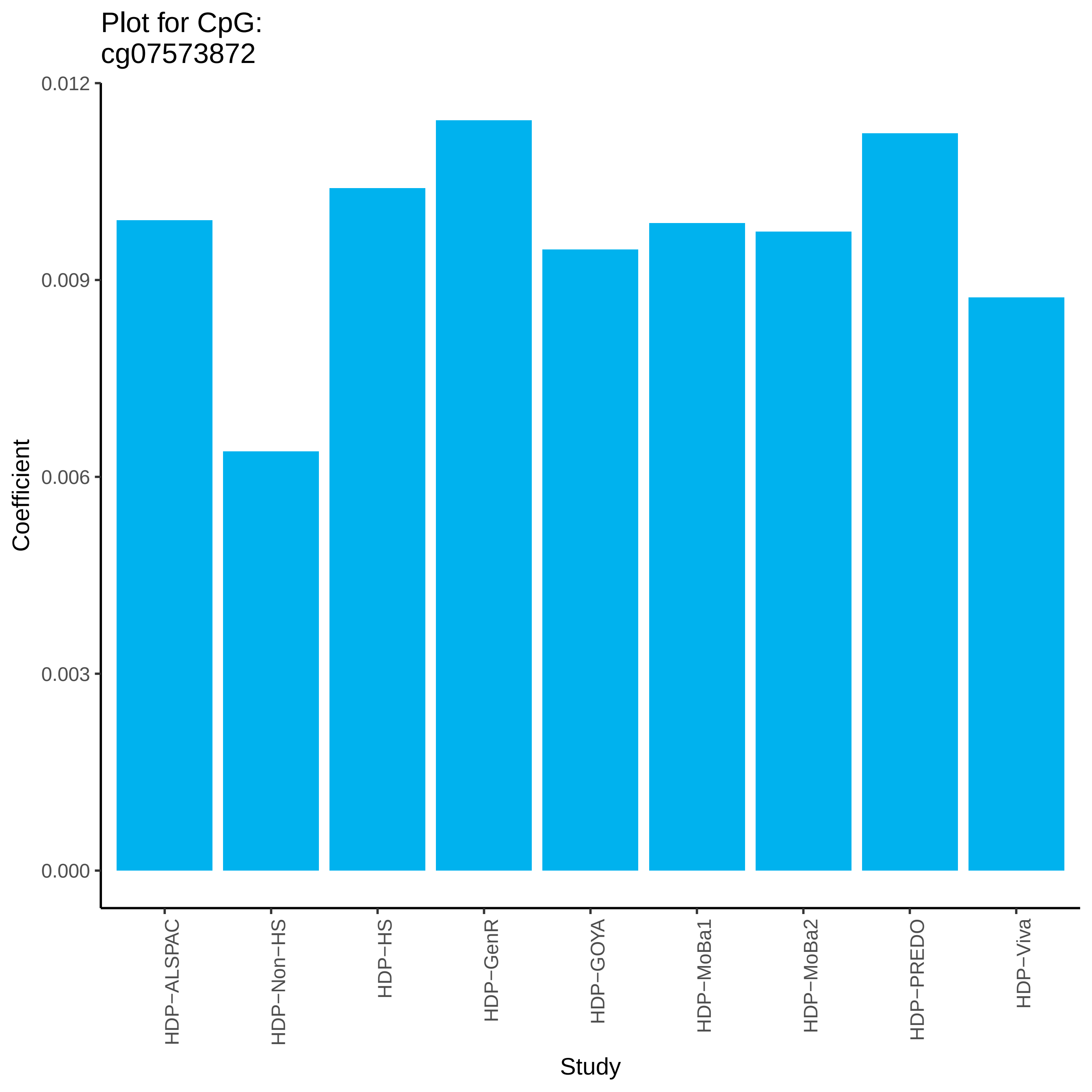
Plot for CpG:
cg07573872
0.012
0.009
0.006
0.003
0.000
Coefficient
HDP−ALSPAC
HDP−Non−HS
HDP−HS
HDP−GenR
HDP−GOYA
HDP−MoBa1
HDP−MoBa2
HDP−PREDO
HDP−Viva
Study

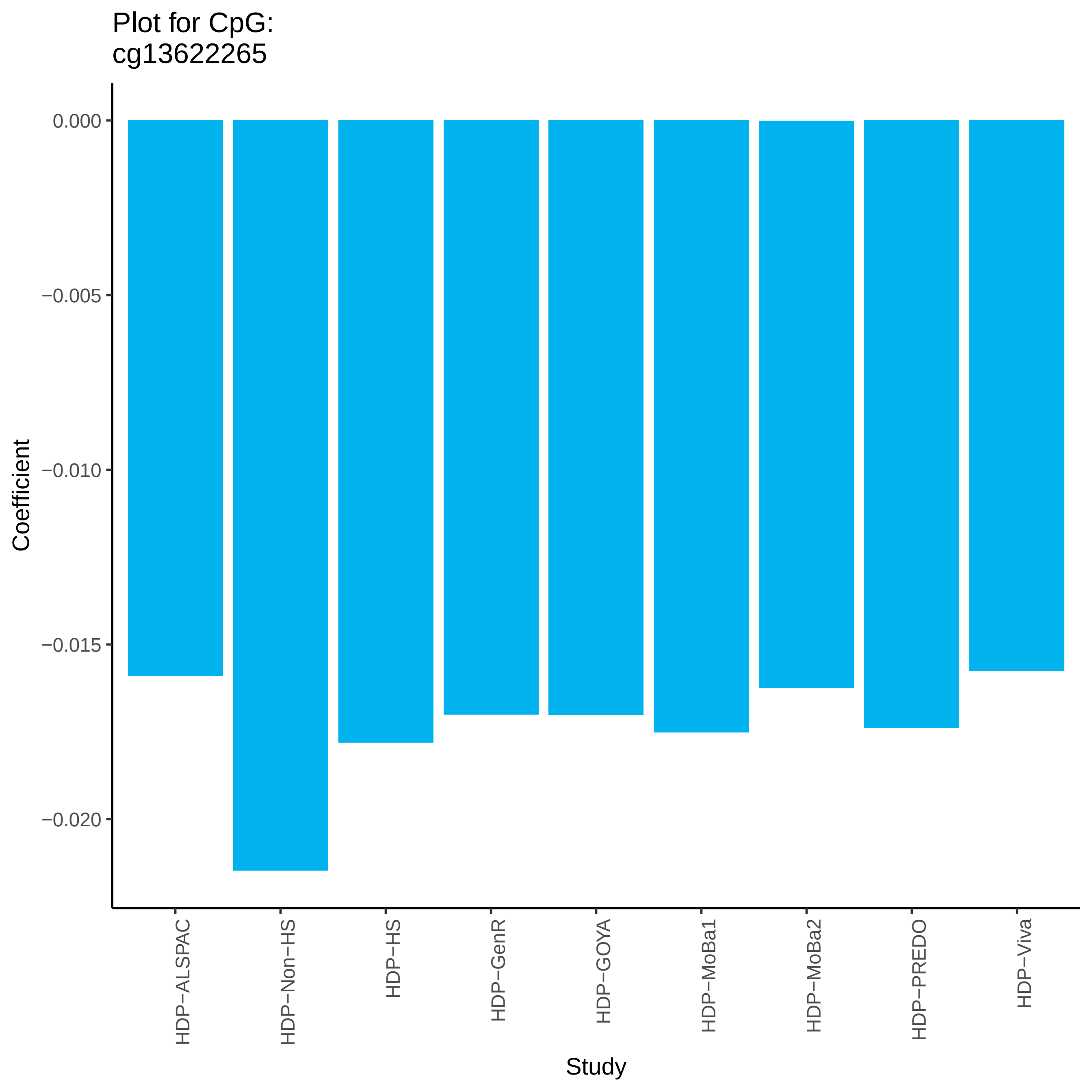

Plot for CpG:
cg13622265
Coefficient
0.000
−0.005
−0.010
−0.015
−0.020
HDP−ALSPAC
HDP−Non−HS
HDP−HS
HDP−GenR
HDP−GOYA
HDP−MoBa1
HDP−MoBa2
HDP−PREDO
HDP−Viva
Study

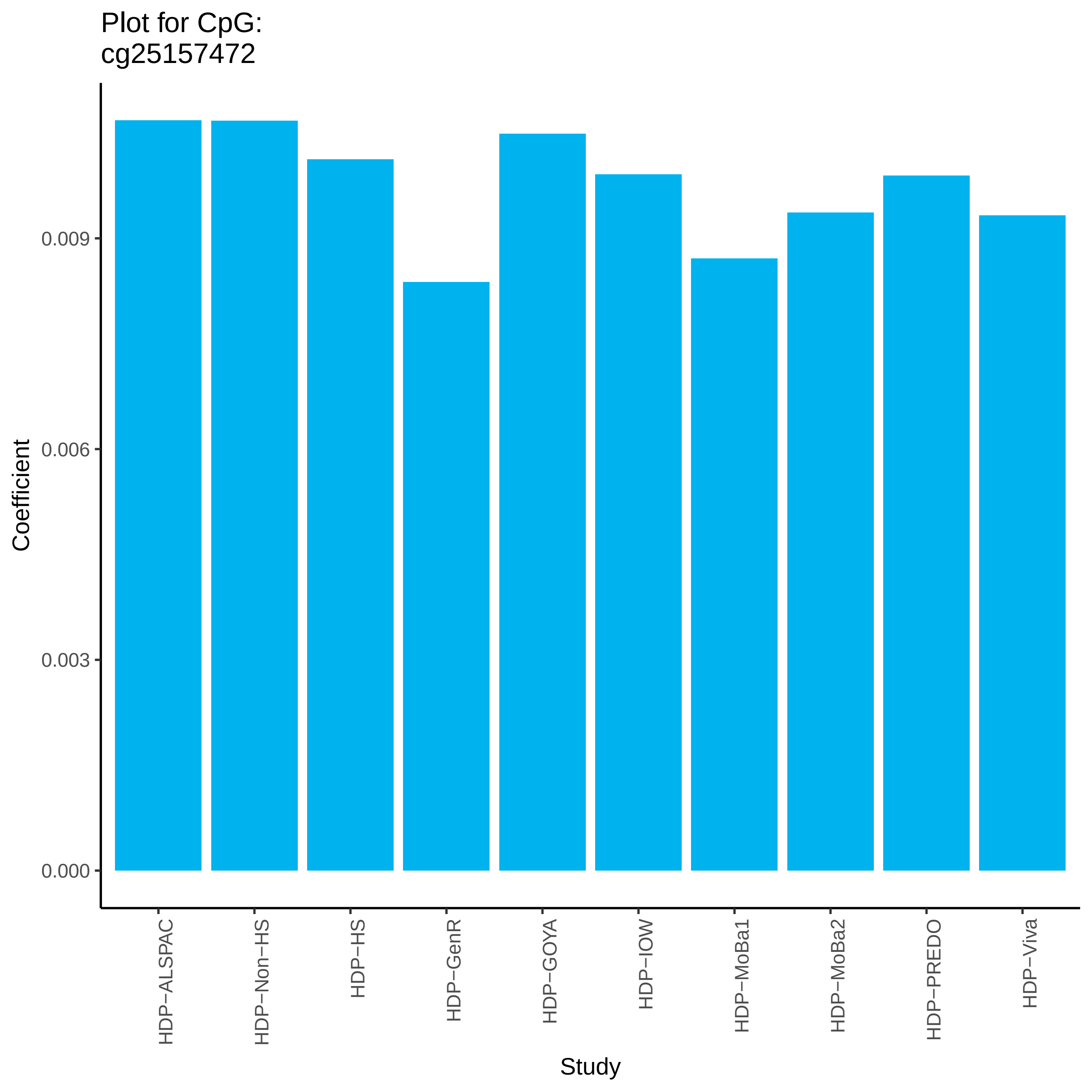
Plot for CpG:
cg25157472
Coefficient
0.009
0.006
0.003
0.000
HDP−ALSPAC
HDP−Non−HS
HDP−HS
HDP−GenR
HDP−GOYA
HDP−IOW
HDP−MoBa1
HDP−MoBa2
HDP−PREDO
HDP−Viva
Study

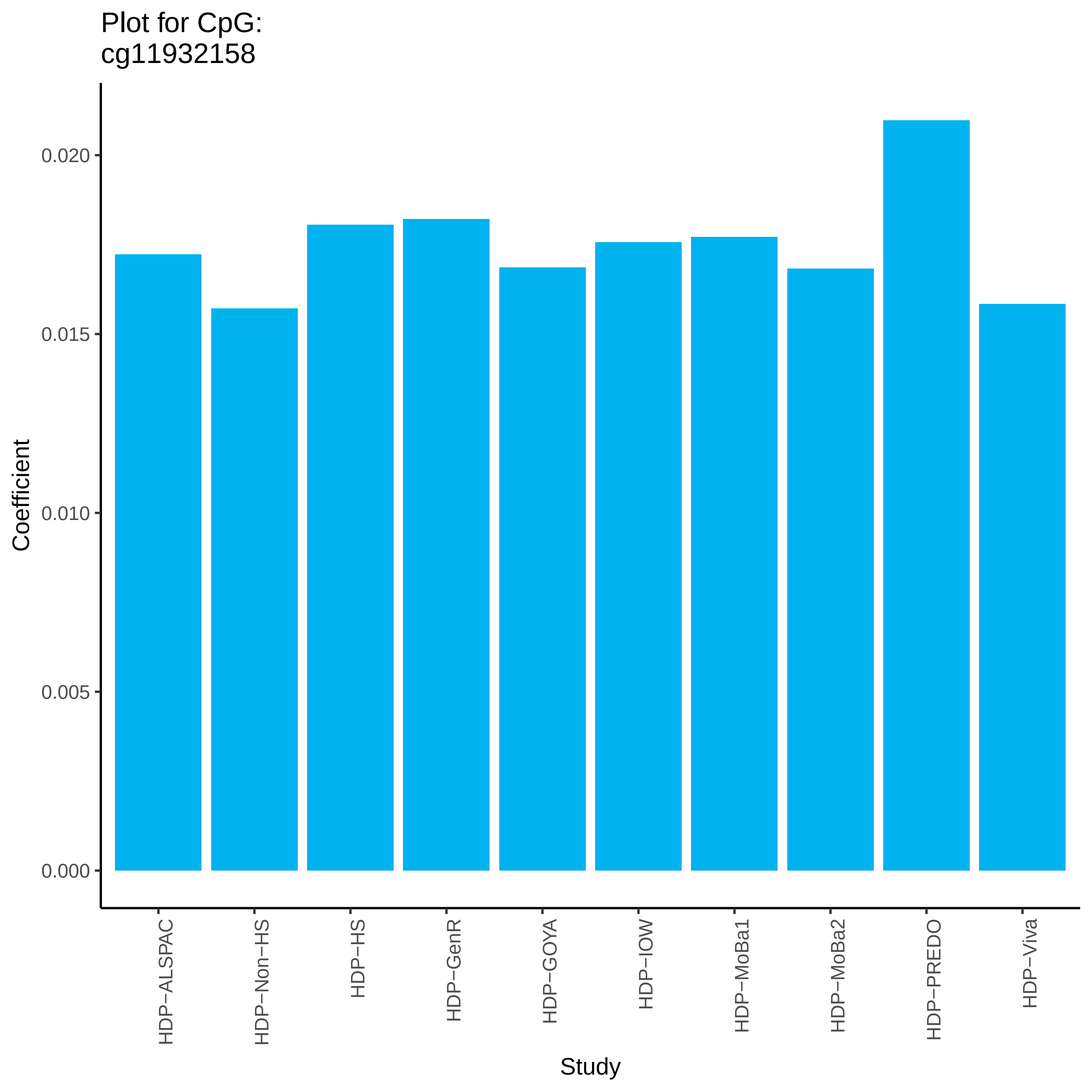

Plot for CpG:
cg11932158
Coefficient
0.000
0.005
0.010
0.015
0.020
Study
HDP−ALSPAC
HDP−Non−HS
HDP−HS
HDP−GenR
HDP−GOYA
HDP−IOW
HDP−MoBa1
HDP−MoBa2
HDP−PREDO
HDP−Viva

Plot for CpG:
cg02430430

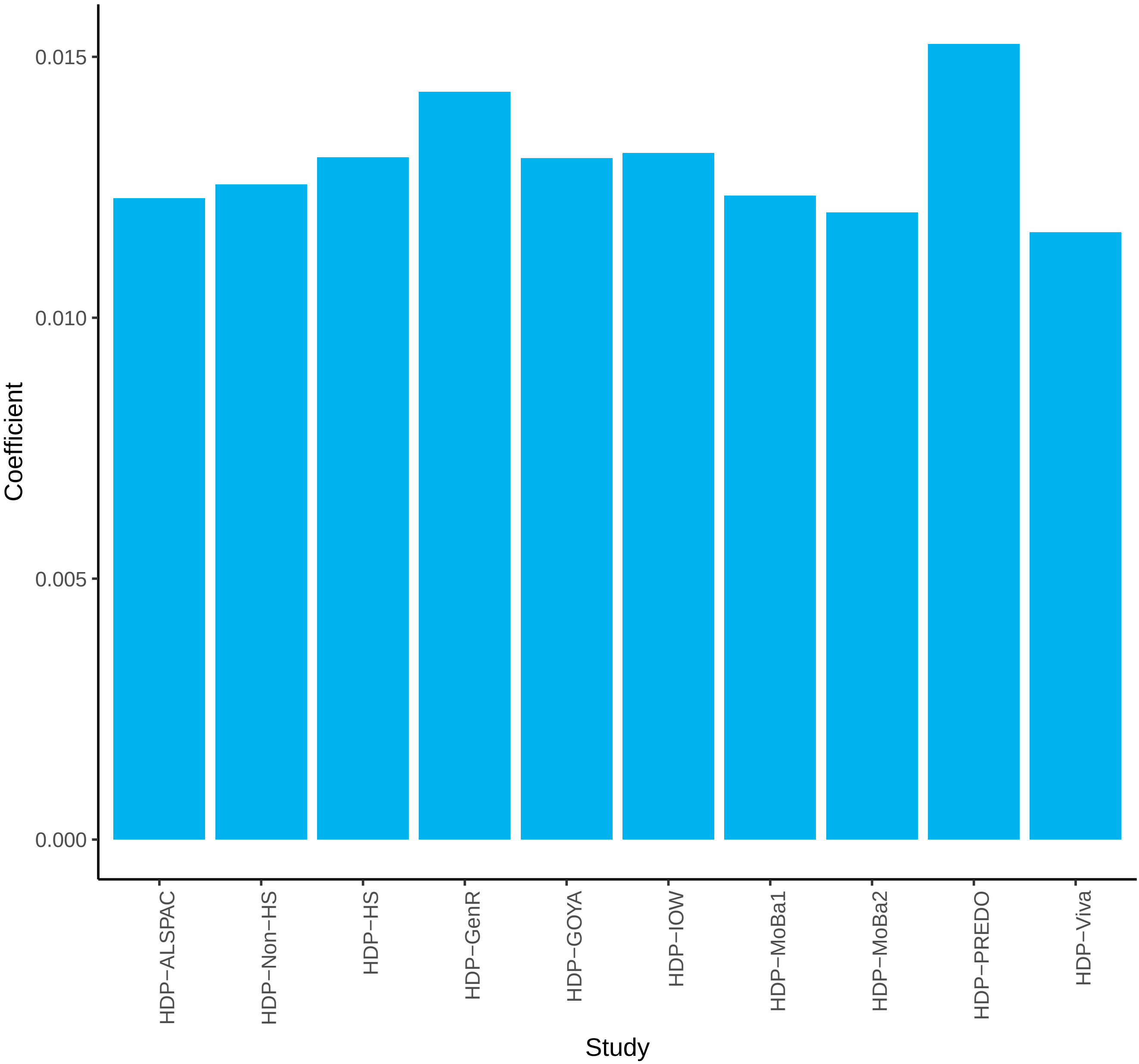

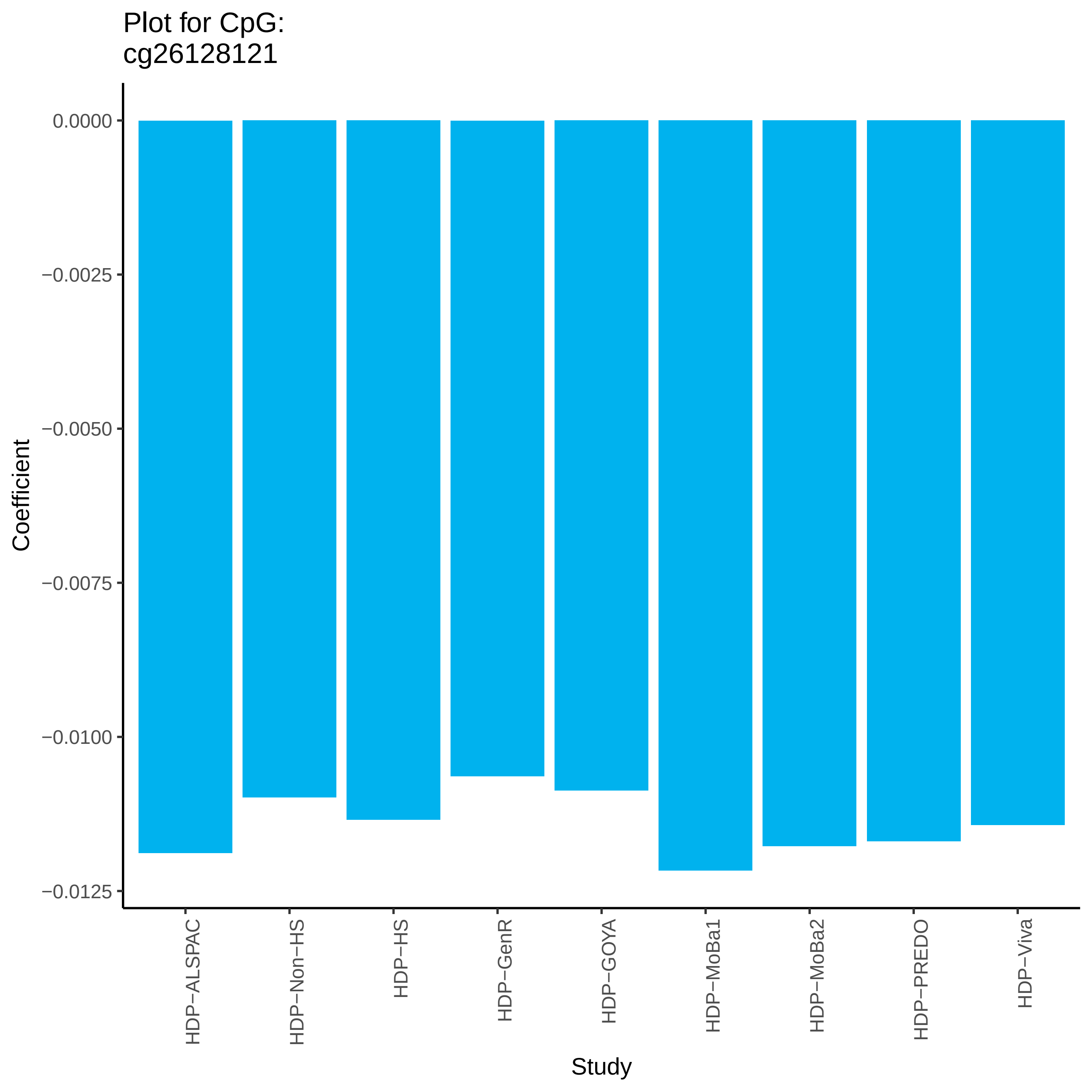
Plot for CpG:
cg26128121
Coefficient
0.0000
−0.0025
−0.0050
−0.0075
−0.0100
−0.0125
HDP−ALSPAC
HDP−Non−HS
HDP−HS
HDP−GenR
HDP−GOYA
HDP−MoBa1
HDP−MoBa2
HDP−PREDO
HDP−Viva
Study

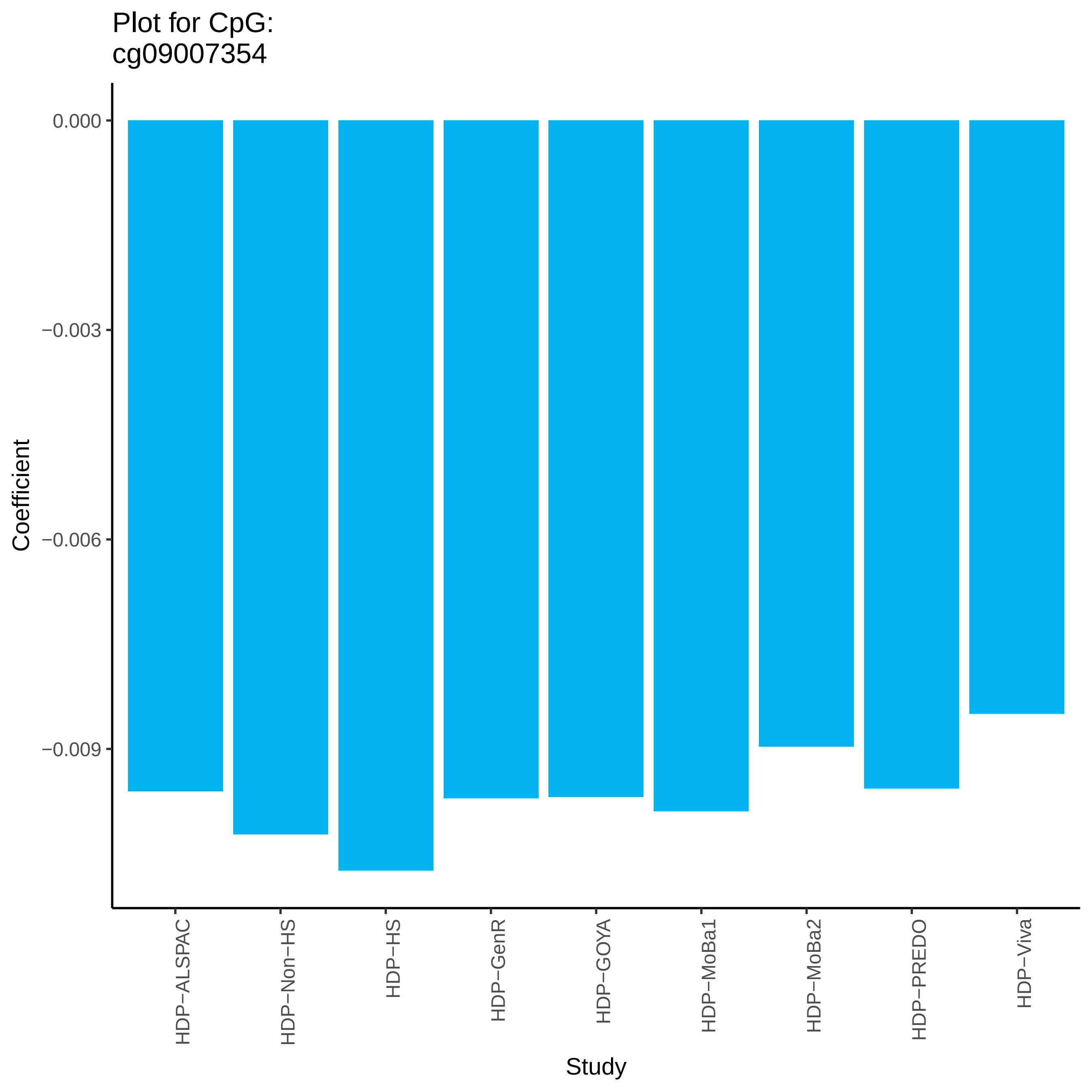

Plot for CpG:
cg09007354
Coefficient
0.000
−0.003
−0.006
−0.009
Study
HDP−ALSPAC
HDP−Non−HS
HDP−HS
HDP−GenR
HDP−GOYA
HDP−MoBa1
HDP−MoBa2
HDP−PREDO
HDP−Viva

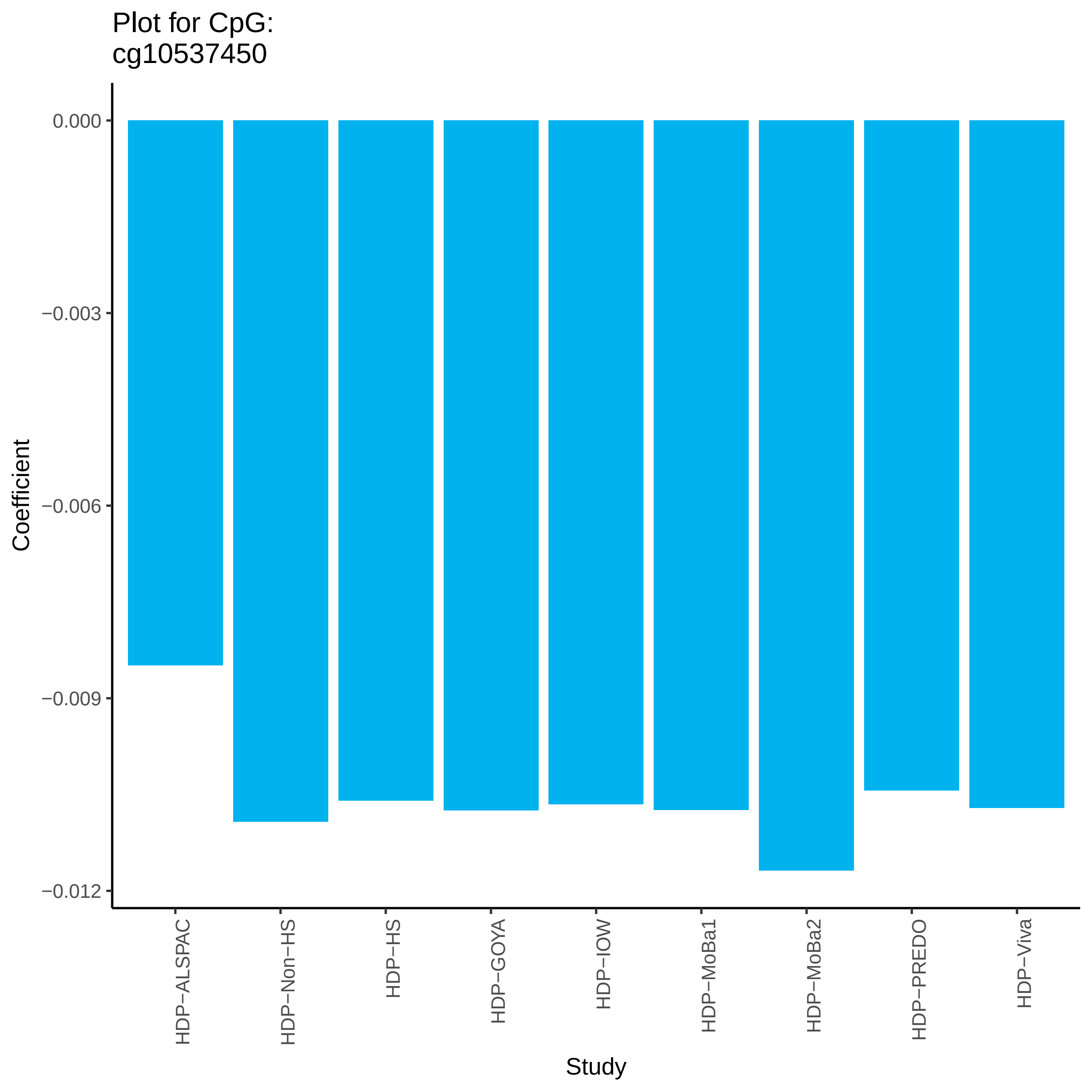

Plot for CpG:
cg10537450
0.000
−0.003
−0.006
−0.009
−0.012
Coefficient
HDP−ALSPAC
HDP−Non−HS
HDP−HS
HDP−GOYA
HDP−IOW
HDP−MoBa1
HDP−MoBa2
HDP−PREDO
HDP−Viva
Study

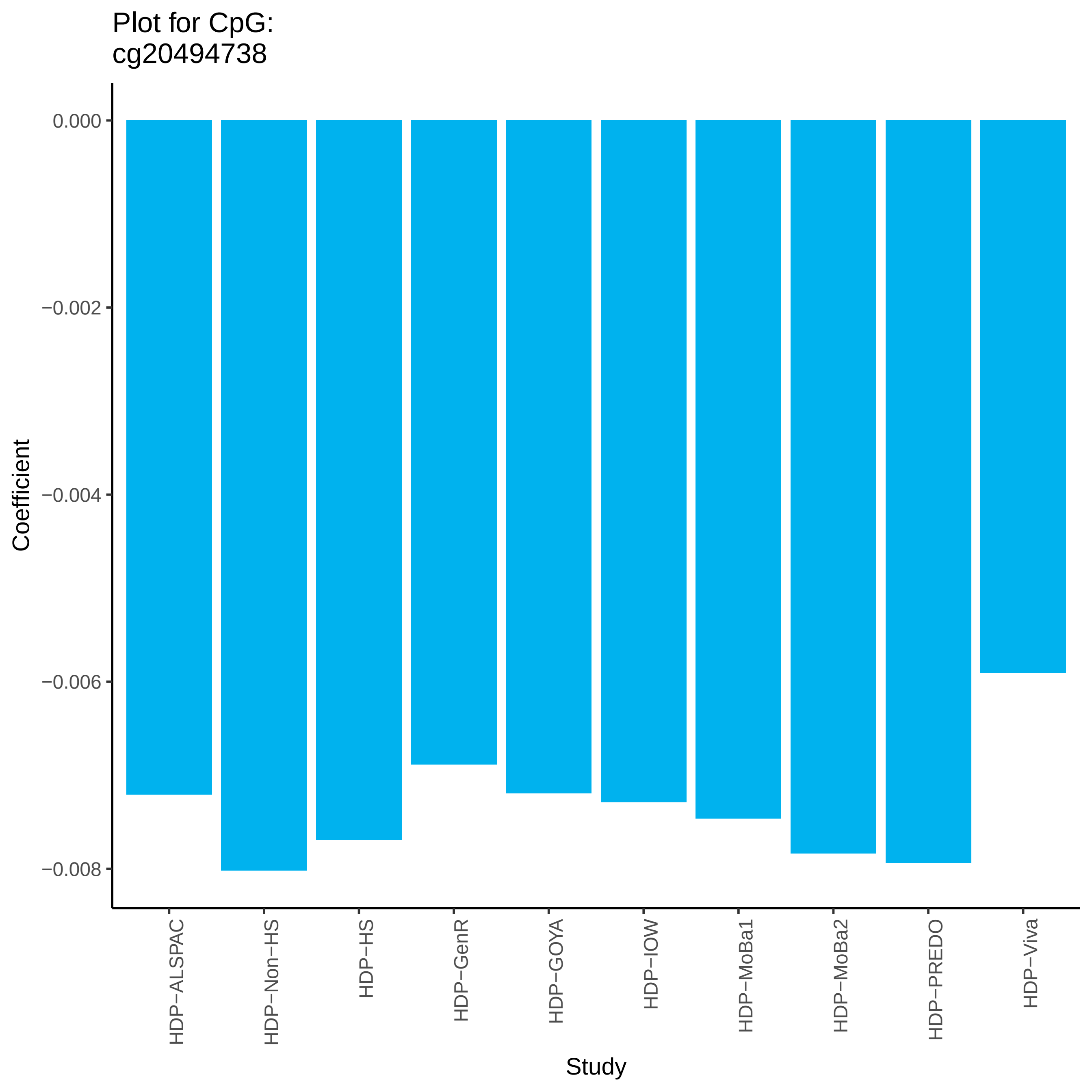
Plot for CpG:
cg20494738
Coefficient
0.000
−0.002
−0.004
−0.006
−0.008
HDP−ALSPAC
HDP−Non−HS
HDP−HS
HDP−GenR
HDP−GOYA
HDP−IOW
HDP−MoBa1
HDP−MoBa2
HDP−PREDO
HDP−Viva
Study

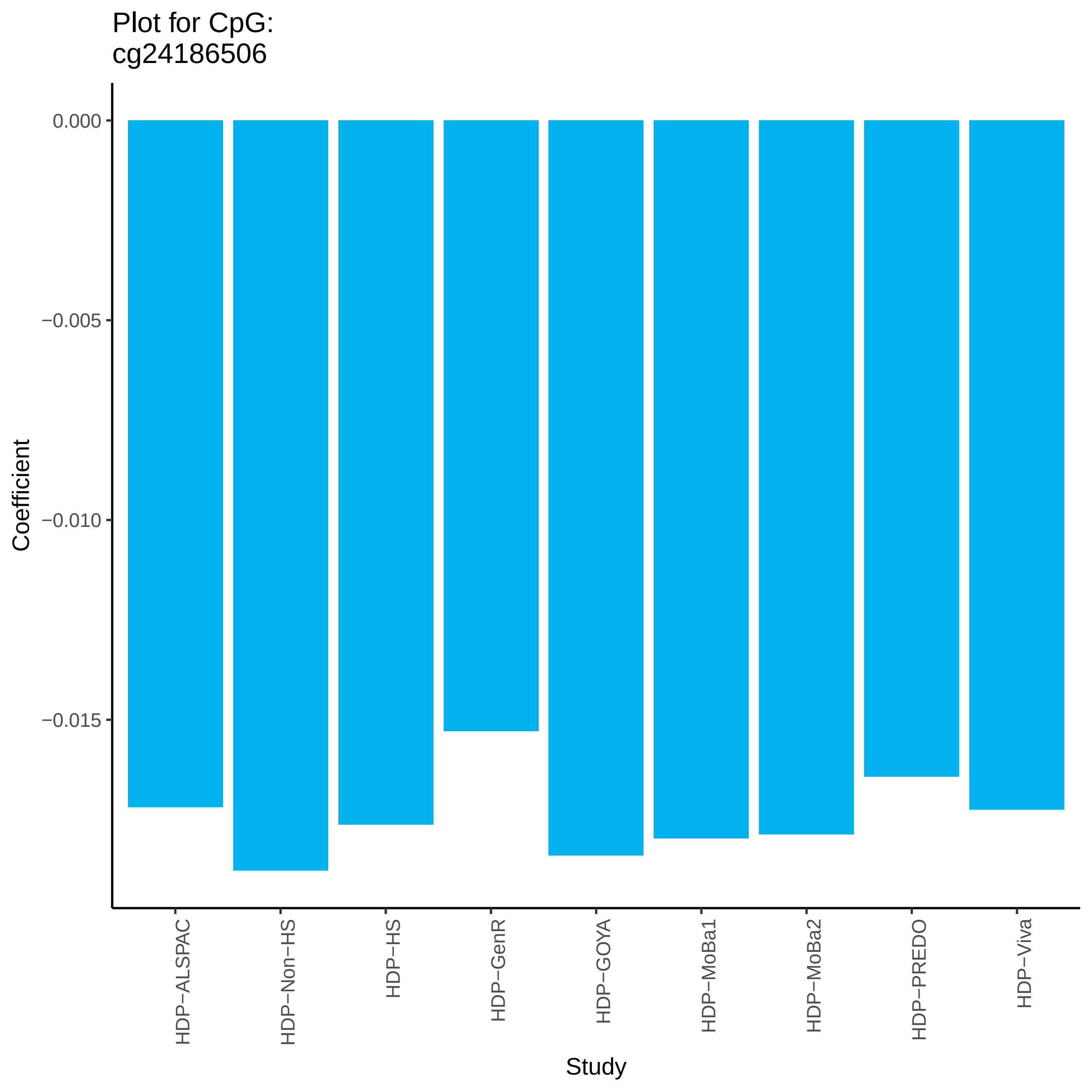

Plot for CpG:
cg24186506
0.000
−0.005
−0.010
−0.015
Coefficient
HDP−ALSPAC
HDP−Non−HS
HDP−HS
HDP−GenR
HDP−GOYA
HDP−MoBa1
HDP−MoBa2
HDP−PREDO
HDP−Viva
Study

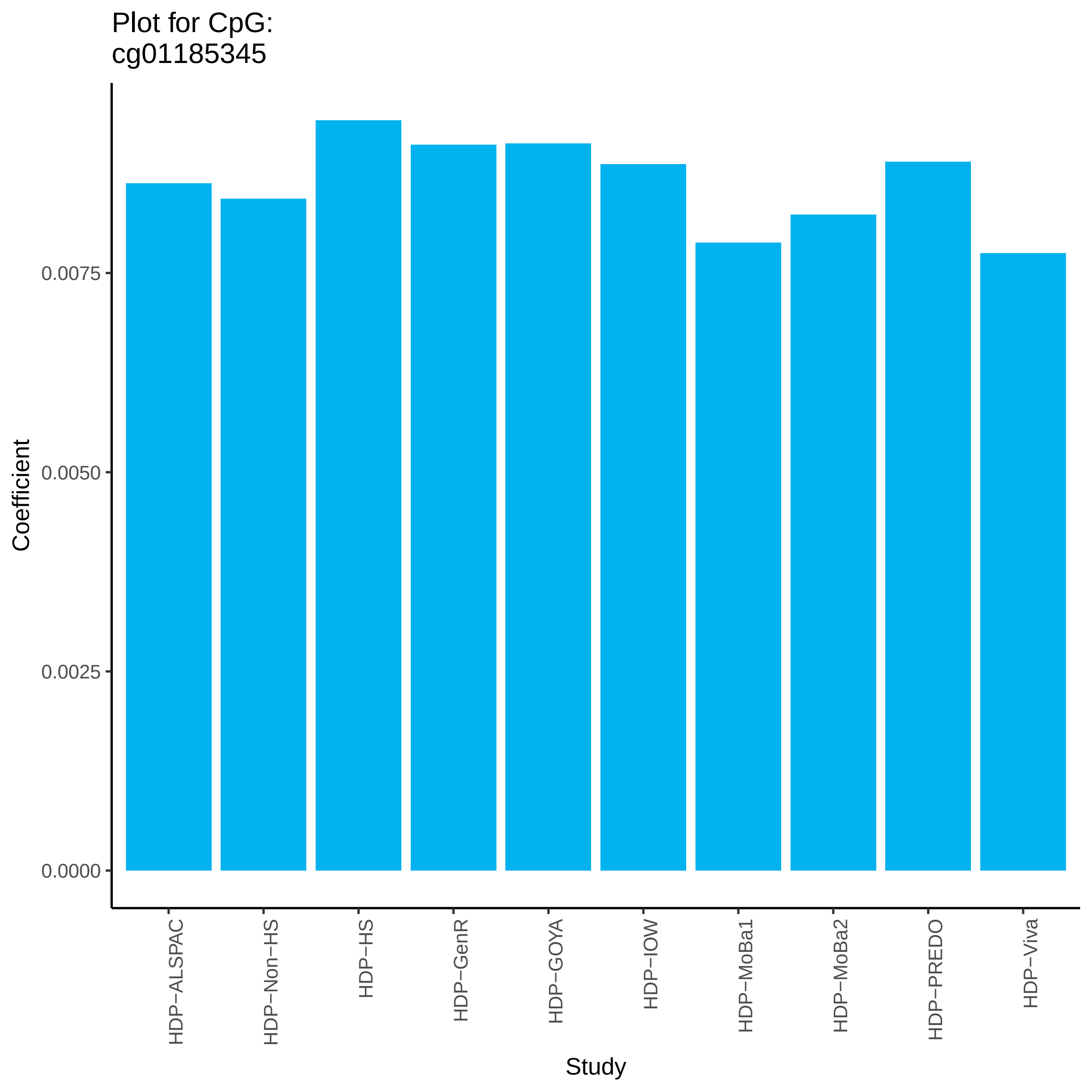

Plot for CpG:
cg01185345
Coefficient
0.0075
0.0050
0.0025
0.0000
HDP−ALSPAC
HDP−Non−HS
HDP−HS
HDP−GenR
HDP−GOYA
HDP−IOW
HDP−MoBa1
HDP−MoBa2
HDP−PREDO
HDP−Viva
Study

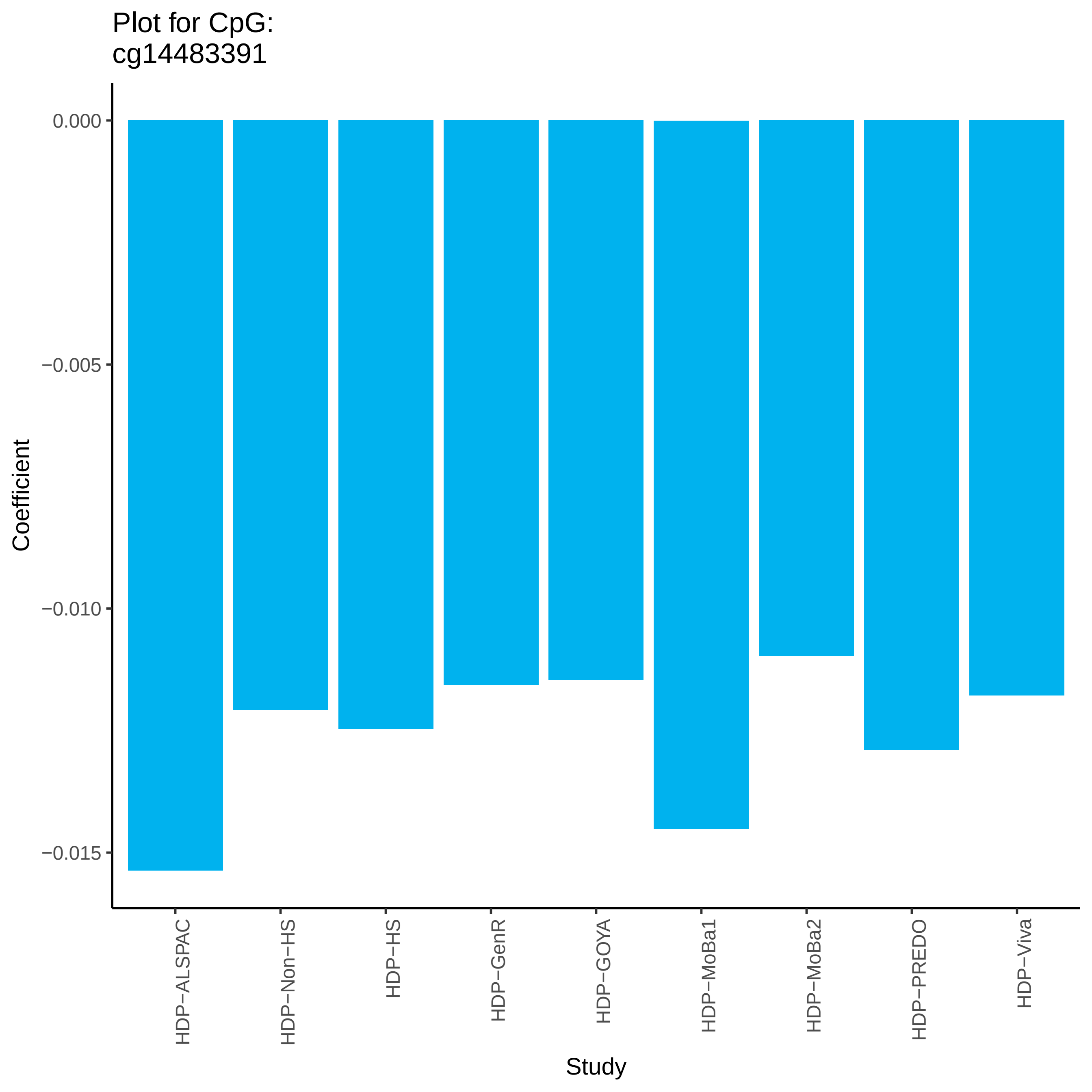
Plot for CpG:
cg14483391
0.000
−0.005
−0.010
−0.015
Coefficient
HDP−ALSPAC
HDP−Non−HS
HDP−HS
HDP−GenR
HDP−GOYA
HDP−MoBa1
HDP−MoBa2
HDP−PREDO
HDP−Viva
Study

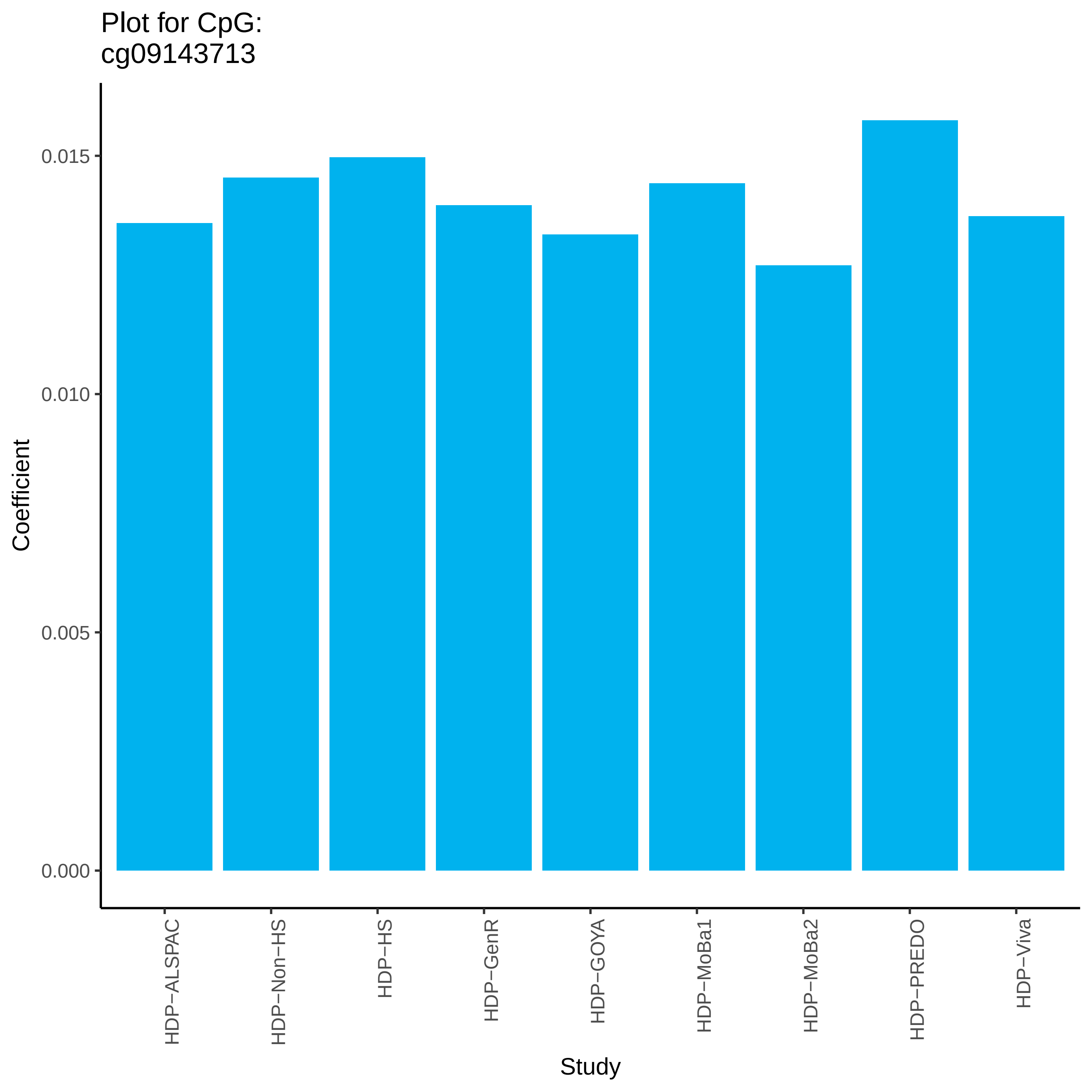

Plot for CpG:
cg09143713
Coefficient
0.015
0.010
0.005
0.000
HDP−ALSPAC
HDP−Non−HS
HDP−HS
HDP−GenR
HDP−GOYA
HDP−MoBa1
HDP−MoBa2
HDP−PREDO
HDP−Viva
Study

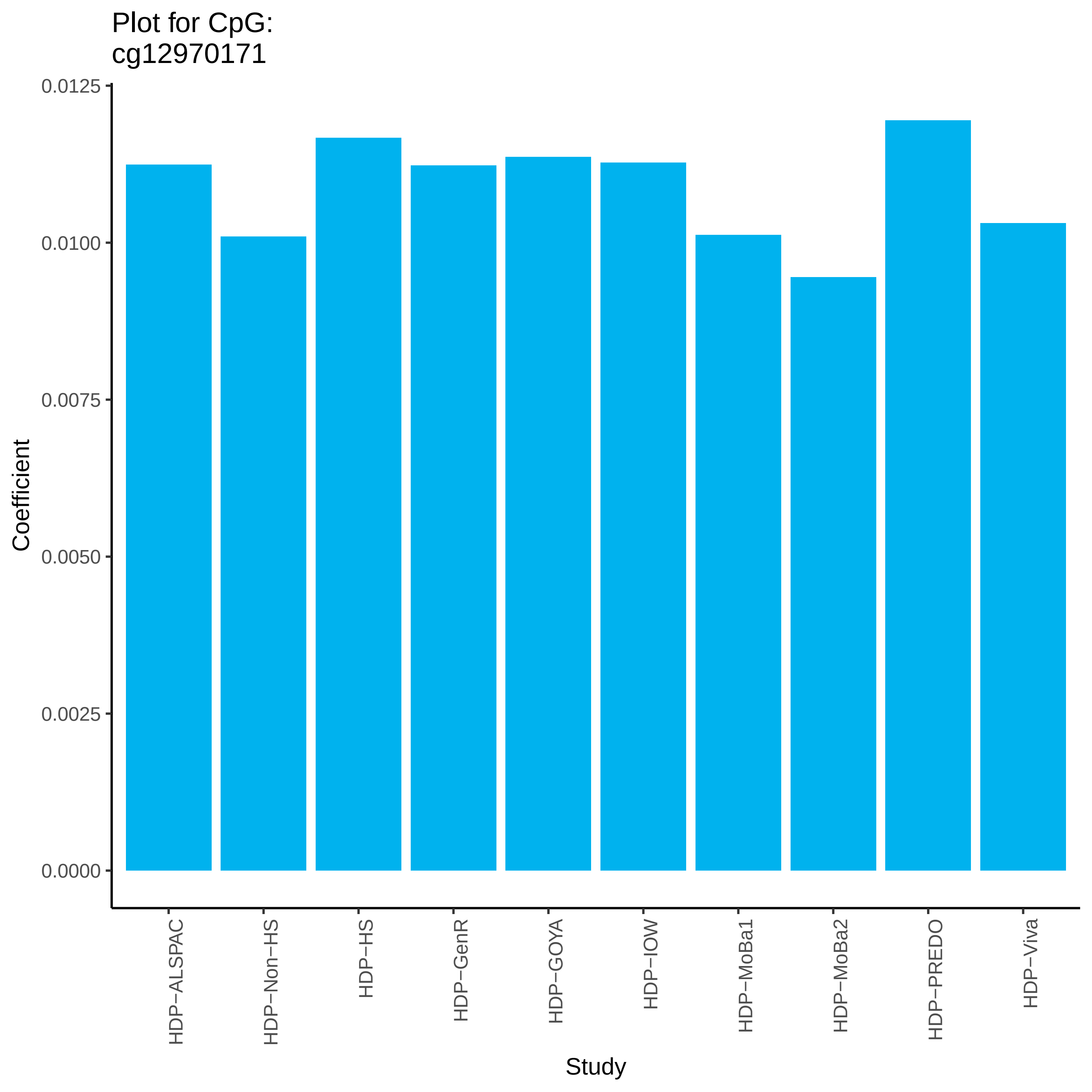
Plot for CpG:
cg12970171
Coefficient
0.0125
0.0100
0.0075
0.0050
0.0025
0.0000
HDP−ALSPAC
HDP−Non−HS
HDP−HS
HDP−GenR
HDP−GOYA
HDP−IOW
HDP−MoBa1
HDP−MoBa2
HDP−PREDO
HDP−Viva
Study

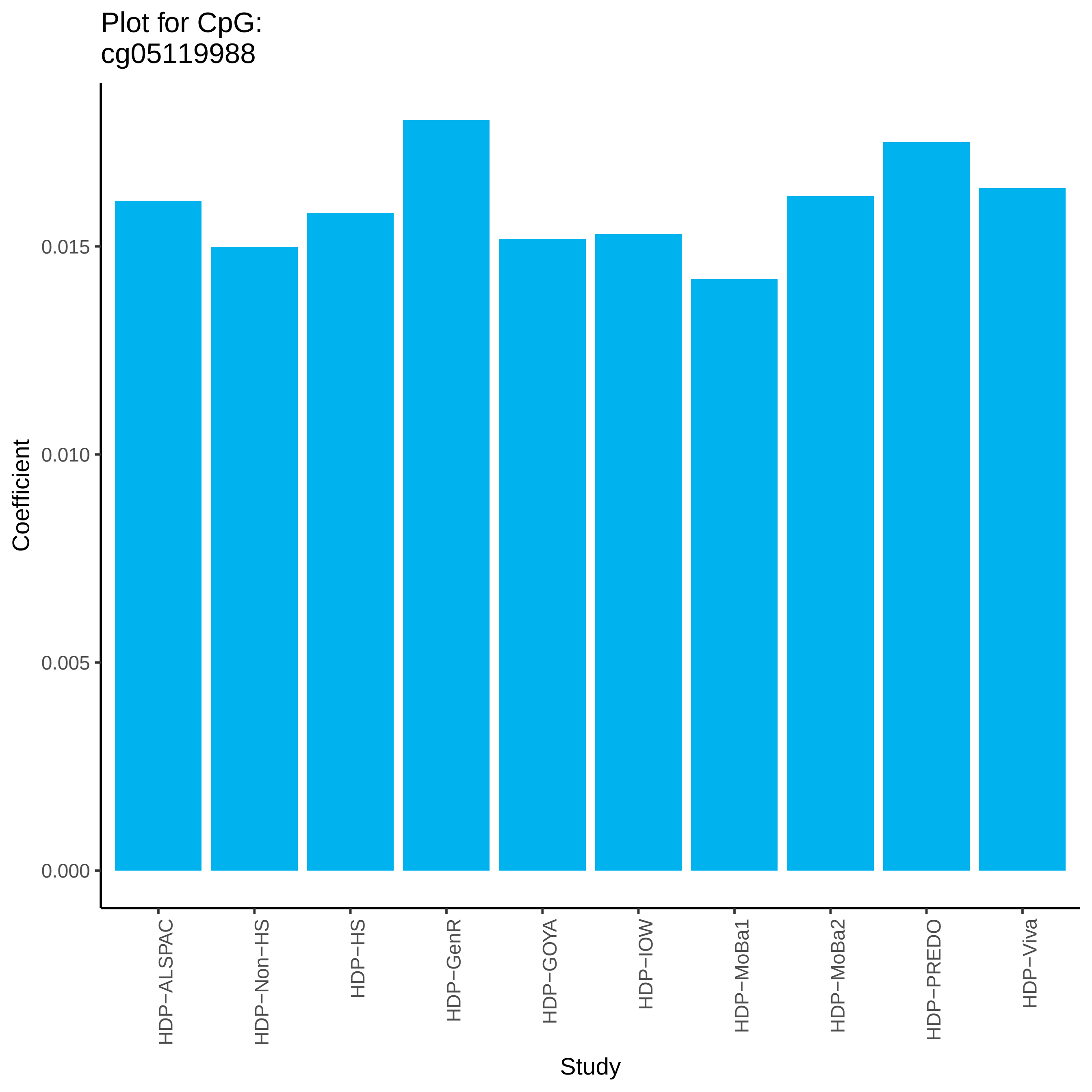

Plot for CpG:
cg05119988
Coefficient
0.015
0.010
0.005
0.000
HDP−ALSPAC
HDP−Non−HS
HDP−HS
HDP−GenR
HDP−GOYA
HDP−IOW
HDP−MoBa1
HDP−MoBa2
HDP−PREDO
HDP−Viva
Study

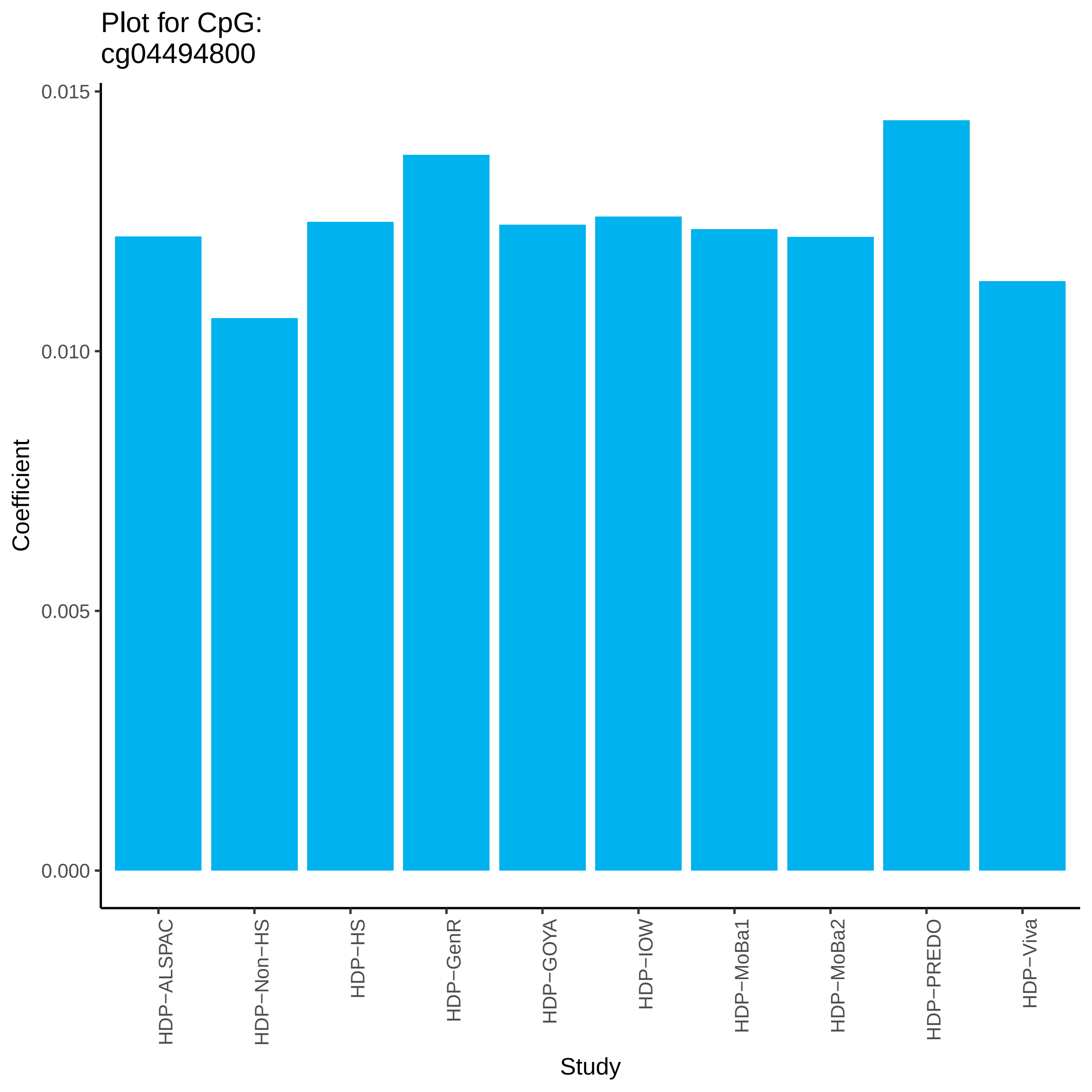
Plot for CpG:
cg04494800
0.015
0.010
0.005
0.000
Coefficient
HDP−ALSPAC
HDP−Non−HS
HDP−HS
HDP−GenR
HDP−GOYA
HDP−IOW
HDP−MoBa1
HDP−MoBa2
HDP−PREDO
HDP−Viva
Study

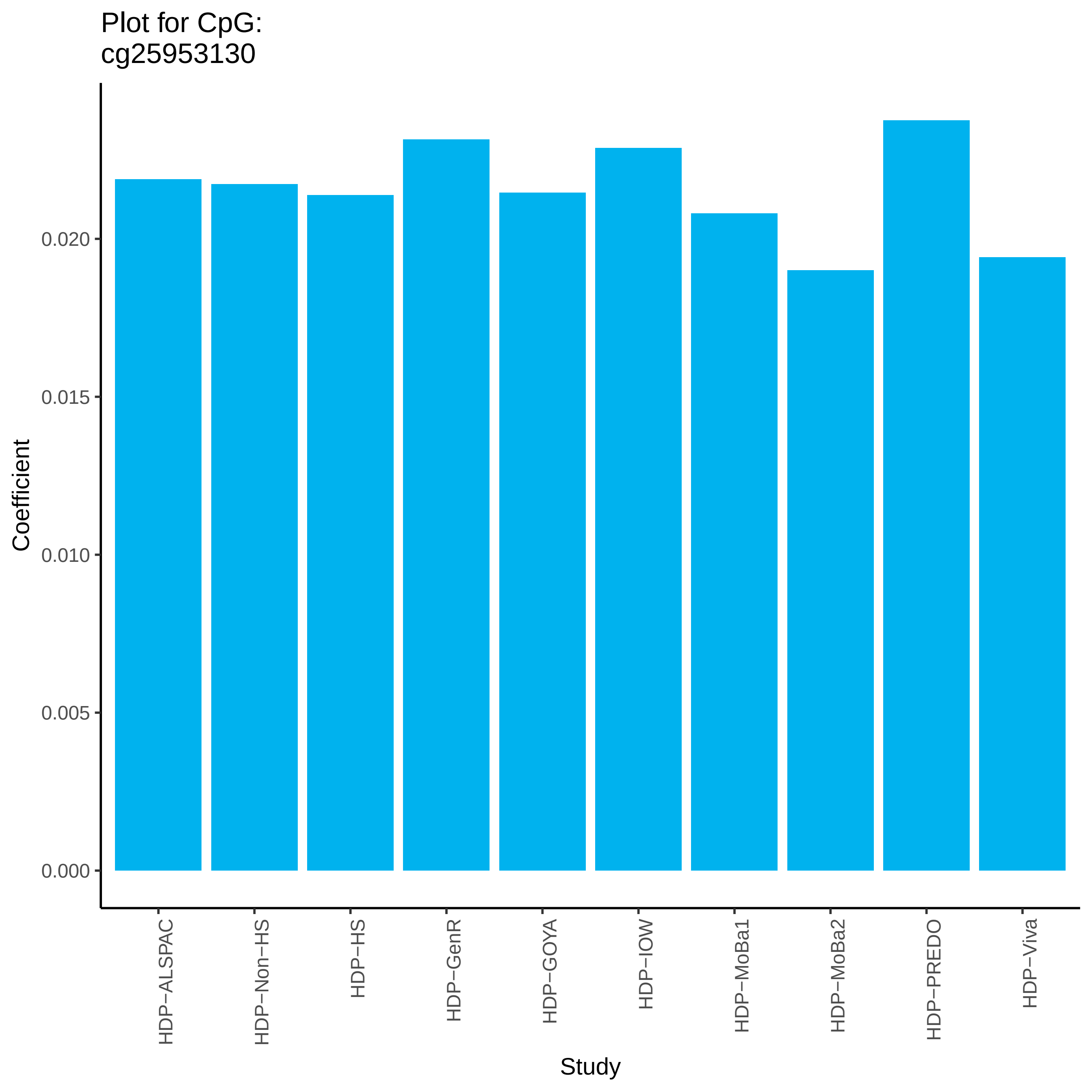

Plot for CpG:
cg25953130
Coefficient
0.000
0.005
0.010
0.015
0.020
Study
HDP−ALSPAC
HDP−Non−HS
HDP−HS
HDP−GenR
HDP−GOYA
HDP−IOW
HDP−MoBa1
HDP−MoBa2
HDP−PREDO
HDP−Viva

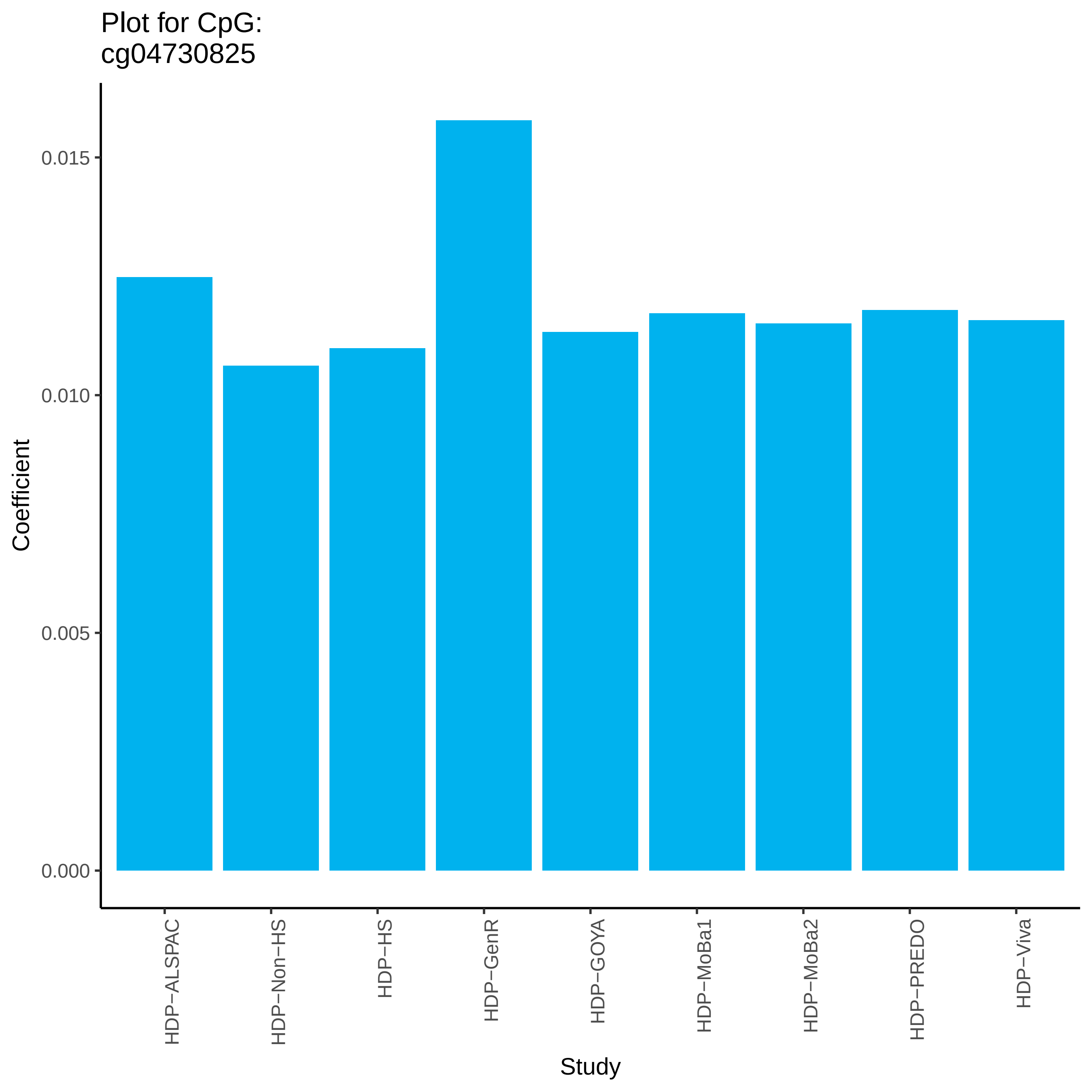
Plot for CpG:
cg04730825
Coefficient
0.015
0.010
0.005
0.000
HDP−ALSPAC
HDP−Non−HS
HDP−HS
HDP−GenR
HDP−GOYA
HDP−MoBa1
HDP−MoBa2
HDP−PREDO
HDP−Viva
Study

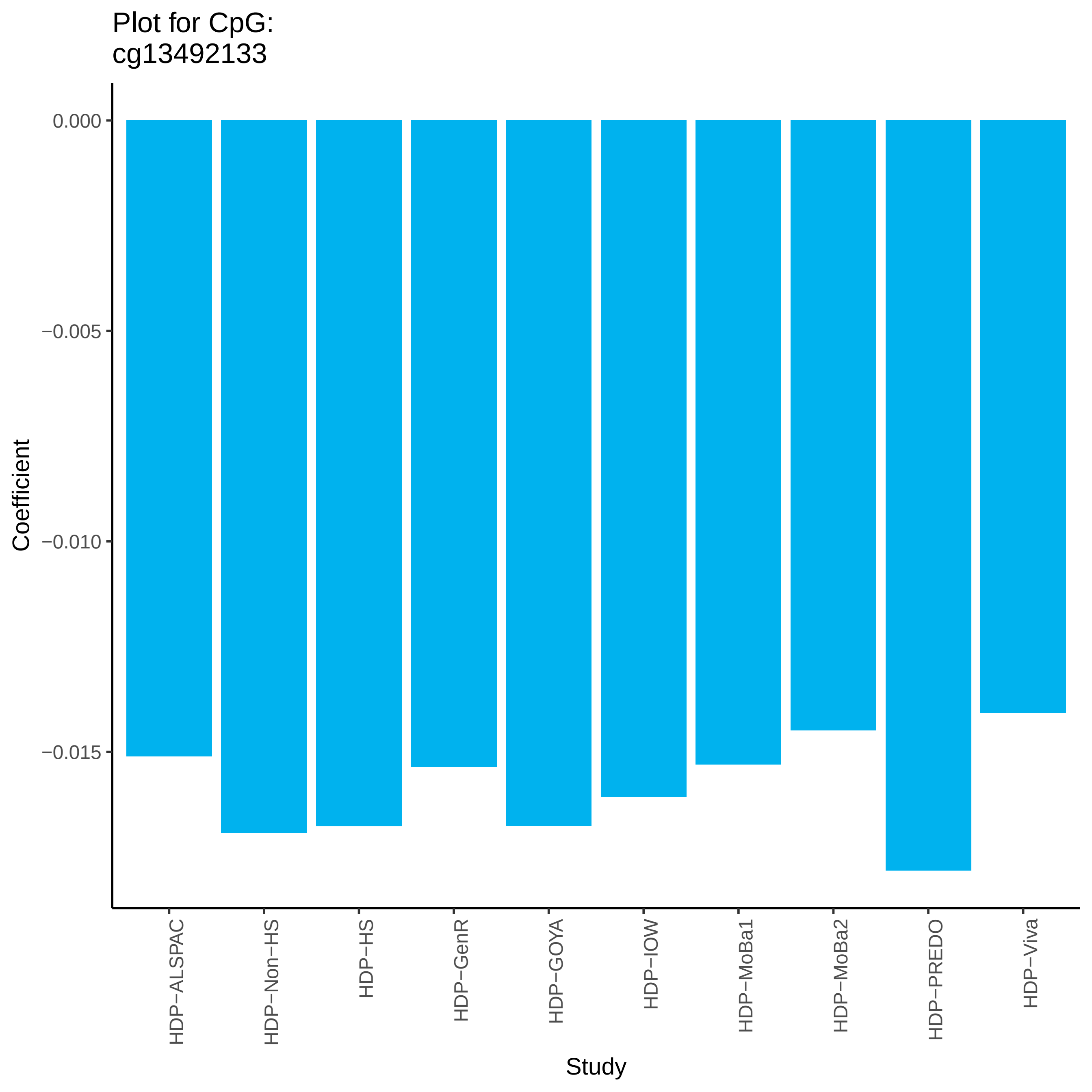

Plot for CpG:
cg13492133
0.000
−0.005
−0.010
−0.015
Coefficient
HDP−ALSPAC
HDP−Non−HS
HDP−HS
HDP−GenR
HDP−GOYA
HDP−IOW
HDP−MoBa1
HDP−MoBa2
HDP−PREDO
HDP−Viva
Study

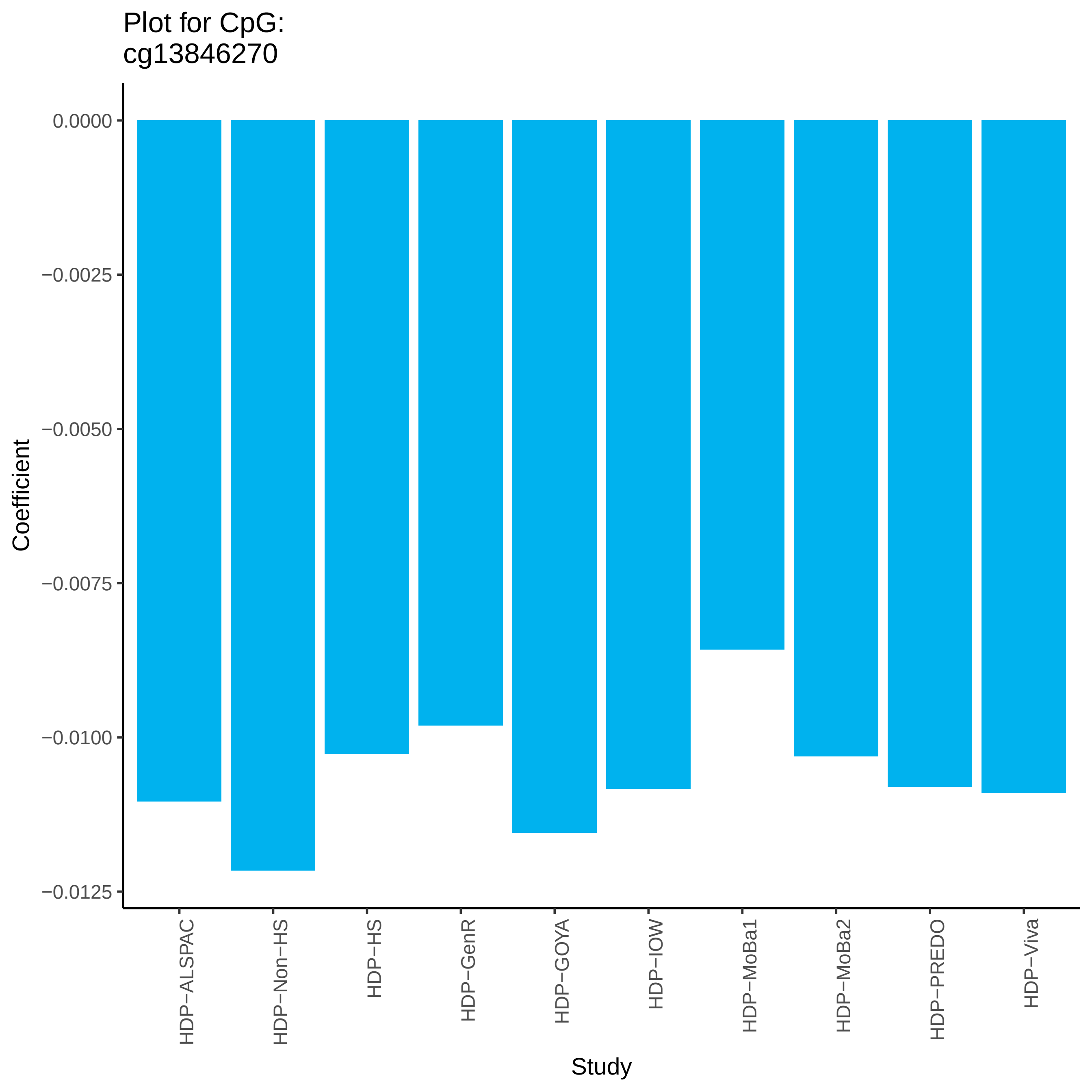

Plot for CpG:
cg13846270
Coefficient
0.0000
−0.0025
−0.0050
−0.0075
−0.0100
−0.0125
HDP−ALSPAC
HDP−Non−HS
HDP−HS
HDP−GenR
HDP−GOYA
HDP−IOW
HDP−MoBa1
HDP−MoBa2
HDP−PREDO
HDP−Viva
Study

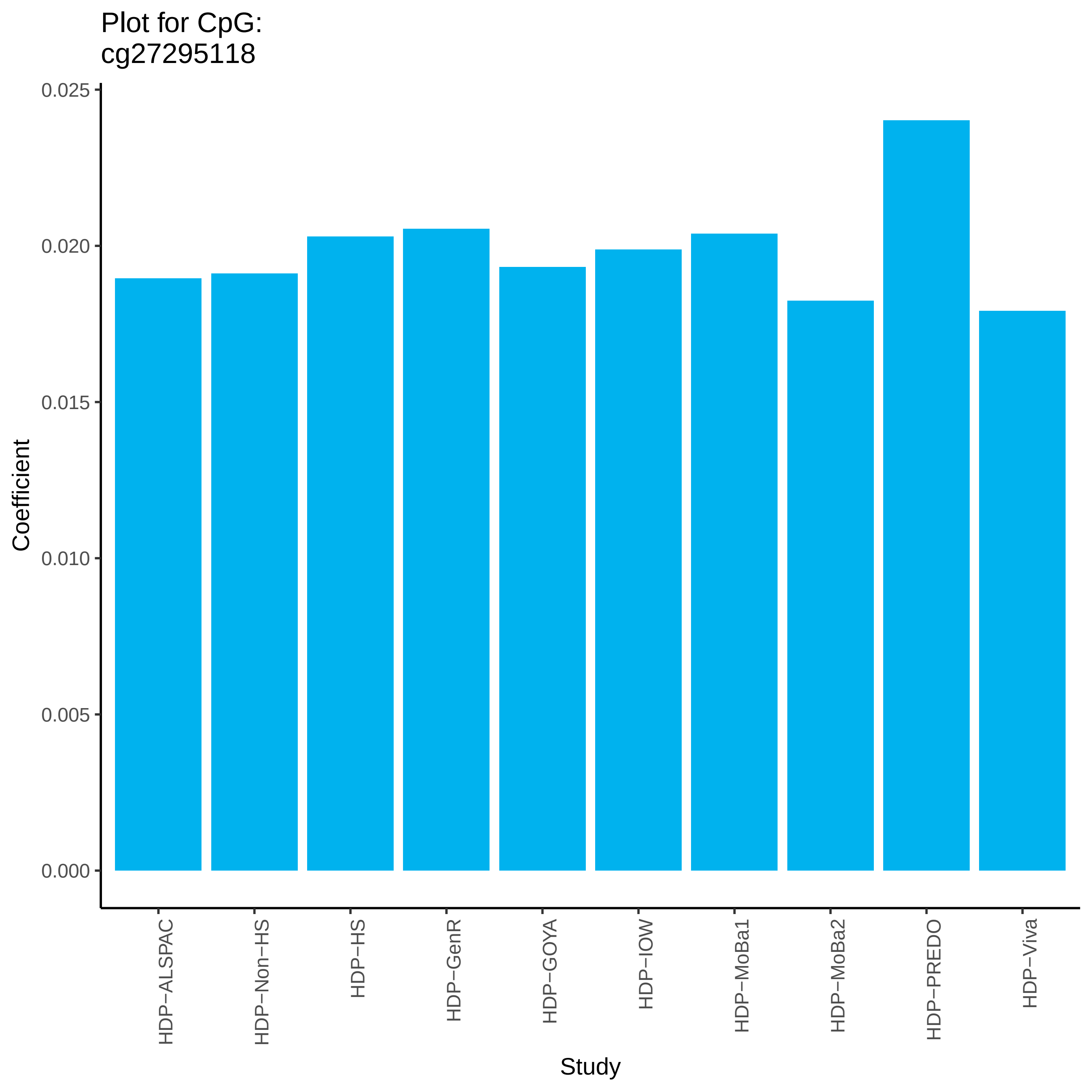
Plot for CpG:
cg27295118
Coefficient
0.025
0.020
0.015
0.010
0.005
0.000
HDP−ALSPAC
HDP−Non−HS
HDP−HS
HDP−GenR
HDP−GOYA
HDP−IOW
HDP−MoBa1
HDP−MoBa2
HDP−PREDO
HDP−Viva
Study

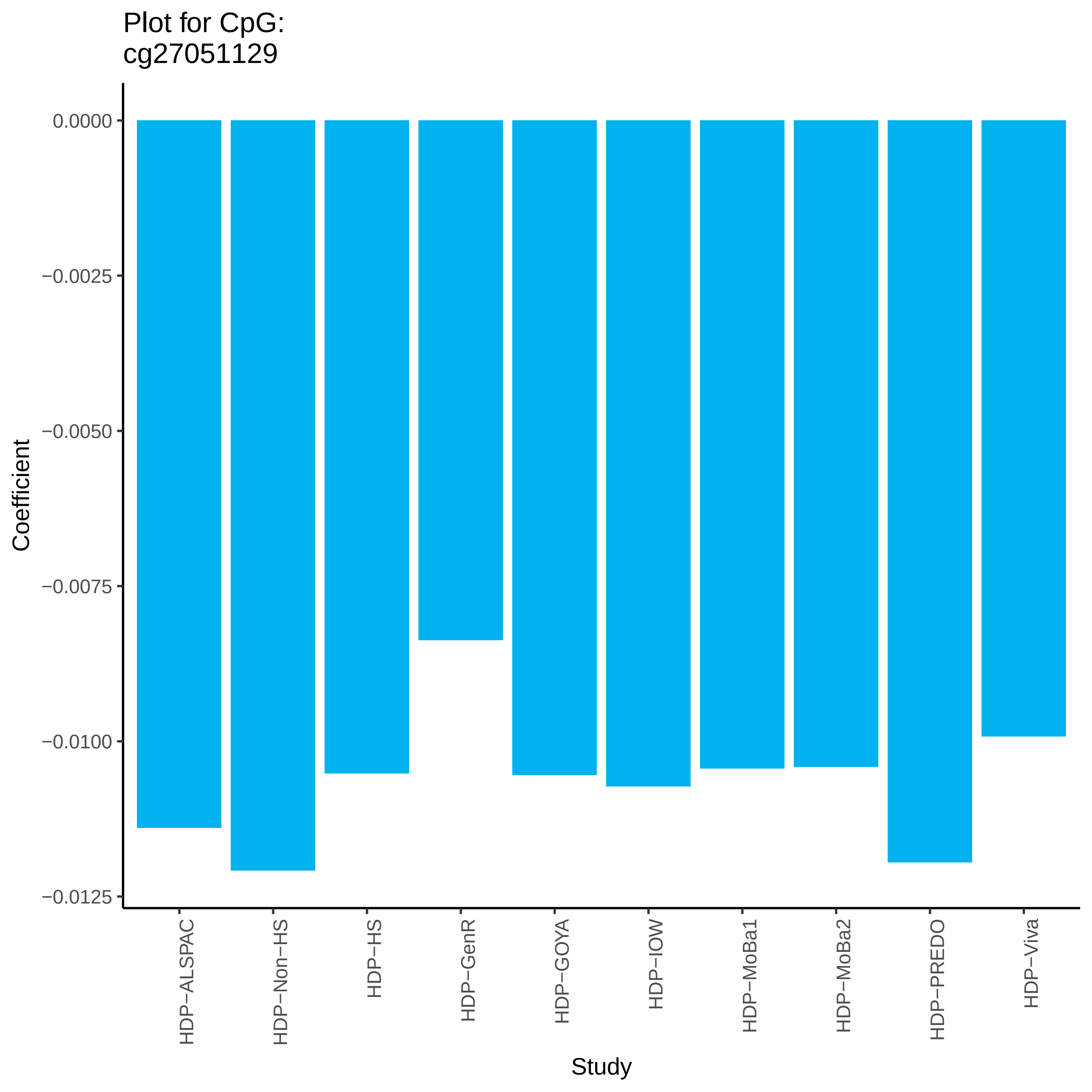
Plot for CpG:
cg27051129
0.0000
−0.0025
−0.0050
−0.0075
−0.0100
−0.0125
Coefficient
HDP−ALSPAC
HDP−Non−HS
HDP−HS
HDP−GenR
HDP−GOYA
HDP−IOW
HDP−MoBa1
HDP−MoBa2
HDP−PREDO
HDP−Viva
Study
