# Supplementary material for: Hypertensive Disorders of Pregnancy and DNA Methylation in Newborns: Findings From the Pregnancy and Childhood Epigenetics Consortium
Source: Hypertension. 2019 Jun 24;74(2):375–83. doi: 10.1161/HYPERTENSIONAHA.119.12634 (PMC6635125; doi:10.1161/HYPERTENSIONAHA.119.12634)

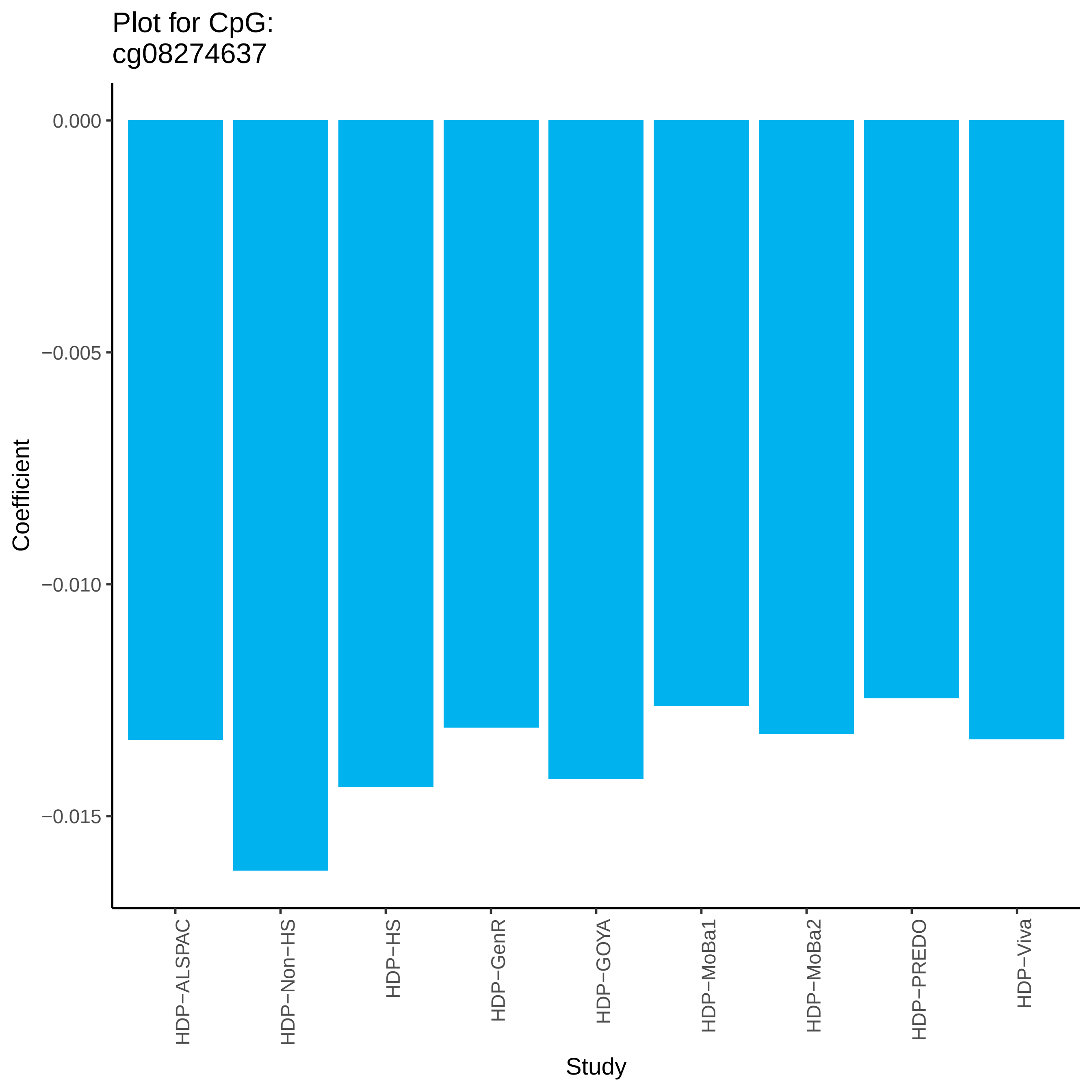

Plot for CpG:
cg08274637
0.000
−0.005
−0.010
−0.015
Coefficient
HDP−ALSPAC
HDP−Non−HS
HDP−HS
HDP−GenR
HDP−GOYA
HDP−MoBa1
HDP−MoBa2
HDP−PREDO
HDP−Viva
Study

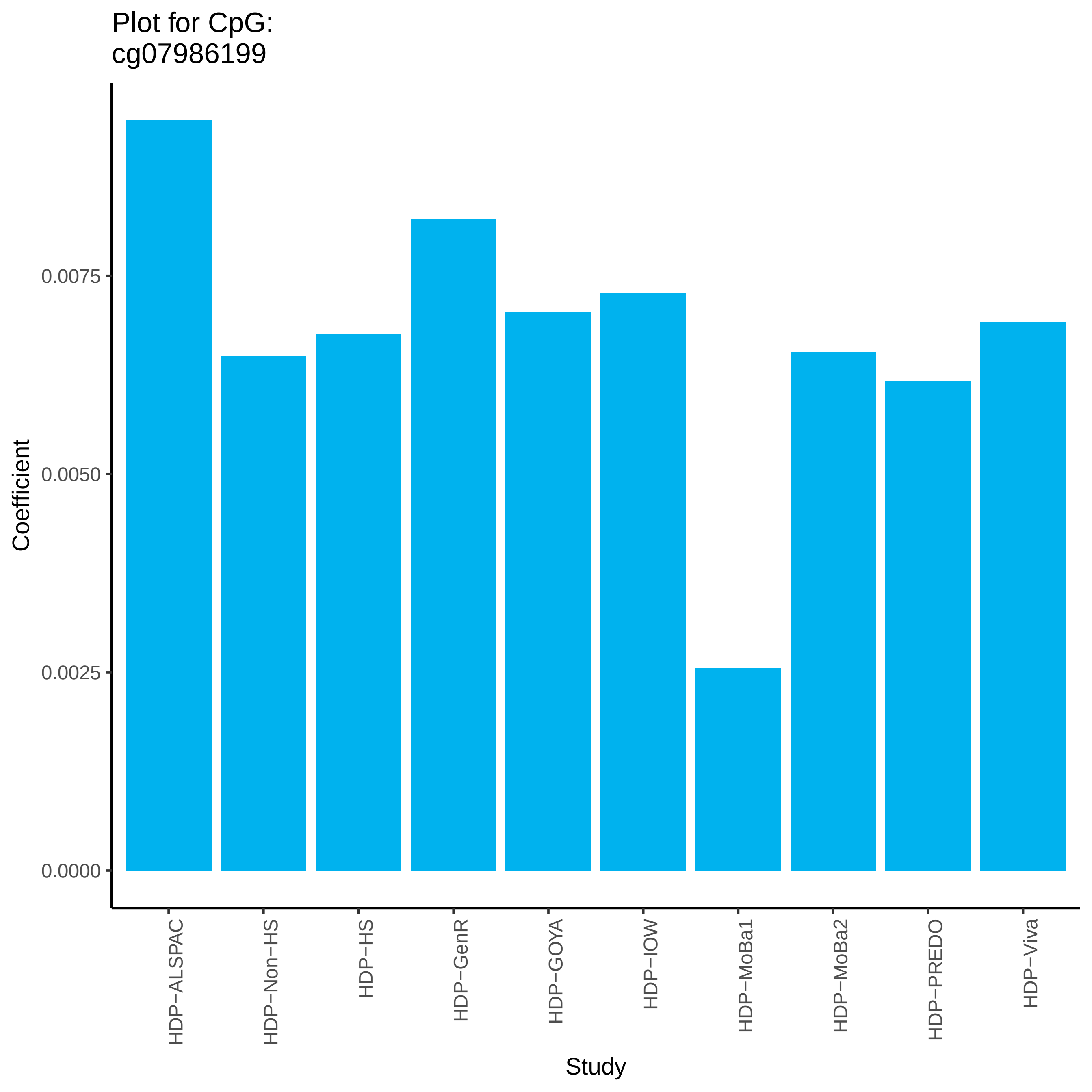

Plot for CpG:
cg07986199
Coefficient
0.0075
0.0050
0.0025
0.0000
Study
HDP−ALSPAC
HDP−Non−HS
HDP−HS
HDP−GenR
HDP−GOYA
HDP−IOW
HDP−MoBa1
HDP−MoBa2
HDP−PREDO
HDP−Viva

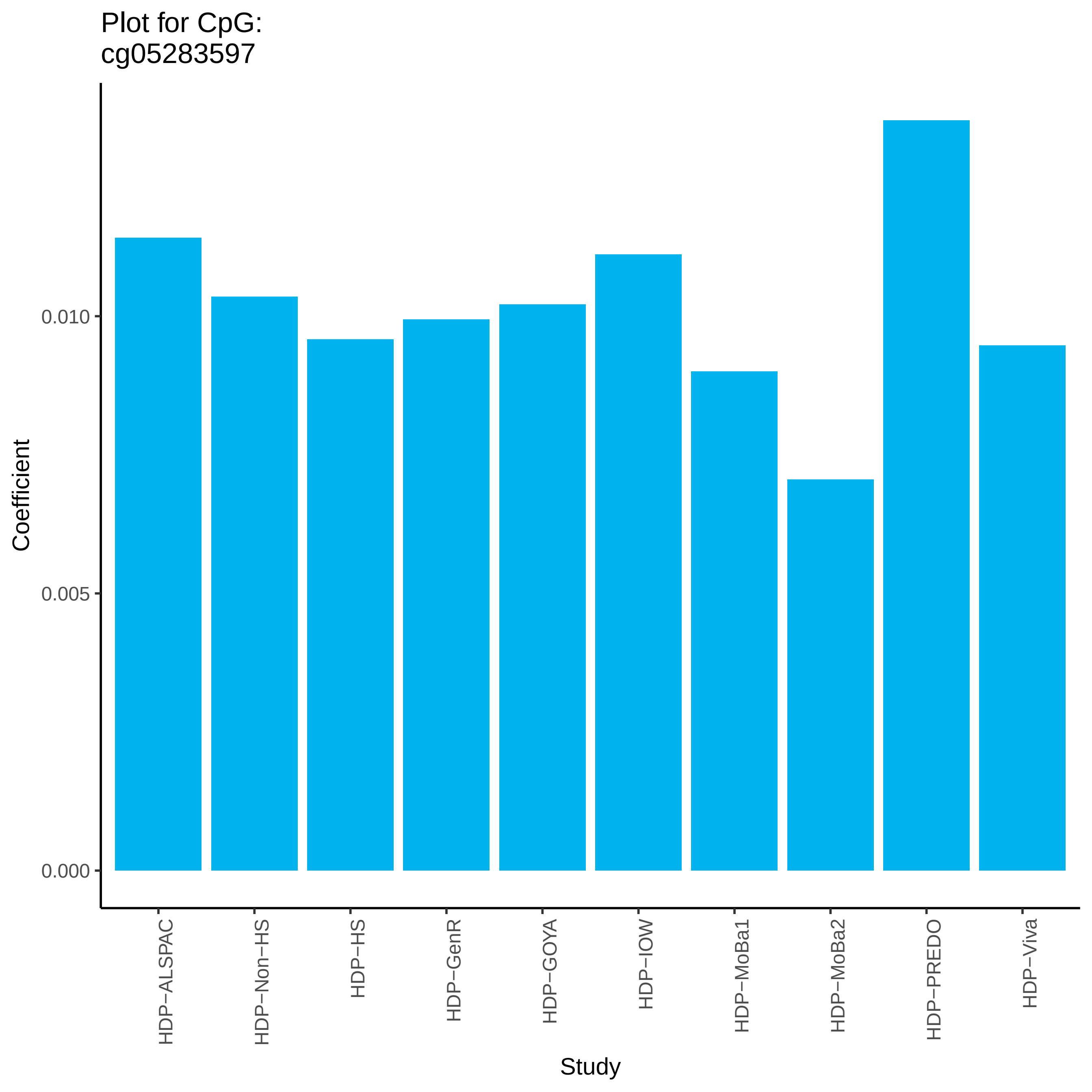

Plot for CpG:
cg05283597
Coefficient
0.010
0.005
0.000
HDP−ALSPAC
HDP−Non−HS
HDP−HS
HDP−GenR
HDP−GOYA
HDP−IOW
HDP−MoBa1
HDP−MoBa2
HDP−PREDO
HDP−Viva
Study

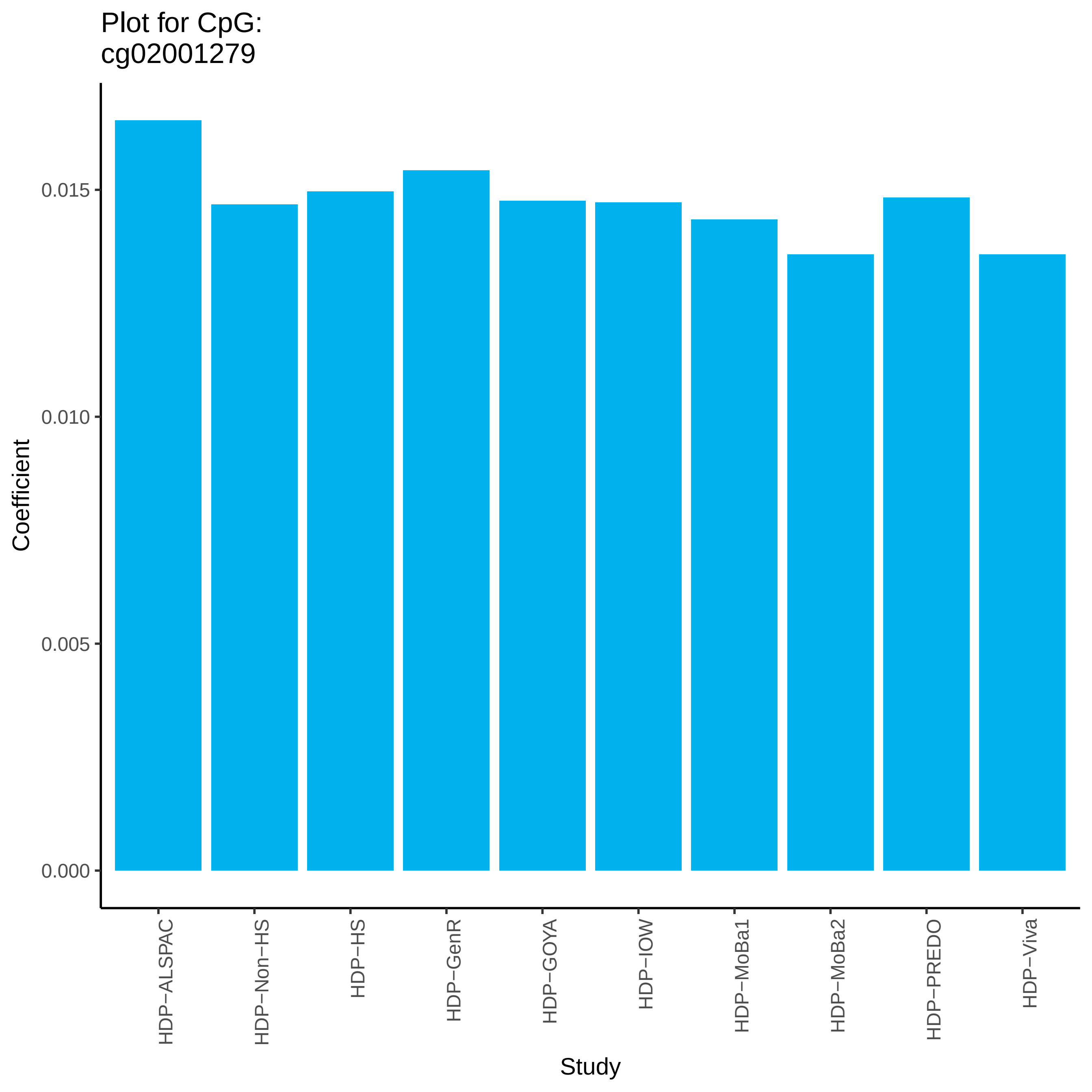
Plot for CpG:
cg02001279
Coefficient
0.015
0.010
0.005
0.000
HDP−ALSPAC
HDP−Non−HS
HDP−HS
HDP−GenR
HDP−GOYA
HDP−IOW
HDP−MoBa1
HDP−MoBa2
HDP−PREDO
HDP−Viva
Study

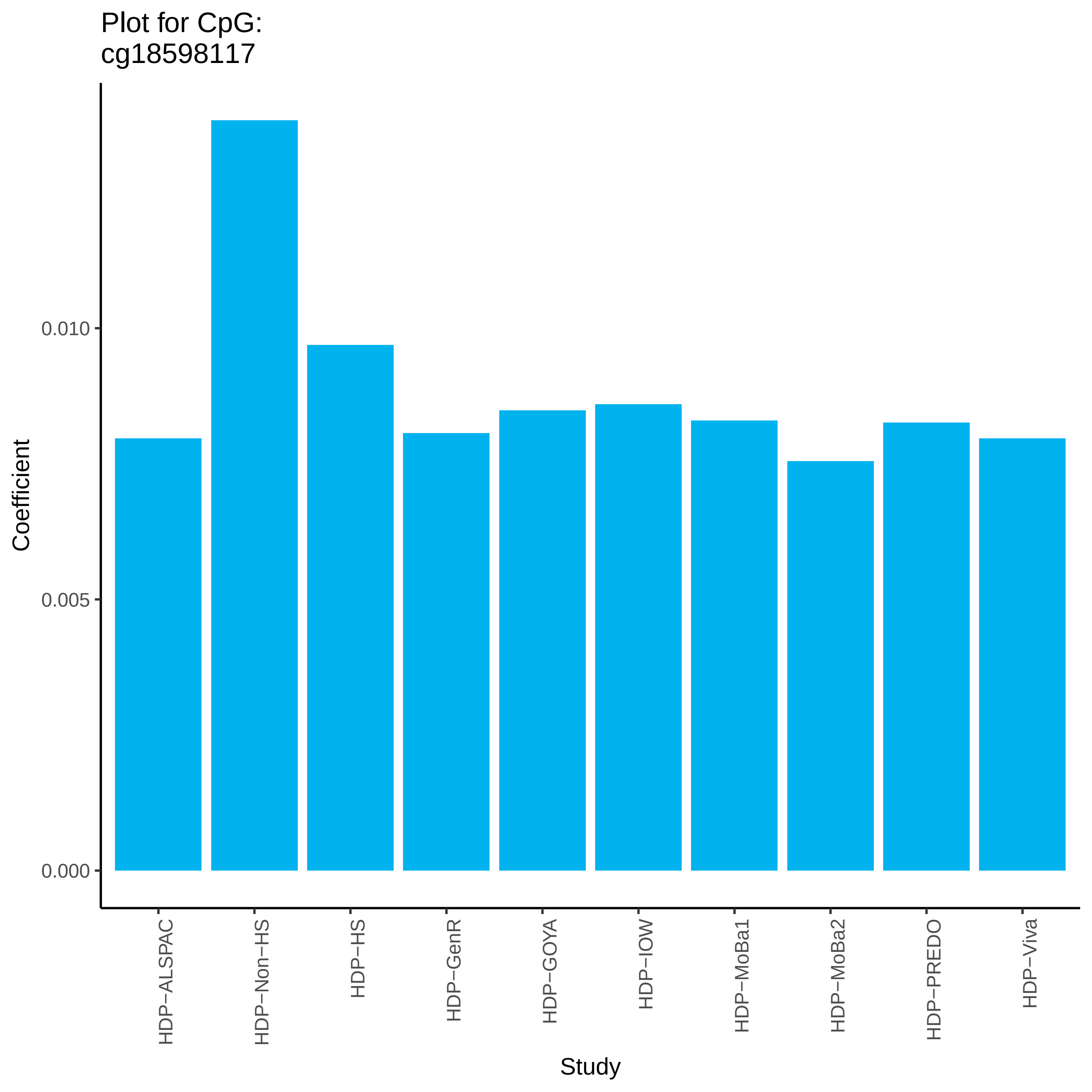

Plot for CpG:
cg18598117
Coefficient
0.010
0.005
0.000
HDP−ALSPAC
HDP−Non−HS
HDP−HS
HDP−GenR
HDP−GOYA
HDP−IOW
HDP−MoBa1
HDP−MoBa2
HDP−PREDO
HDP−Viva
Study

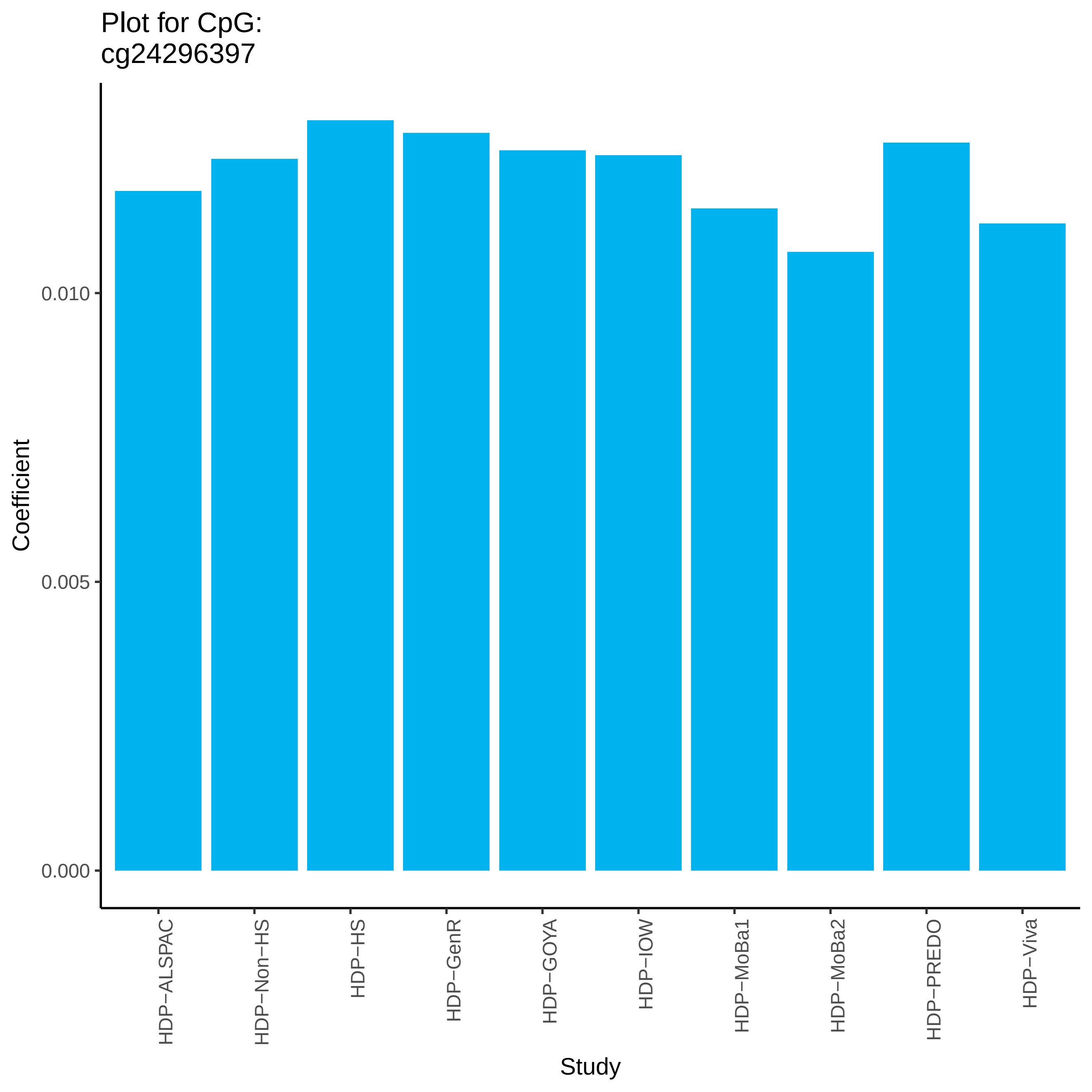
Plot for CpG:
cg24296397
Coefficient
0.010
0.005
0.000
HDP−ALSPAC
HDP−Non−HS
HDP−HS
HDP−GenR
HDP−GOYA
HDP−IOW
HDP−MoBa1
HDP−MoBa2
HDP−PREDO
HDP−Viva
Study

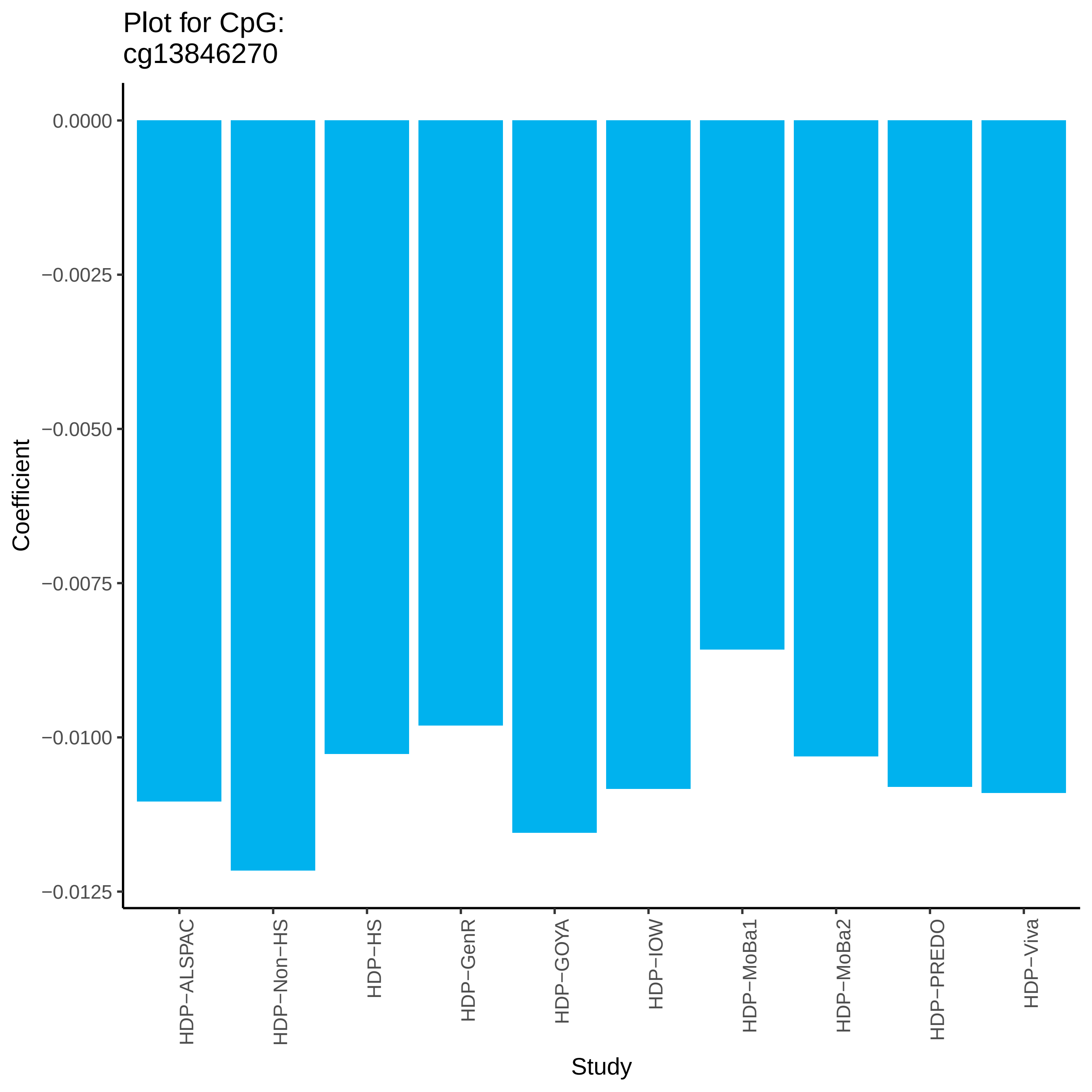

Plot for CpG:
cg13846270
Coefficient
0.0000
−0.0025
−0.0050
−0.0075
−0.0100
−0.0125
HDP−ALSPAC
HDP−Non−HS
HDP−HS
HDP−GenR
HDP−GOYA
HDP−IOW
HDP−MoBa1
HDP−MoBa2
HDP−PREDO
HDP−Viva
Study

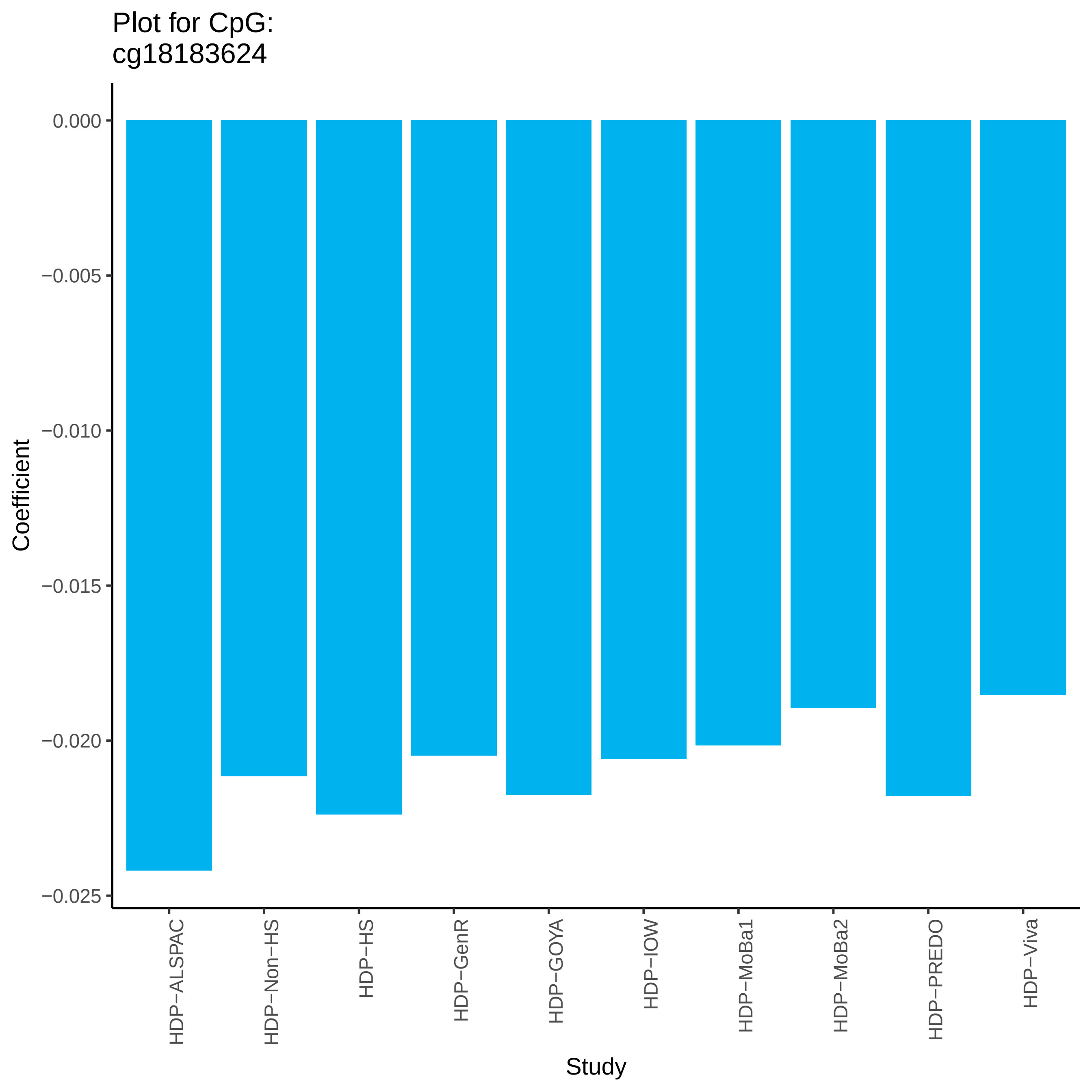
Plot for CpG:
cg18183624
Coefficient
0.000
−0.005
−0.010
−0.015
−0.020
−0.025
HDP−ALSPAC
HDP−Non−HS
HDP−HS
HDP−GenR
HDP−GOYA
HDP−IOW
HDP−MoBa1
HDP−MoBa2
HDP−PREDO
HDP−Viva
Study

Plot for CpG:
cg13110239

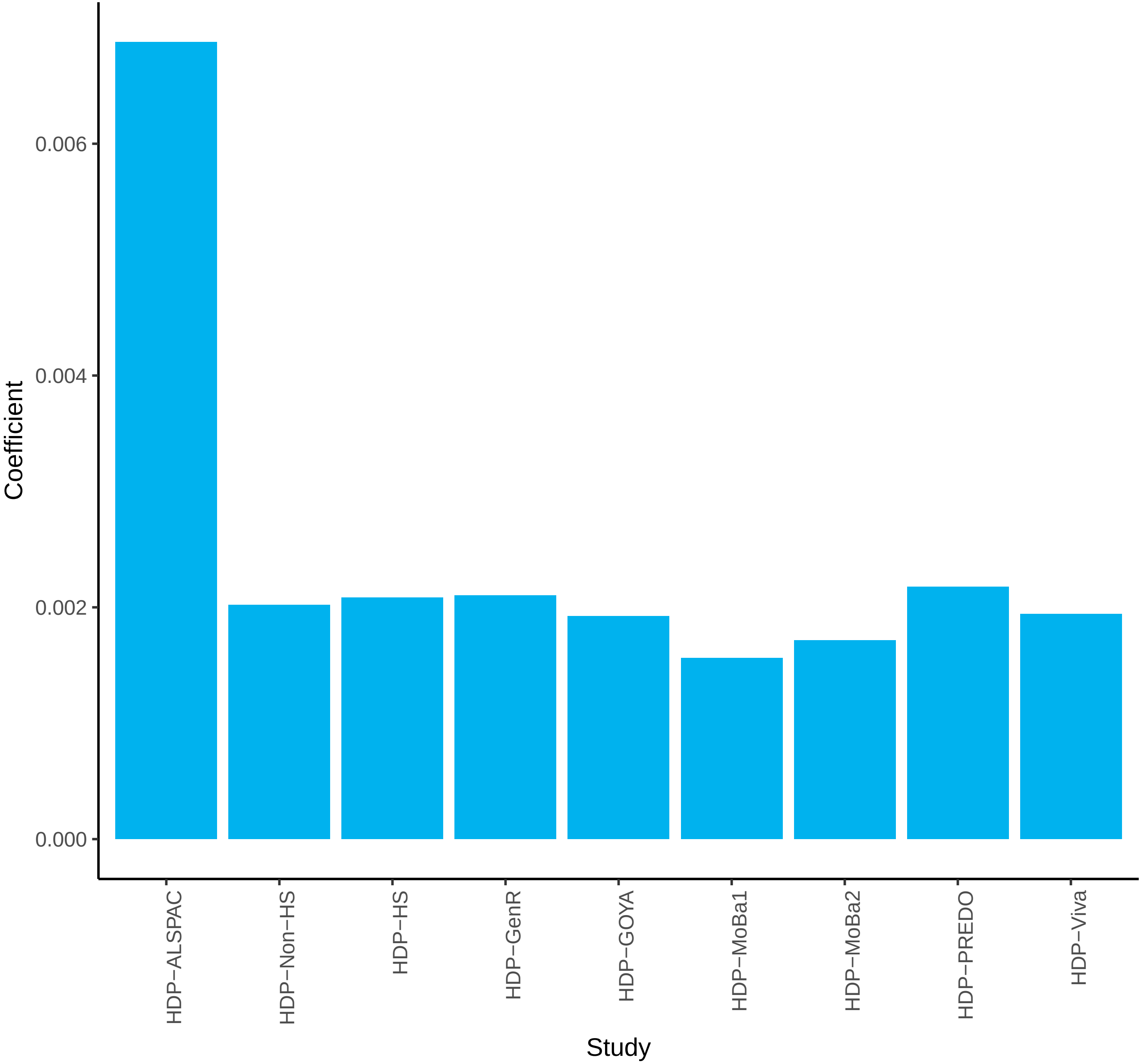

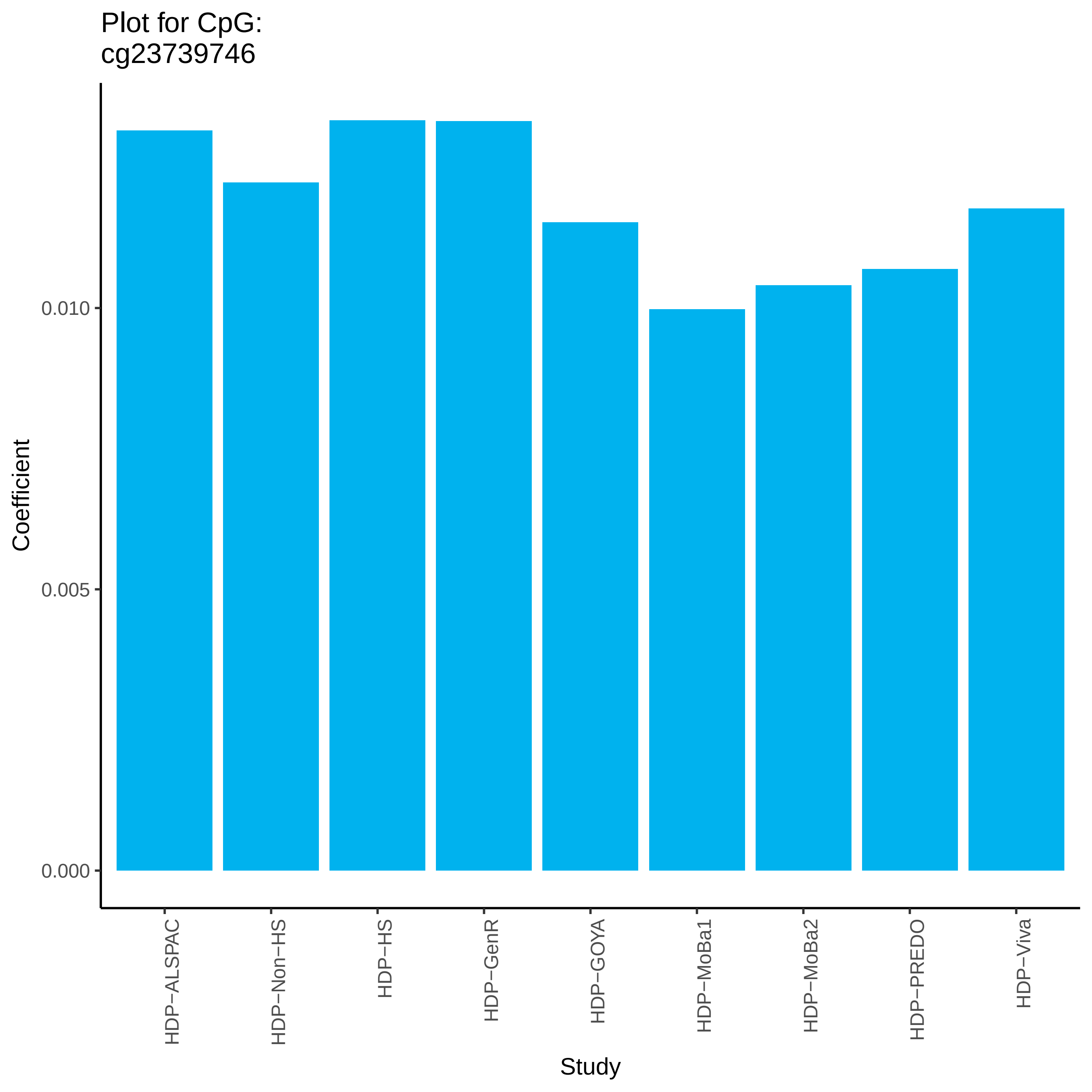
Plot for CpG:
cg23739746
Coefficient
0.010
0.005
0.000
Study
HDP−ALSPAC
HDP−Non−HS
HDP−HS
HDP−GenR
HDP−GOYA
HDP−MoBa1
HDP−MoBa2
HDP−PREDO
HDP−Viva

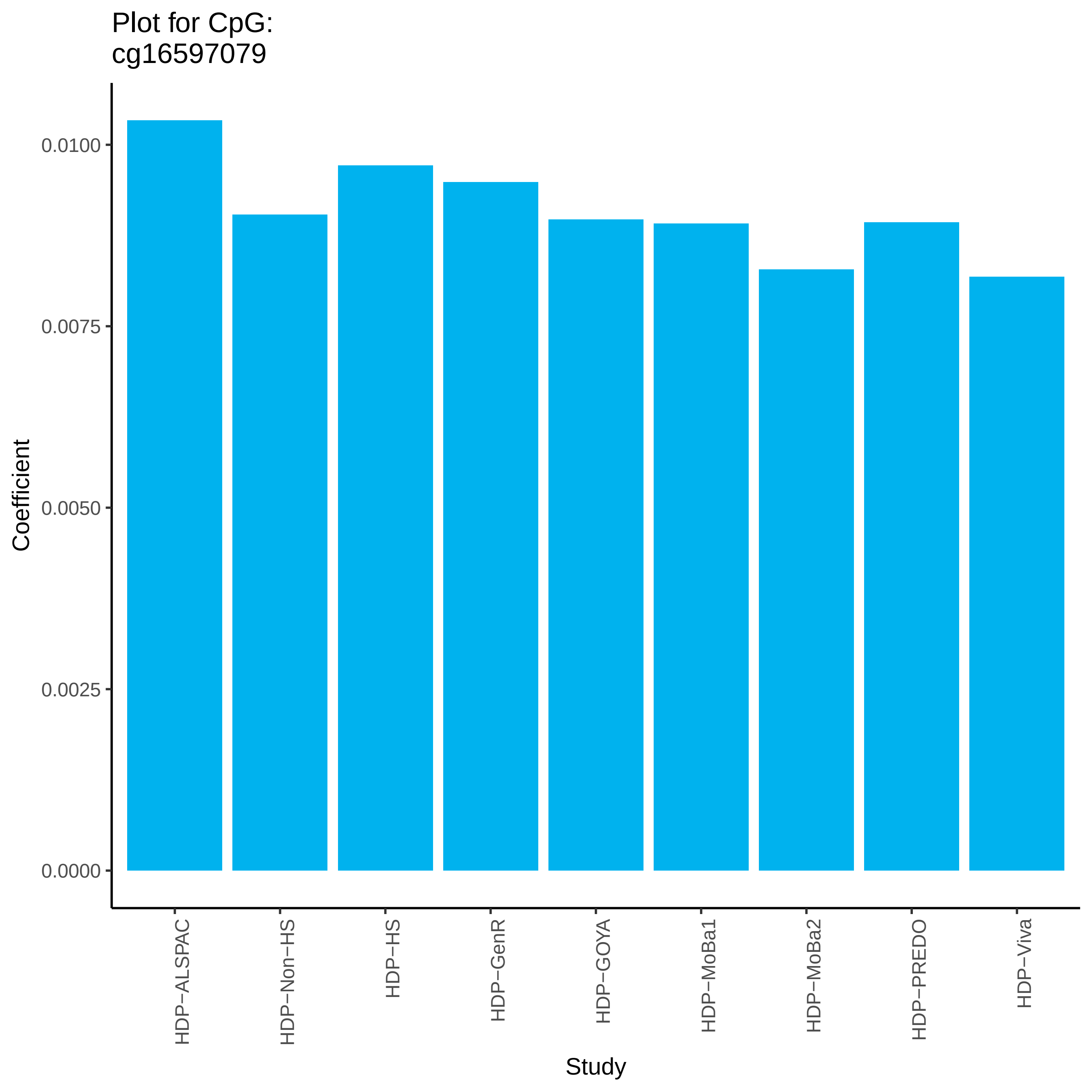

Plot for CpG:
cg16597079
Coefficient
0.0100
0.0075
0.0050
0.0025
0.0000
HDP−ALSPAC
HDP−Non−HS
HDP−HS
HDP−GenR
HDP−GOYA
HDP−MoBa1
HDP−MoBa2
HDP−PREDO
HDP−Viva
Study

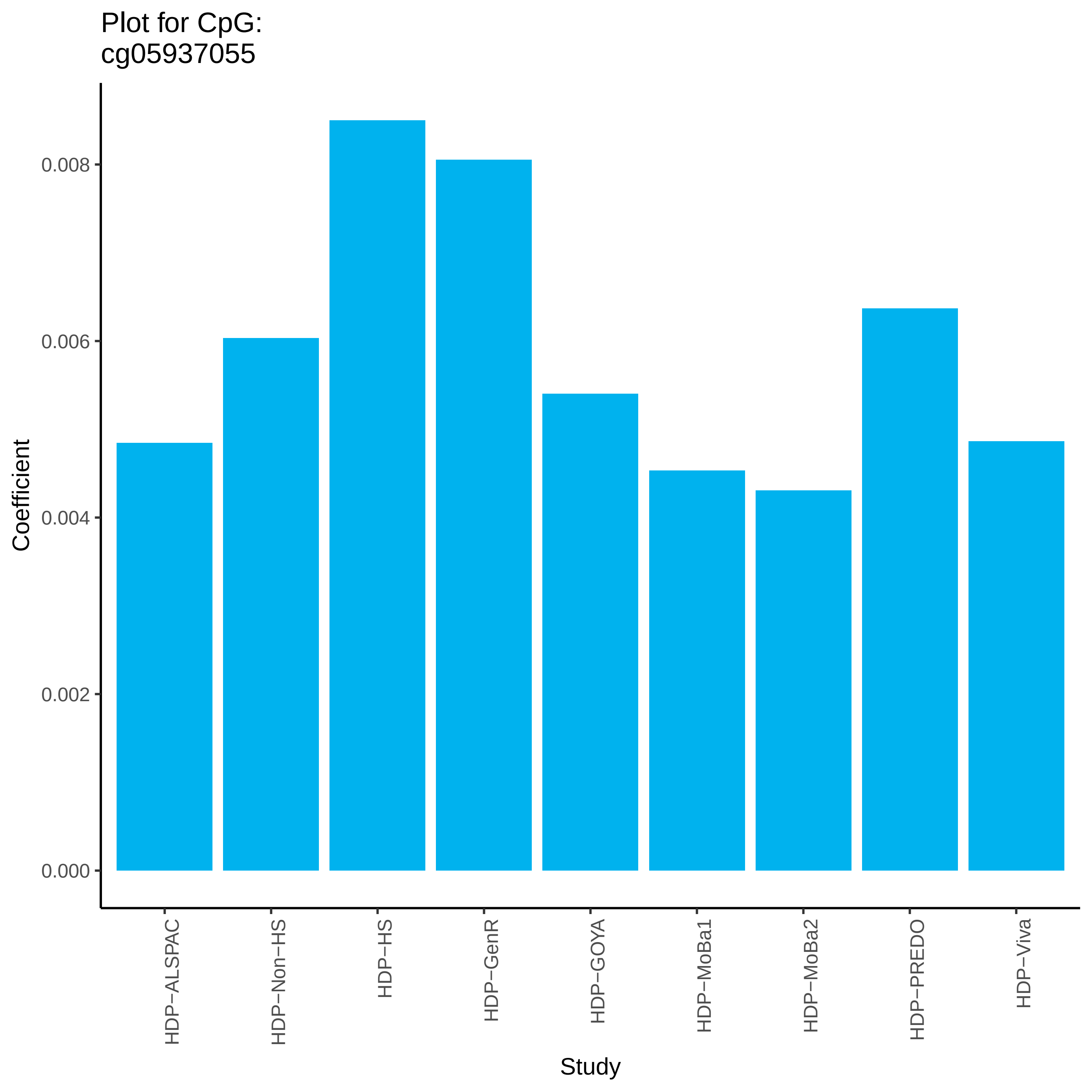

Plot for CpG:
cg05937055
Coefficient
0.000
0.002
0.004
0.006
0.008
Study
HDP−ALSPAC
HDP−Non−HS
HDP−HS
HDP−GenR
HDP−GOYA
HDP−MoBa1
HDP−MoBa2
HDP−PREDO
HDP−Viva

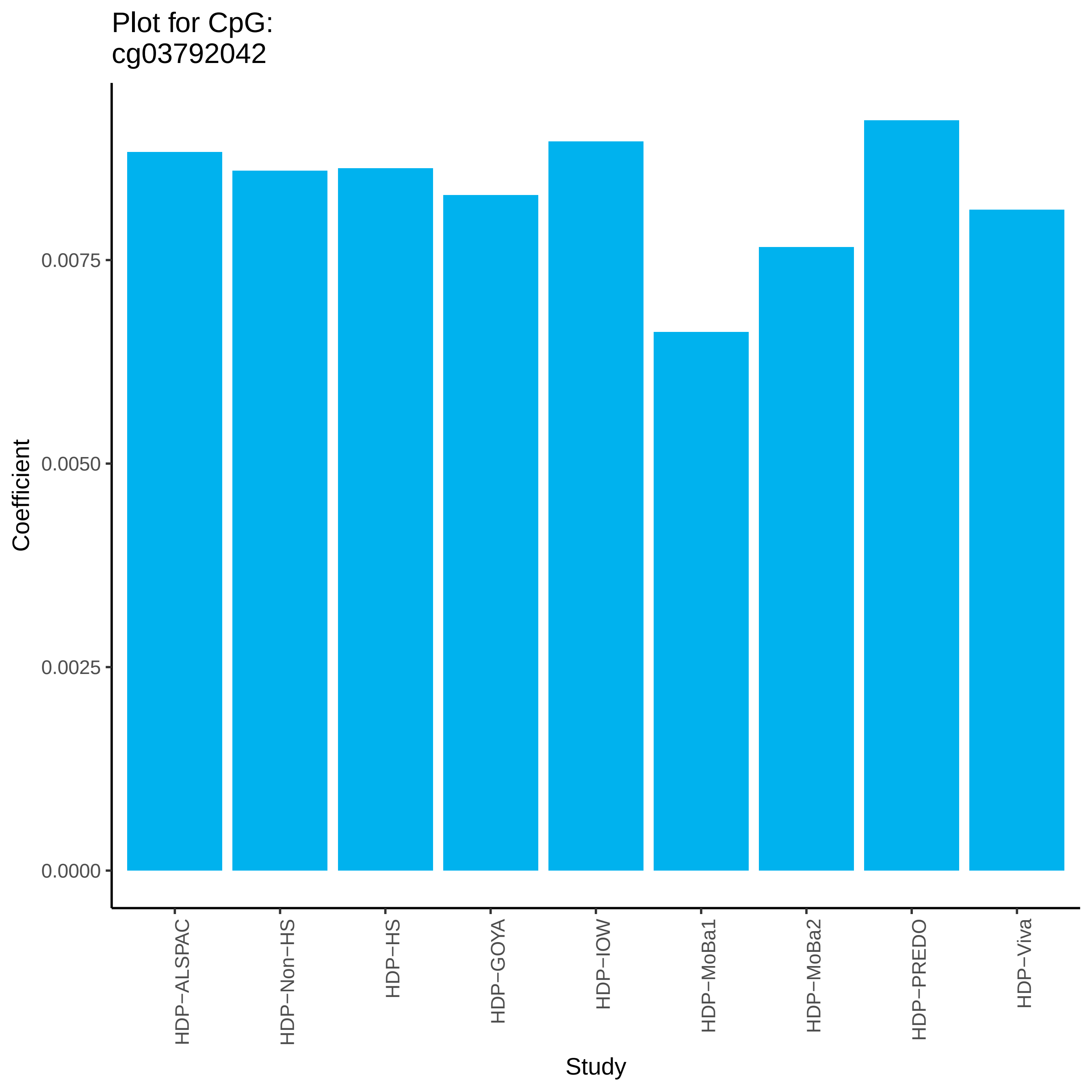

Plot for CpG:
cg03792042
Coefficient
0.0075
0.0050
0.0025
0.0000
Study
HDP−ALSPAC
HDP−Non−HS
HDP−HS
HDP−GOYA
HDP−IOW
HDP−MoBa1
HDP−MoBa2
HDP−PREDO
HDP−Viva

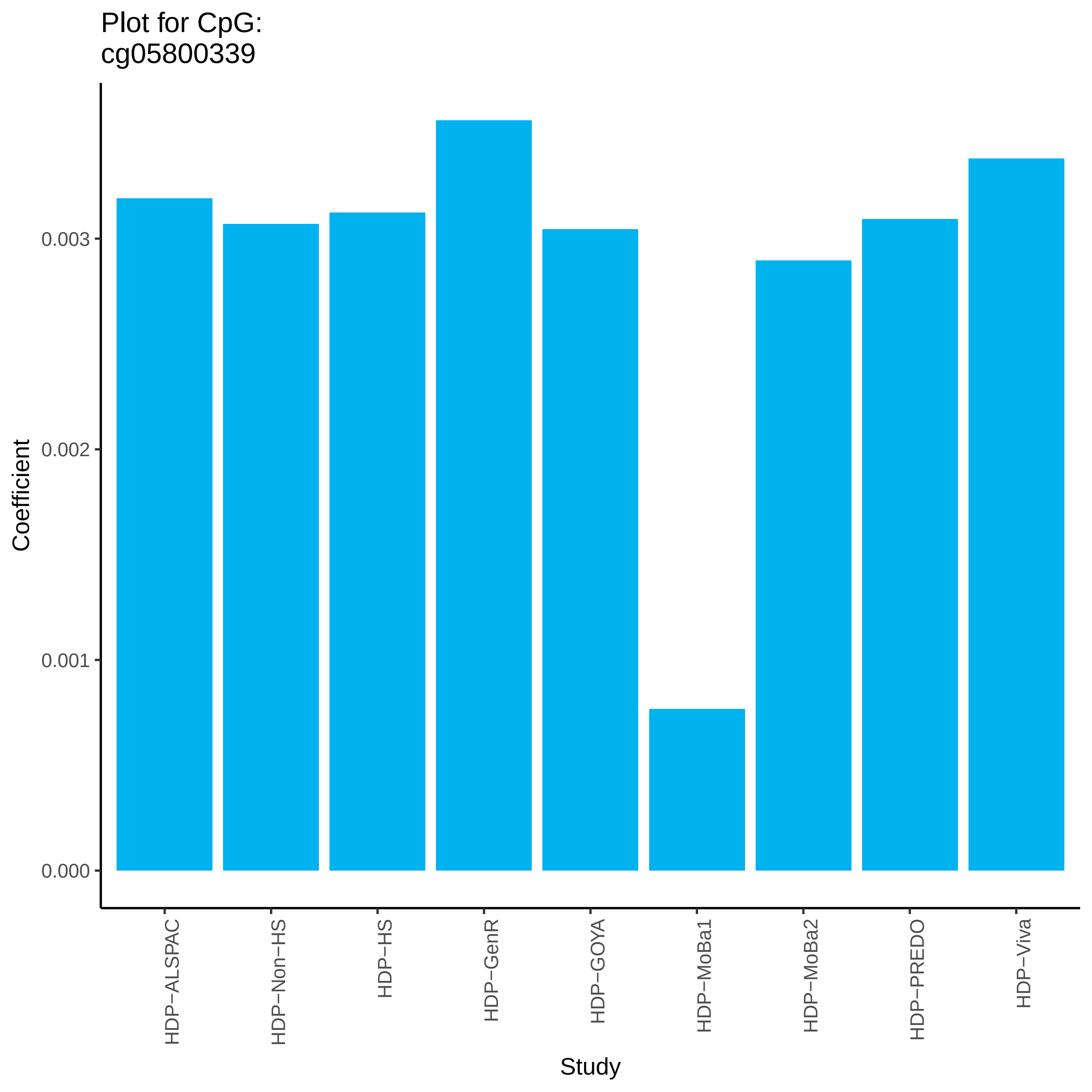
Plot for CpG:
cg05800339
Coefficient
0.003
0.002
0.001
0.000
HDP−ALSPAC
HDP−Non−HS
HDP−HS
HDP−GenR
HDP−GOYA
HDP−MoBa1
HDP−MoBa2
HDP−PREDO
HDP−Viva
Study

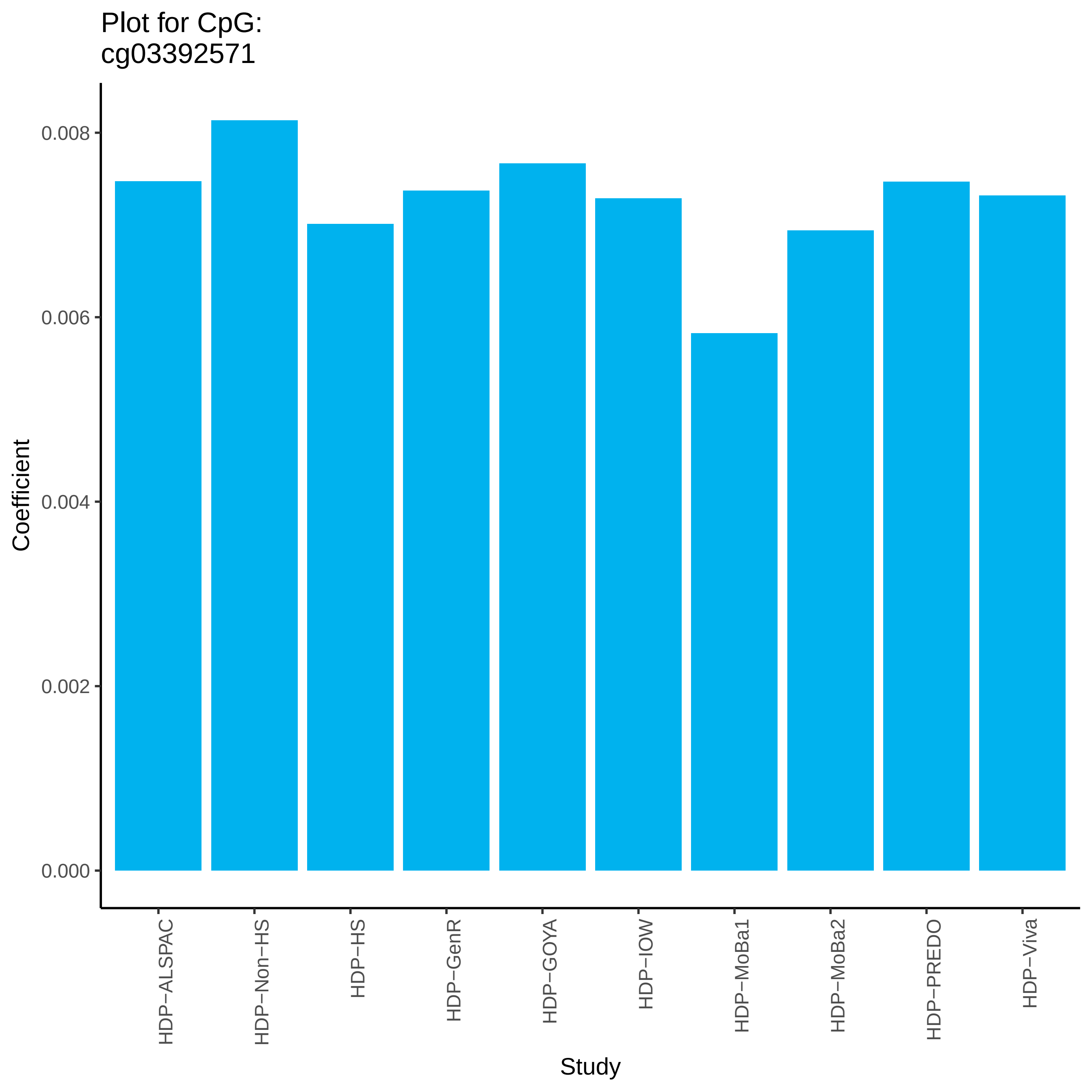
Plot for CpG:
cg03392571
Coefficient
0.000
0.002
0.004
0.006
0.008
Study
HDP−ALSPAC
HDP−Non−HS
HDP−HS
HDP−GenR
HDP−GOYA
HDP−IOW
HDP−MoBa1
HDP−MoBa2
HDP−PREDO
HDP−Viva

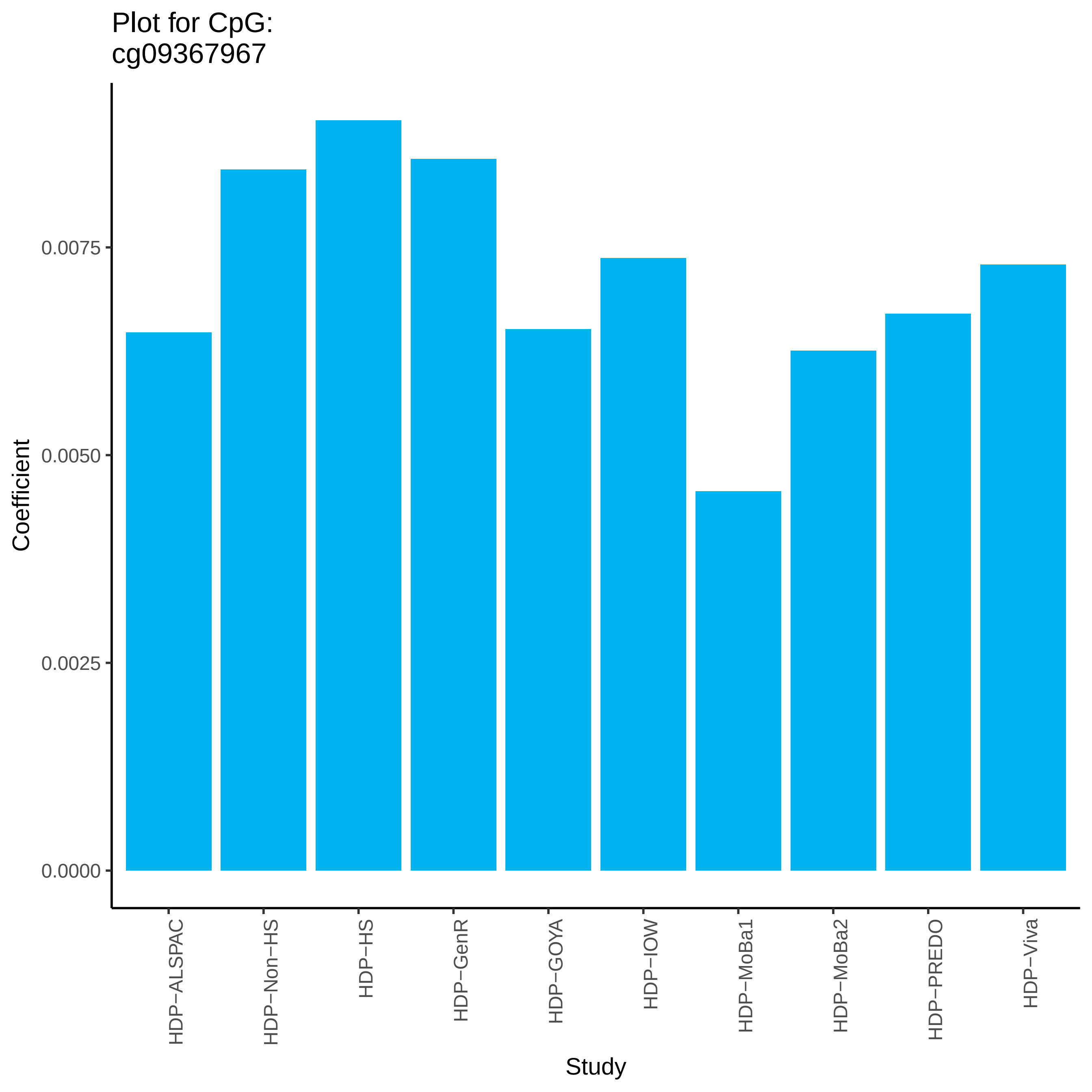
Plot for CpG:
cg09367967
Coefficient
0.0075
0.0050
0.0025
0.0000
HDP−ALSPAC
HDP−Non−HS
HDP−HS
HDP−GenR
HDP−GOYA
HDP−IOW
HDP−MoBa1
HDP−MoBa2
HDP−PREDO
HDP−Viva
Study

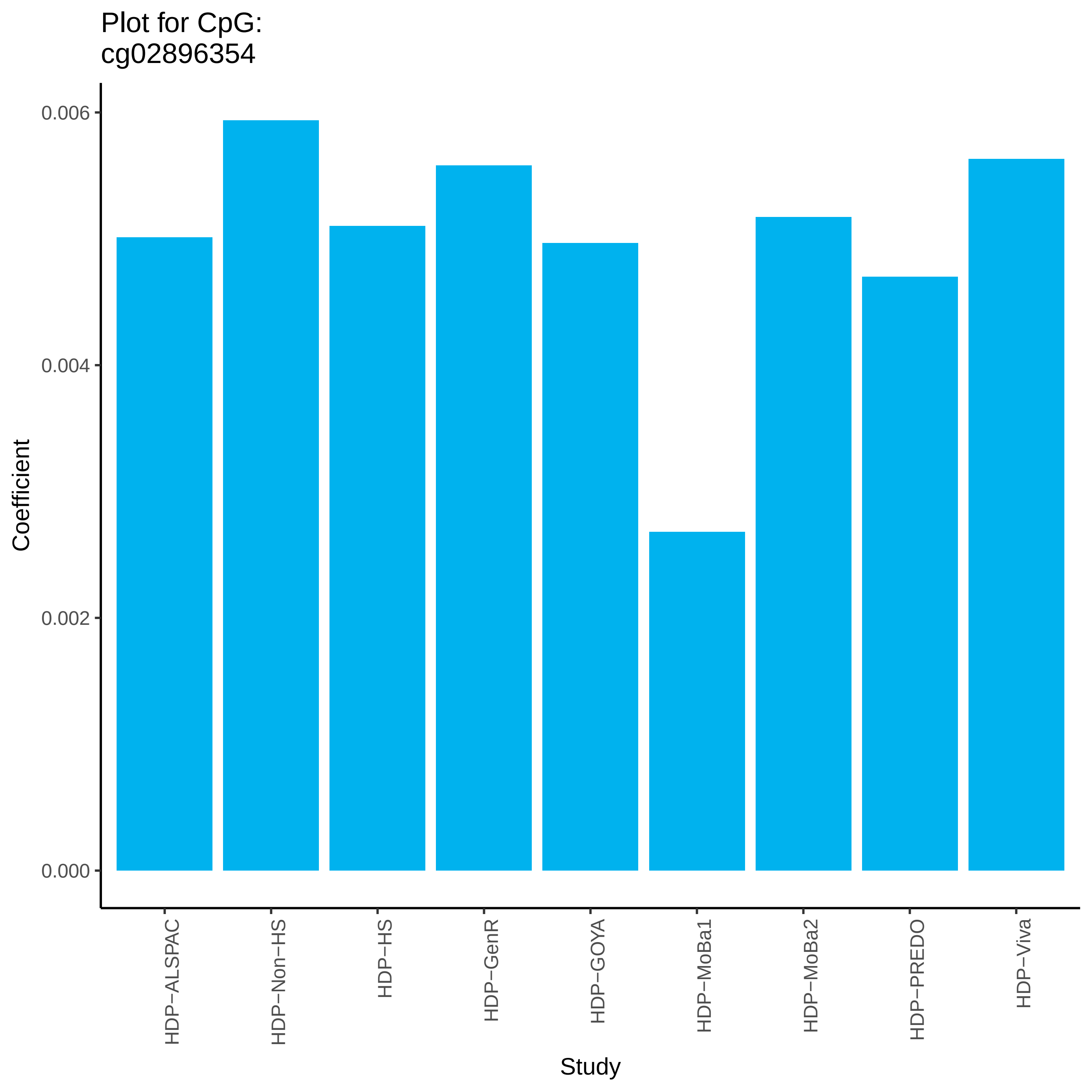

Plot for CpG:
cg02896354
Coefficient
0.006
0.004
0.002
0.000
HDP−ALSPAC
HDP−Non−HS
HDP−HS
HDP−GenR
HDP−GOYA
HDP−MoBa1
HDP−MoBa2
HDP−PREDO
HDP−Viva
Study

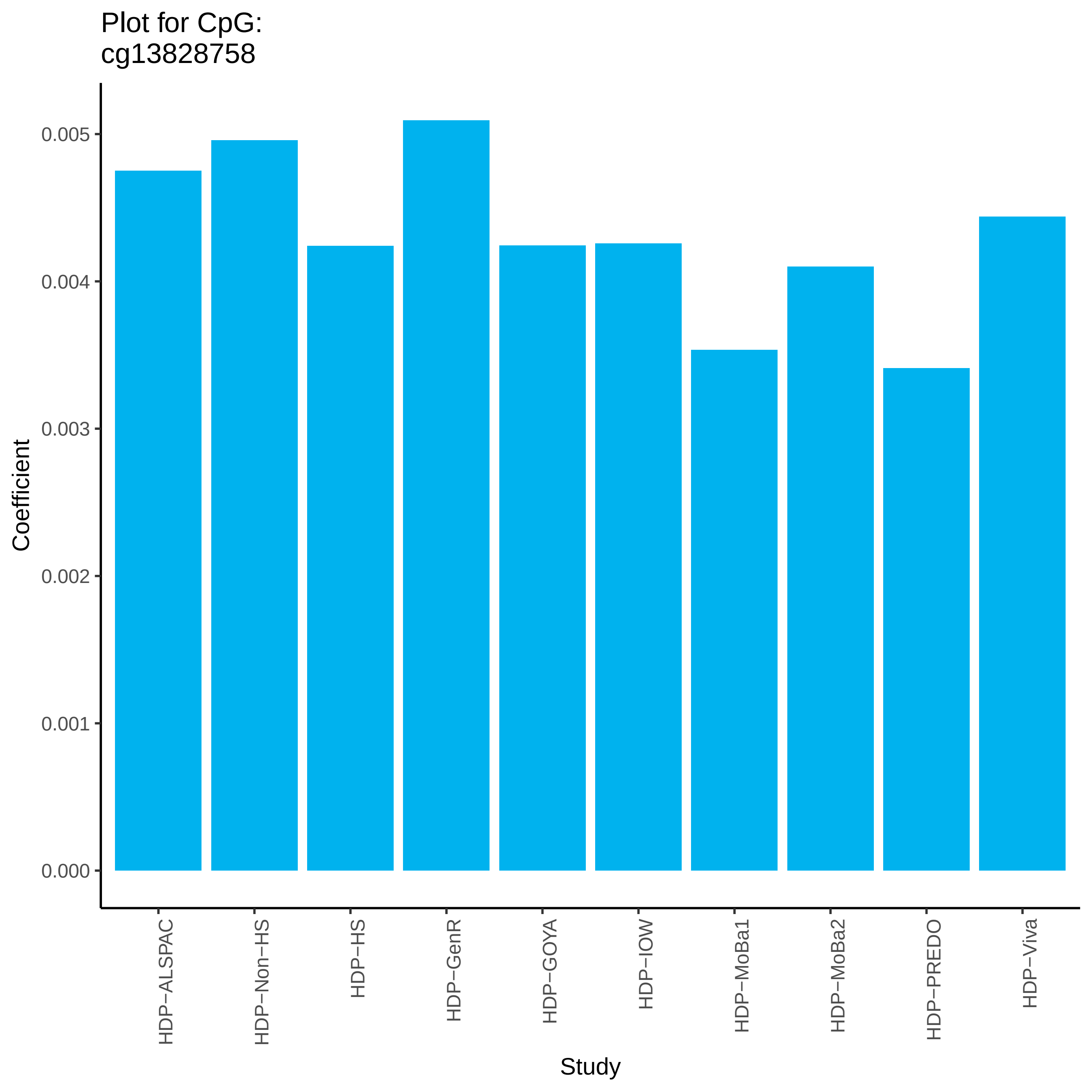

Plot for CpG:
cg13828758
Coefficient
0.005
0.004
0.003
0.002
0.001
0.000
HDP−ALSPAC
HDP−Non−HS
HDP−HS
HDP−GenR
HDP−GOYA
HDP−IOW
HDP−MoBa1
HDP−MoBa2
HDP−PREDO
HDP−Viva
Study

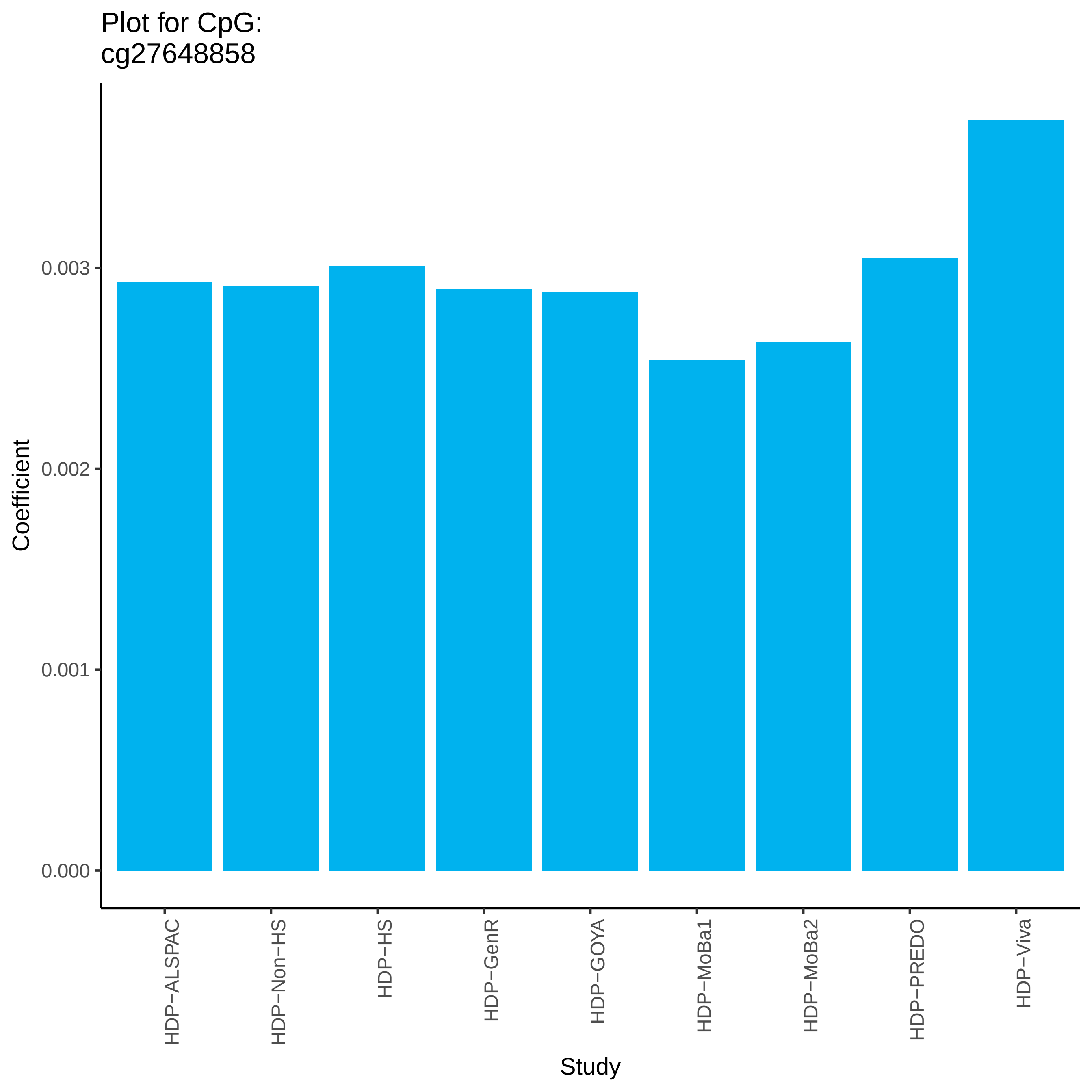

Plot for CpG:
cg27648858
Coefficient
0.003
0.002
0.001
0.000
HDP−ALSPAC
HDP−Non−HS
HDP−HS
HDP−GenR
HDP−GOYA
HDP−MoBa1
HDP−MoBa2
HDP−PREDO
HDP−Viva
Study

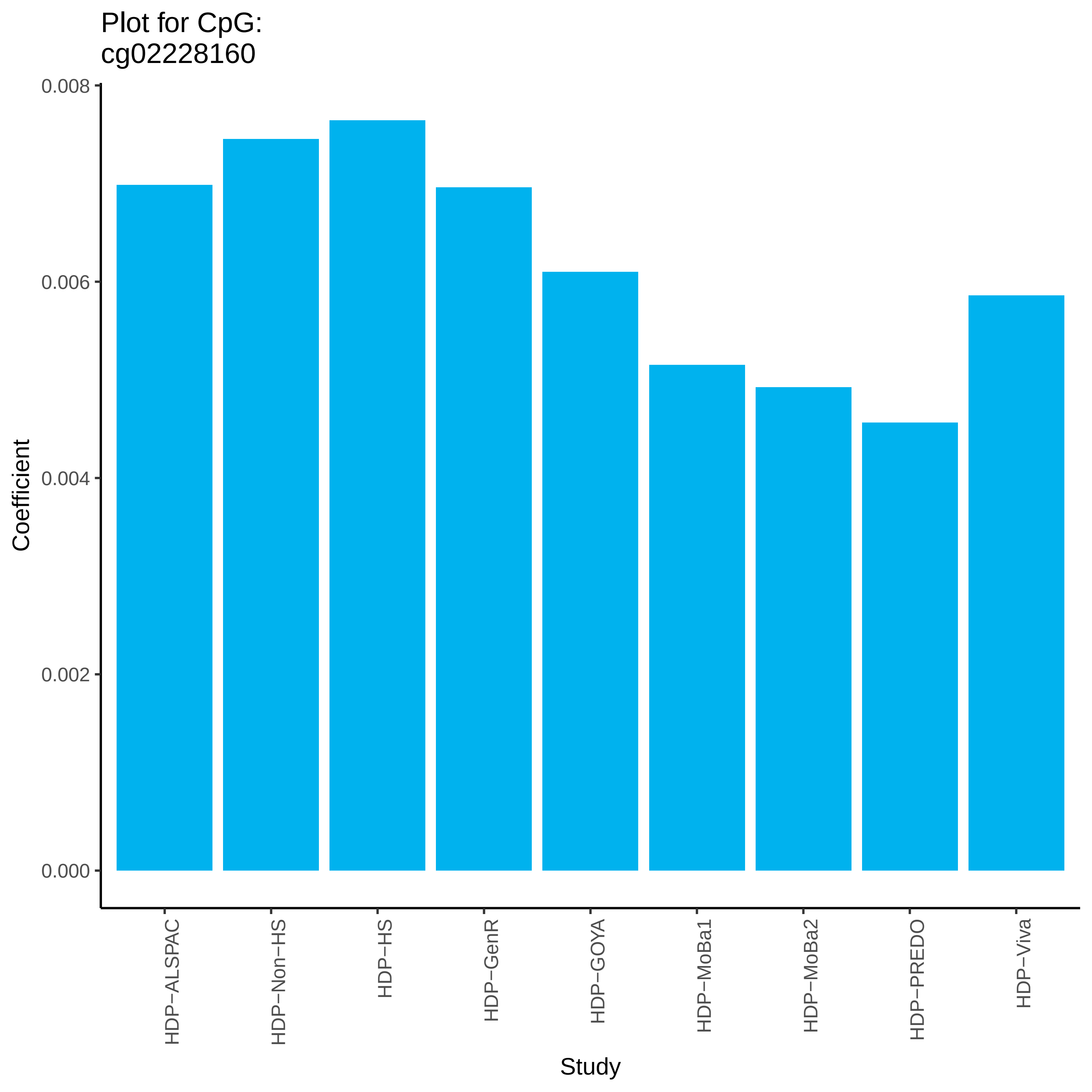
Plot for CpG:
cg02228160
Coefficient
0.008
0.006
0.004
0.002
0.000
HDP−ALSPAC
HDP−Non−HS
HDP−HS
HDP−GenR
HDP−GOYA
HDP−MoBa1
HDP−MoBa2
HDP−PREDO
HDP−Viva
Study

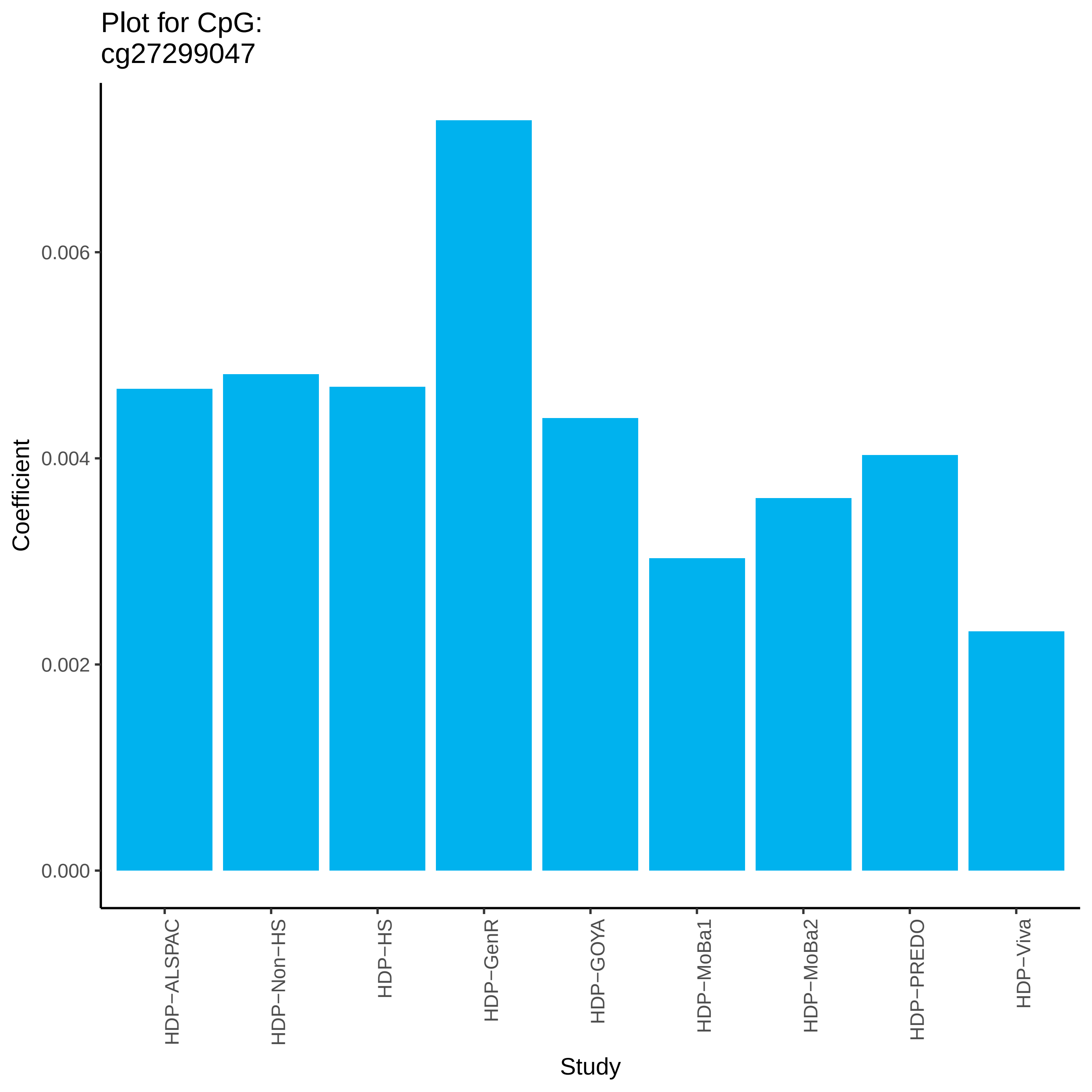
Plot for CpG:
cg27299047
Coefficient
0.006
0.004
0.002
0.000
HDP−ALSPAC
HDP−Non−HS
HDP−HS
HDP−GenR
HDP−GOYA
HDP−MoBa1
HDP−MoBa2
HDP−PREDO
HDP−Viva
Study

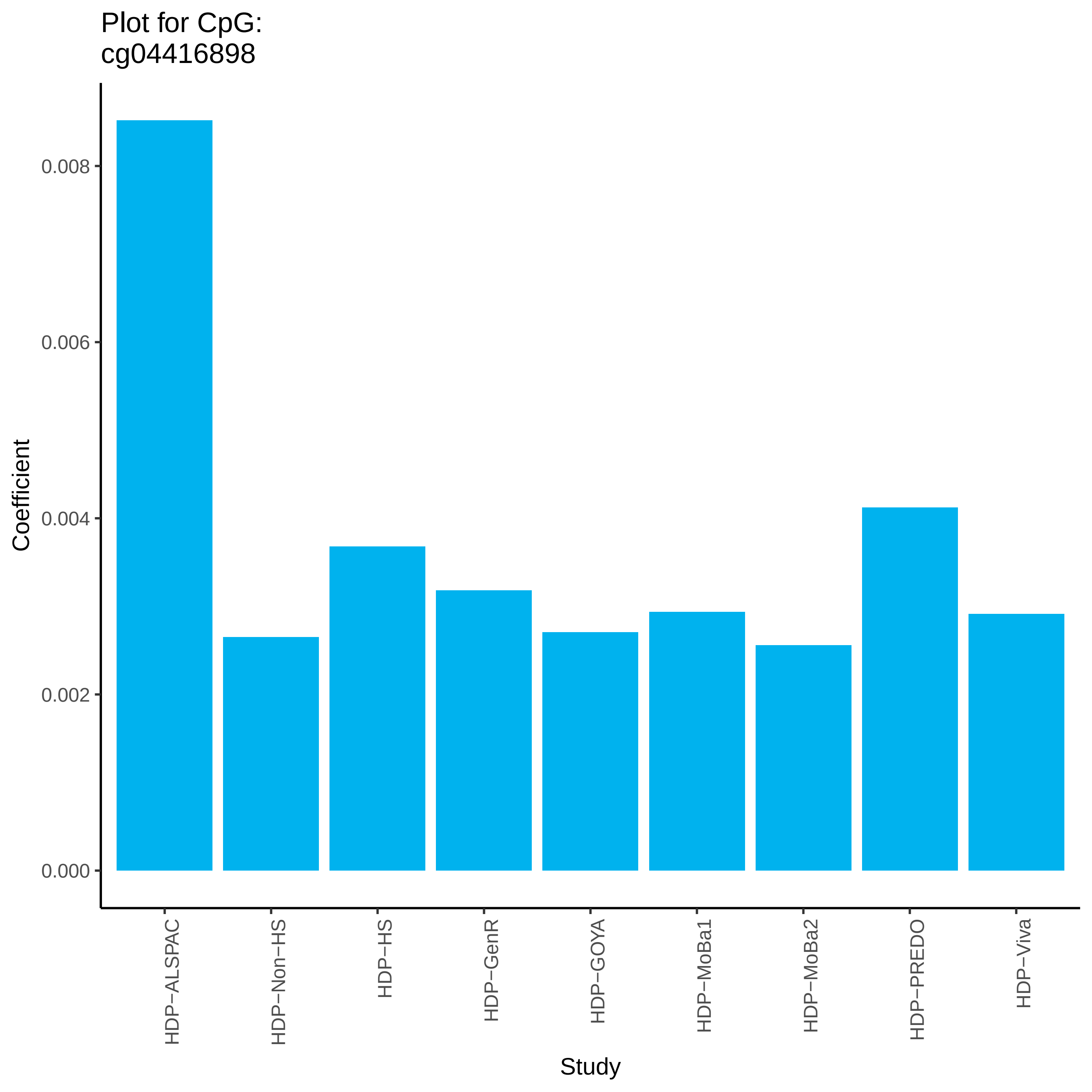
Plot for CpG:
cg04416898
Coefficient
0.008
0.006
0.004
0.002
0.000
HDP−ALSPAC
HDP−Non−HS
HDP−HS
HDP−GenR
HDP−GOYA
HDP−MoBa1
HDP−MoBa2
HDP−PREDO
HDP−Viva
Study

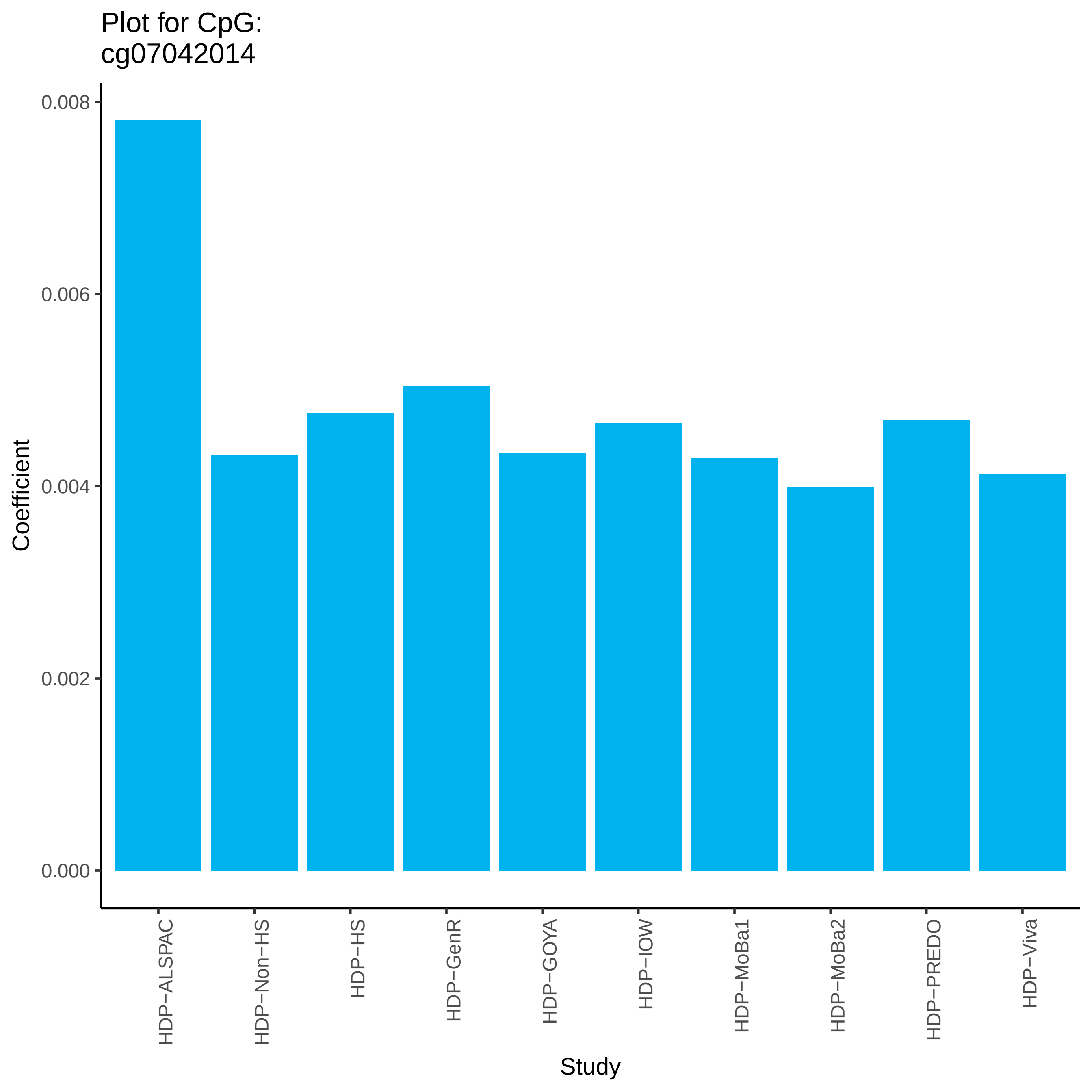
Plot for CpG:
cg07042014
Coefficient
0.000
0.002
0.004
0.006
0.008
Study
HDP−ALSPAC
HDP−Non−HS
HDP−HS
HDP−GenR
HDP−GOYA
HDP−IOW
HDP−MoBa1
HDP−MoBa2
HDP−PREDO
HDP−Viva

Plot for CpG:
cg05591701

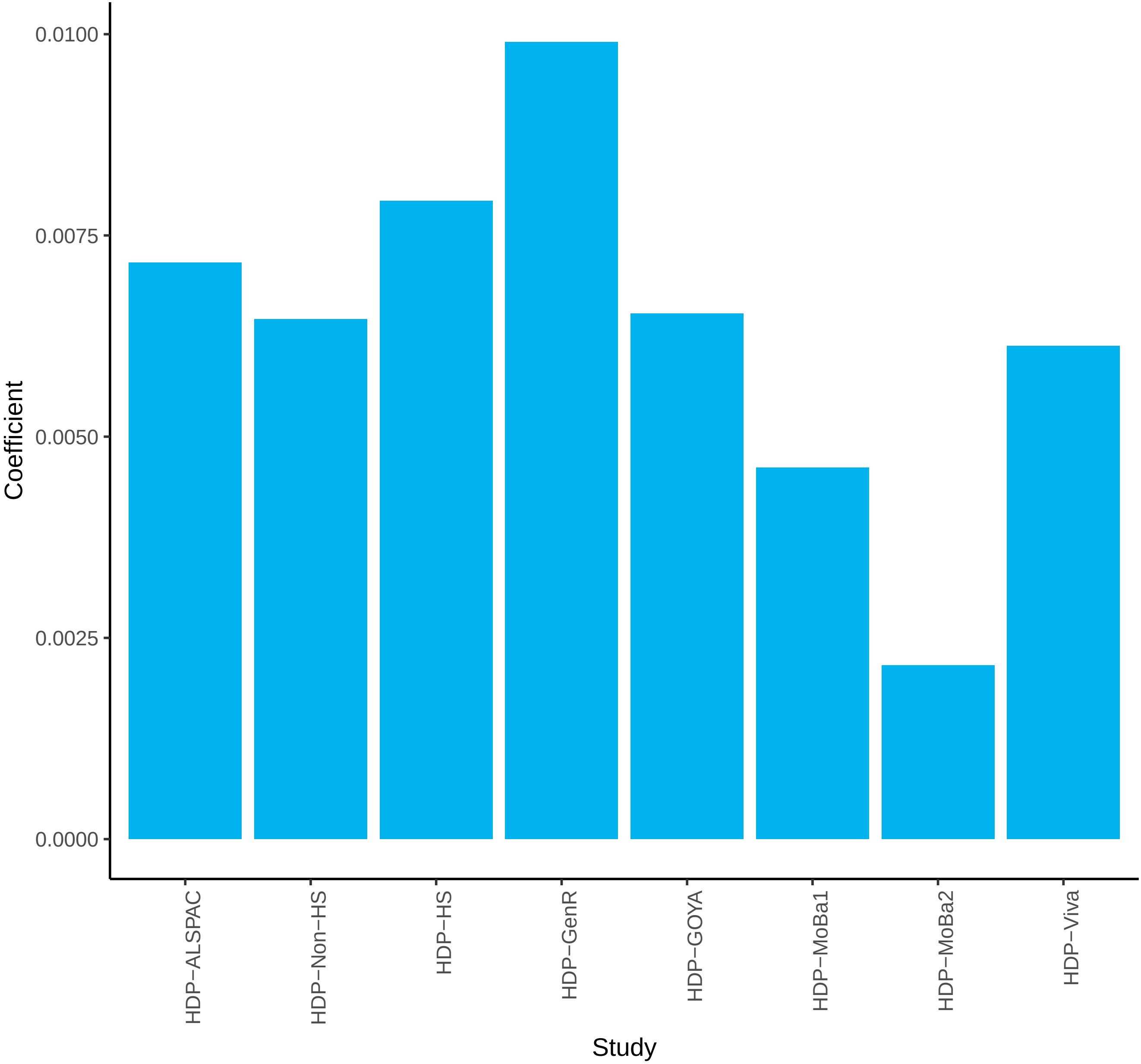

Plot for CpG:
cg23469878

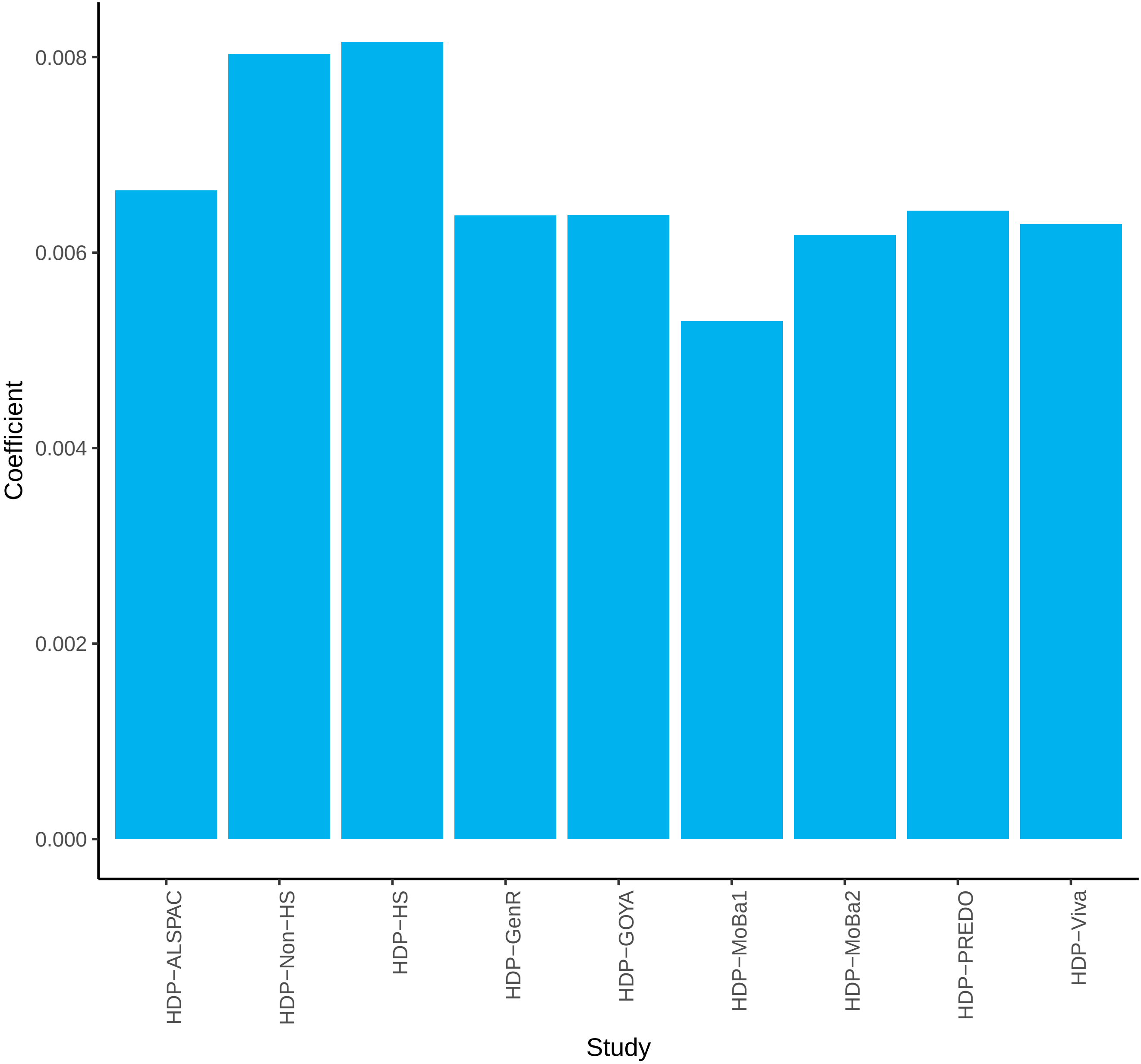

Plot for CpG:
cg00531137

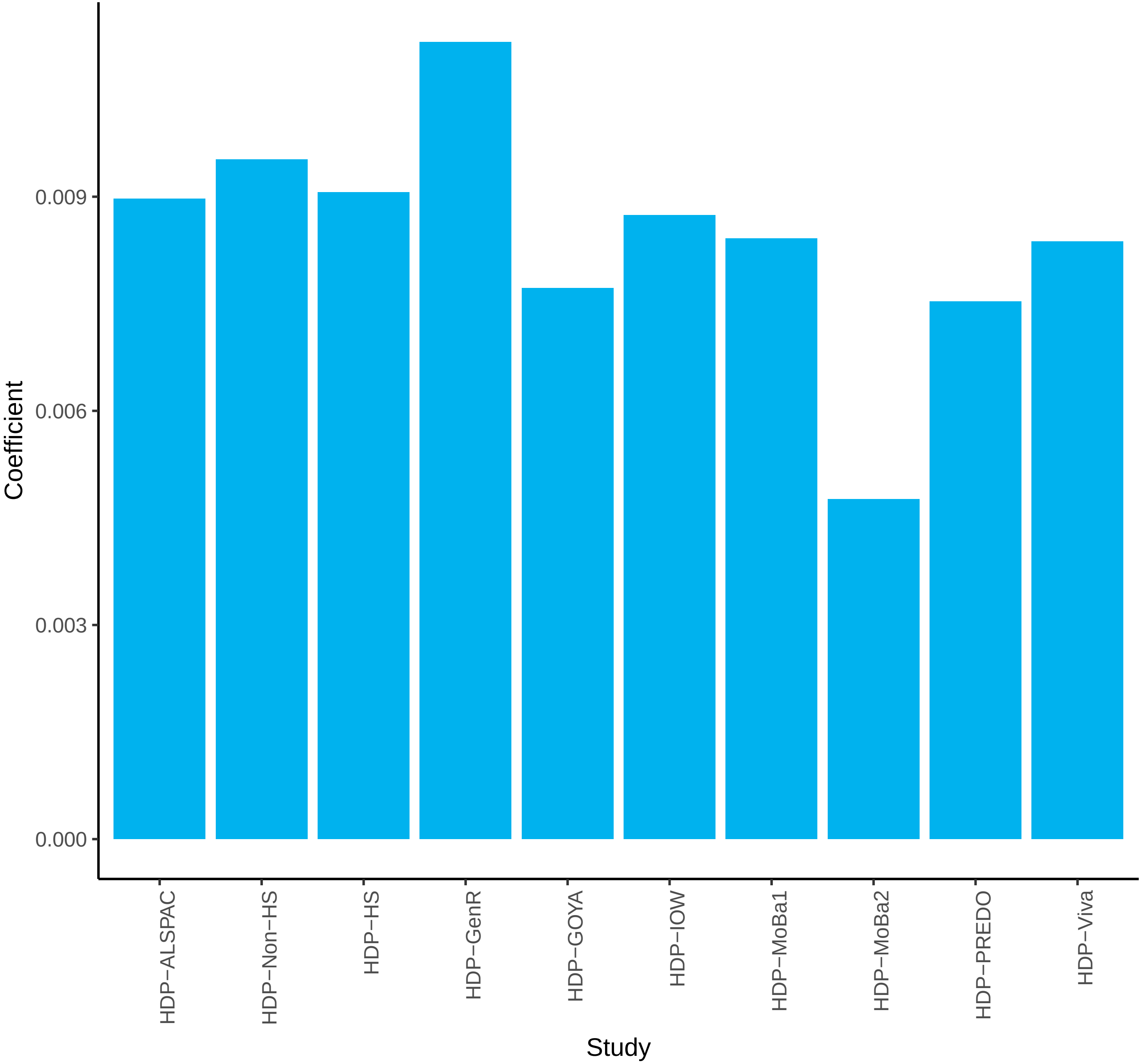

Supplement: Supplementary file 4 [file hyp-74-375-s004.pdf]
